# Plasma metabolomic and lipidomic profiles accurately classify mothers of children with congenital heart disease: an observational study

**DOI:** 10.1007/s11306-024-02129-8

**Published:** 2024-07-02

**Authors:** Stuart Mires, Eduardo Sommella, Fabrizio Merciai, Emanuela Salviati, Vicky Caponigro, Manuela Giovanna Basilicata, Federico Marini, Pietro Campiglia, Mai Baquedano, Tim Dong, Clare Skerritt, Kelly-Ann Eastwood, Massimo Caputo

**Affiliations:** 1 Translational Health Sciences, University of Bristol, Bristol, UK; 2 University Hospitals Bristol and Weston NHS Foundation Trust, Bristol, UK; 3 Department of Pharmacy, University of Salerno, Salerno, Italy; 4 Department of Advanced Medical and Surgical Sciences, University of Campania “Luigi Vanvitelli”, Naples, Italy; 5 Department of Chemistry, University of Rome, Rome, Italy

**Keywords:** ALSPAC, Congenital heart disease, Metabolomics, Prediction models, Risk factors

## Abstract

**Introduction:**

Congenital heart disease (CHD) is the most common congenital anomaly, representing a significant global disease burden. Limitations exist in our understanding of aetiology, diagnostic methodology and screening, with metabolomics offering promise in addressing these.

**Objective:**

To evaluate maternal metabolomics and lipidomics in prediction and risk factor identification for childhood CHD.

**Methods:**

We performed an observational study in mothers of children with CHD following pregnancy, using untargeted plasma metabolomics and lipidomics by ultrahigh performance liquid chromatography-high resolution mass spectrometry (UHPLC-HRMS). 190 cases (157 mothers of children with structural CHD (sCHD); 33 mothers of children with genetic CHD (gCHD)) from the children OMACp cohort and 162 controls from the ALSPAC cohort were analysed. CHD diagnoses were stratified by severity and clinical classifications. Univariate, exploratory and supervised chemometric methods were used to identify metabolites and lipids distinguishing cases and controls, alongside predictive modelling.

**Results:**

499 metabolites and lipids were annotated and used to build PLS-DA and SO-CovSel-LDA predictive models to accurately distinguish sCHD and control groups. The best performing model had an sCHD test set mean accuracy of 94.74% (sCHD test group sensitivity 93.33%; specificity 96.00%) utilising only 11 analytes. Similar test performances were seen for gCHD. Across best performing models, 37 analytes contributed to performance including amino acids, lipids, and nucleotides.

**Conclusions:**

Here, maternal metabolomic and lipidomic analysis has facilitated the development of sensitive risk prediction models classifying mothers of children with CHD. Metabolites and lipids identified offer promise for maternal risk factor profiling, and understanding of CHD pathogenesis in the future.

**Supplementary Information:**

The online version contains supplementary material available at 10.1007/s11306-024-02129-8.

## Introduction

Congenital heart disease (CHD) is defined as a ‘structural developmental anomaly of the heart or great vessels (Jacobs et al., 2021).’ It is the most common congenital anomaly, with a reported prevalence of 0.63–0.8% of total births in the UK and Europe (European Commission, 2019; Public Health England, 2019). CHD is a heterogeneous group of conditions often described using anatomical, clinical and severity classification systems (EUROCAT, 2013; Jacobs et al., 2021). Critical or severe CHD are defined as requiring intervention in the first year of life, representing 20–25% of cases (Bakker et al., 2019; Chamsi-Pasha & Chamsi-Pasha, 2016). Globally, data suggests reduced postoperative mortality and increased survival for complex CHD, representing a significant global disease burden from birth into adulthood. (Bouma & Mulder, 2017; Lytzen et al., 2019; Zimmerman et al., 2020).

Our understanding of CHD aetiology remains limited, with over 60% remaining unexplained (Botto & Correa, 2003; Yasuhara & Garg, 2021). Chromosomal aneuploidy including Down syndrome account for 10–15%, with monogenic single gene disorders such as DiGeorge syndrome present in up to 25% (Fahed et al., 2013; Kerstjens-Frederikse, 2014; Yasuhara & Garg, 2021). Research in maternal metabolic diseases including diabetes, obesity, and cardiovascular disease have identified metabolic risk factors and potential causative pathways validated in animal models (Botto & Correa, 2003; Chen et al., 2018; Cheng et al., 2012; Hedermann et al., 2021; Helle & Priest, 2020; Suhre et al., 2010; Wang et al., 2011). Non-genetic factors implicated in aetiology include parental smoking, alcohol and drug exposures (Lee et al., 2021). However, evidence strength, mechanistic understanding and causal inference is limited. Maternal folate and folate-containing multivitamin use reduce CHD risk (Cheng et al., 2022; Feng et al., 2015; Goh & Koren, 2008). However, there is not enough evidence to confidently conclude that maternal micronutrient deficiency is associated with fetal CHD (Mires et al., 2022). A greater understanding of potential maternal risk factors and mechanisms could revolutionise primary prevention of CHD.

The human metabolome is a global representation of physiology, representing individual phenotype influenced by genetics and the environment (Hollywood et al., 2006; Monni et al., 2021; Nalbantoglu, 2019). Metabolomics aims to identify and quantify all endogenous and exogenous small molecules and metabolites in a biological system (Letertre et al., 2021; Nalbantoglu, 2019). The metabolome is potentially influenced by several factors including diet, fasting, gender and pregnancy (Handelman et al., 2019; Heinzmann et al., 2012; Kochhar et al., 2006; Krug et al., 2012; Lenz et al., 2004; Monni et al., 2021). However, the variation in studies including sample size, population, biosamples and analytical methods limit the generalisability of findings.

Metabolites measured in an individual represent their metabolic phenotype or metabotype. This reflects the interaction of their genetics and environmental factors (Yousri et al., 2014). Longitudinal studies in individuals have aimed to describe conservation of metabotype over time, with large scale analyses in blood and urine over periods of 3 months to 10 years suggesting conservation of over 70% of metabotype in the majority of participants (Assfalg et al., 2008; Bernini et al., 2009; Carayol et al., 2015; Ghini et al., 2015; Nicholson et al., 2011; Townsend et al., 2013; Yousri et al., 2014). This data suggests that whilst metabolic profiles are under the influence of multiple factors, a large component of individual metabotype is stable over time. Therefore, metabolomic profiles of mothers measured following pregnancy are likely to share significant similarities with those during the periconceptual period. This reproducibility is essential for applications in epidemiological studies of human disease.

In perinatal metabolomics, it is hypothesised that congenital anomalies such as CHD may alter fetal organ function and perfusion, with changes reflected in maternal blood (Monni et al., 2021). Therefore, maternal metabolomic profiling could facilitate biomarker screening in pregnancy, representing a potential fetal effect. Furthermore, metabolic changes could represent maternal aetiological factors for fetal CHD. Studies have assessed metabolomic profiles in mothers of children with CHD during and following the index pregnancy with the identification of several potential metabolites differentiating case and control groups, and development of risk prediction models (Bahado-Singh et al., 2014; Fang et al., 2023; Friedman et al., 2021; Hobbs et al., 2005a, b; Hsu et al., 2022; Taylor et al., 2022; Troisi et al., 2021; Wang et al., 2021; Xie et al., 2019). Whilst this offers promise for further investigation, delineating a fetal effect compared to underlying maternal risk factor profiling remains a priority (Mires et al., 2023).

The aim of this study was to gain insights into potential maternal metabolomic risk factors for childhood CHD. We performed untargeted plasma metabolomic and lipidomic analyses in mothers of children with CHD compared to controls utilising the UK-based children OMACp (Outcome monitoring and risk stratification after cardiac procedure in neonates, infants, children, and young adults born with congenital heart disease; cOMACp) and ALSPAC (Avon Longitudinal Study of Parents and Children) cohorts. We sought to identify metabolites that may provide insight into biological pathways conferring increased risk of CHD in offspring and build accurate risk prediction models.

## Materials and methods

### Study design and cohorts

We performed an observational study assessing untargeted metabolomics and lipidomics in mothers of children with CHD utilising the cOMACp and ALSPAC cohorts.

cOMACp is a multicentre prospective cohort study (Bristol, Leicester, and Dublin) which commenced 01/09/2019. It comprises a data registry and biomaterial bank recruiting children and young adults (0–18) with CHD undergoing cardiac surgery and/or catheterisation alongside their biological mothers. Routine clinical data including diagnosis and co-morbidities alongside maternal questionnaire and medical record data is collected where possible. Women optionally consent to blood sampling at the time of recruitment (Mai et al., 2023). The cohort included 992 children and young adults and 655 mothers when assessed on 31/08/2022.

ALSPAC is a UK-based birth cohort study. In the G0 cohort, pregnant women resident in Avon, UK with pregnancy due dates between April 1991 and December 1992 were invited to take part in the study. 14,541 pregnancies were enrolled, including 14,062 live births with 13,988 children alive at one year of age. Following further attempts to identify eligible children at approximately seven years of age, the total sample size for analyses using any data collected after the age of seven was 15,447 pregnancies, resulting in 15,658 fetuses. Of these 14,901 children were alive at one year of age with this generation termed G1. This comprised 14,833 unique mothers (G0 mothers) enrolled in ALSPAC as of September 2021 (Boyd et al., 2013; Fraser et al., 2013). The ALSPAC-G2 generation (children of G1) began recruitment 6th June 2012 and is ongoing with an aim to continue until all ALSPAC-G1 participants have completed their families. The cohort when assessed in June 2018 had 810 G2 participants, from 548 families, with 83% recruited prior to the age of 3 (Lawlor et al., 2019). Questionnaire, medical record, and clinic data are collected at multiple time points. The ALSPAC study website contains details of all the data that is available through a fully searchable data dictionary and variable search tool (http://www.bristol.ac.uk/alspac/researchers/our-data/ including).

Study data for both cohorts were collected and managed using REDCap (Research Electronic Data Capture) tools hosted at the University of Bristol (UoB; ALSPAC) and University Hospitals Bristol and Weston NHS Foundation Trust (UHBW; cOMACp). REDCap is a secure, web-based software platform designed to support data capture for research studies (Harris et al., 2009).

### Participants

Biological mothers of children with CHD (cases) were sampled from the cOMACp cohort. At the time of the study, 200 mothers of children with structural CHD had EDTA plasma samples available. Of these, nine were excluded due to isolated preterm patent ductus arteriosus and one was excluded due to acquired valvular disease. Therefore, 190 cases were included in this study.

Biological mothers of children without CHD (controls) were sampled from the ALSPAC cohort. At the time of the study, 191 mothers of G2 children had EDTA plasma samples available. Of these, three were excluded due to having a child with known CHD, two were excluded due to having a history of a child with known CHD and 24 were excluded as they were known to be pregnant at the time of sampling. For the remaining 162 subjects, a conjugate measure of likelihood of severe CHD was developed utilising neonatal intensive care admission or hospital/surgical admission data for the child in the first 3 years of life. Of these, 128 had no history of CHD, with 34 having missing data. Given the overall low population incidence of CHD, a decision to utilise the remaining 162 patients as a population control group was made. Of the 162 control samples, eleven mothers gave two or more samples.

### CHD diagnoses and classification

Perioperative diagnosis data and co-morbidity data are routinely collected for cOMACp patients. CHD diagnoses were confirmed through operative/catheter findings and/or echocardiography. Of the 190 cases, 148 had isolated CHD with no known genetic diagnosis, nine had CHD with an extracardiac anomaly but no known genetic diagnosis and 33 had CHD with an associated chromosomal or genetic disorder. For the purposes of analyses in this study, two groups are considered: structural CHD without known genetic diagnosis (*n* = 157; sCHD) and CHD with a known genetic diagnosis (*n* = 33; gCHD).

Patients with CHD often have multiple diagnoses. The International Paediatric and Congenital Cardiac Code (IPCCC) and the Eleventh Revision of the International Classification of Diseases (ICD-11) defines a nomenclature for CHD, utilising 14 subgroups (Jacobs et al., 2021). We utilised this classification to present the spectrum of diagnoses within participants.

The sCHD group was further classified according to severity and clinical classifications for subgroup analysis. EUROCAT (European Commission population-based registry database for congenital anomalies within Europe) defines 16 severe and eight non-severe subgroups of CHD (EUROCAT, 2013). This classification (supplementary Table 1) grouped participants by severe, non-severe or unclassified (EUROCAT, 2013). A participant was designated ‘severe’ if they had any severe diagnosis; ‘non-severe’ if any non-severe diagnosis without a severe diagnosis; and ‘not classified’ if they had neither a severe nor non-severe diagnosis. To assess potential clinical correlations, participants were also classified as having a cyanotic or acyanotic CHD.

### Maternal and child characteristic data

Maternal data including ethnicity, age, body mass index (BMI) and timing of blood sample were collected. Child gender was also recorded. Data sources varied by cohort due to variation, timing, and availability of data. Supplementary Table 2 outlines data sources and variable descriptions by cohort.

### Sample collection

Maternal blood EDTA plasma samples were taken through venepuncture and transferred to the laboratory as soon as possible. cOMACp samples were taken at the time of recruitment. ALSPAC samples were taken at the 36-month postnatal clinic visit. The plasma fraction was aliquoted and stored at -80 degrees. Samples were shipped on dry ice in a single shipment on 26/09/2022 to the Department of Pharmacy, University of Salerno, Fisciano (Italy) for analyses. Package integrity was confirmed on receipt.

### Untargeted metabolomic and lipidomic analyses

Plasma metabolome and lipidome extraction is described in supplementary material section S1. Untargeted analyses were performed on a UHPLC system (Ultimate RS 3000 UHPLC, Thermo Fisher Scientific, Milan, Italy) coupled to TIMS-TOF Pro Quadrupole Time of Flight (Q-TOF, Bruker Daltonics, Bremen, Germany) equipped with an Apollo II electrospray ionization (ESI) probe. Metabolome analysis was performed in both hydrophilic interaction chromatography (HILIC) and reversed phase ultrahigh performance liquid chromatography (RP-UHPLC). Lipidome profiling was performed by RP-UHPLC. Analyses were performed in both positive (+) and negative (-) ionisation mode for both lipidomics and metabolomics. Detailed parameters of LC conditions, MS parameters, metabolite and lipid annotation criteria are fully reported in supplementary material section S1.

### Statistical analysis

Statistical comparison of the case and control group maternal and child characteristics was performed for categorical and continuous variables using Stata v17.0 (StataCorp LLC, Texas, USA). Missing data was present for both cohorts due to incompletion of questionnaires and/or unavailability of clinical records. First, missing data imputation was performed as outlined in the supplementary material section S2 and supplementary Table 3. Categorical variables were compared by chi-squared test. For continuous variables, data were plotted by histogram to visualise normality. Wilcoxon rank sum (Mann-Whitney) test was used to compare continuous variables due to non-normal distributions. For tabulated data where cell counts are < 5, these are represented as < 5 as per ALSPAC reporting guidance.

Lipidomics and metabolomics data analysis approaches are outlined in full in the supplementary material section S2. In this study, the same biological samples were analysed utilising different omics methods resulting in a multi-block dataset: metabolomics ESI^+^, metabolomics ESI^−^, lipidomics ESI^+^ and lipidomics ESI^−^. Data were pre-processed independently for each lipidomics and metabolomics modality, utilising internal standard and total ion sum normalisation respectively. Missing values and zeros were replaced with one-fifth of the minimum value recorded in the dataset for that molecule. Logarithm values were then calculated using a base of 10.

Univariate analysis was performed independently on selected variables without logarithm transformation using Wilcoxon rank sum (Mann-Whitney) test. Prior to further chemometric modelling, data was autoscaled. Multivariate data analysis was conducted on the filtered dataset using custom-developed routines and standard functions in Matlab R2022b (The MathWorks Inc, Natick, MA, USA) (Smilde et al., 2003). Firstly, the unsupervised data reduction tools, Principal Component Analysis (PCA) and SUM-PCA were used in the exploration and visualisation of the data (Smilde & Van Mechelen, 2019; Smilde et al., 2003). To develop chemometric classification models, the Duplex algorithm was used to establish common training and test sets for all omics modalities utilising as input the super scores derived from SUM-PCA (Daszykowski et al., 2002; Snee, 1977). Samples originating from the same patient were assigned to the same training or test set group. Partial Least Squares-Discriminant Analysis (PLS-DA) and sequential and orthogonalized covariance selection (SO-CovSel-LDA) were utilised in the development of chemometric classification models (Biancolillo et al., 2015, 2020; Geladi & Kowalski, 1986 et al., 2013; Roger et al., 2011; Ståhle & Wold, 1987). As sCHD and control groups were approximately balanced, the performance of each model was assessed by specificity, sensitivity, and accuracy. Chemometric approaches are further described in supplementary materials section S2.

Metabolite set enrichment analysis (MSEA) is a method to assess if a list of differentiating metabolites may implicate a biological pathway for further investigation (Xia & Wishart, 2010). We utilised over representation analysis to assess whether metabolites and lipids distinguishing sCHD and control groups identified by chemometric analysis are represented more than expected by chance in SMPDB pathway-based compound lists. The *p*-value represented the probability of seeing at least the number of metabolites from a metabolite set in a compound list by chance (Xia & Wishart, 2010). MSEA was performed utilising MetaboAnalyst 5.0 (https://new.metaboanalyst.ca/ModuleView.xhtml).

### Study access and ethical approvals

Ethical approval for this study was obtained from the ALSPAC Ethics and Law Committee, the Local Research Ethics Committees and cOMACp (REC reference 19/SW/0113; IRAS 261,397) ethical approvals. Utilisation of ALSPAC data and samples obtained ALSPAC executive approval (project number B3982) 07/02/2022. All participants gave informed written consent. Consent for biological samples was collected in accordance with the Human Tissue Act (2004). Research was performed in accordance with the principles of the Declaration of Helsinki.

## Results

### Maternal and child characteristics

Table 1 compares maternal and child characteristics between sCHD and control groups. Ethnicity, child gender and sample timing were similar between cohorts. Of note, due to the methods of cohort recruitment, timing of maternal blood sampling in sCHD had a wider range. Maternal age and BMI potentially differed between sCHD and control groups and were considered potential confounders. Supplementary Table 4 compares gCHD and control group characteristics, demonstrating similar trends.

**Table 1:** Maternal and child characteristic variables following imputation of missing data. Number (proportion) presented for categorical variables and median (IQR) presented for continuous variables. Where a cell count is less than 5, this is represented as < 5. Cell counts < 5 may include 0. *P* value calculated by chi-squared test^*a*^ for categorical variables and Wilcoxon Rank Sum (Mann-Whitney) Test^*b*^ for continuous variables. sCHD – structural congenital heart disease

Maternal Characteristic	sCHD (*n* = 157)	Control (*n* = 162)	*P* Value
Ethnic Group *n* (%)			
White	152 (96.82%)	> 97.00%	0.120 ^a^
Mixed/multiple ethnic groups	< 5	< 5	
Asian or Asian British	< 5	< 5	
Black/African/Caribbean/black British	< 5	< 5	
Child Gender *n* (%)			
Female	70 (44.59%)	74 (45.68%)	0.845 ^a^
Male	87 (55.41%)	88 (54.32%)	
BMI median (IQR)	25.175 (23.14–28.40)	24.1 (21.37–28.70)	0.046 ^b^
Age at Sampling median (IQR)	35 (30–40)	24 (23–26)	< 0.001 ^b^
Sample Timing Median (IQR)	27 (5–97)	37 (36–39)	0.106 ^b^

### CHD diagnoses

**Fig. 1:**
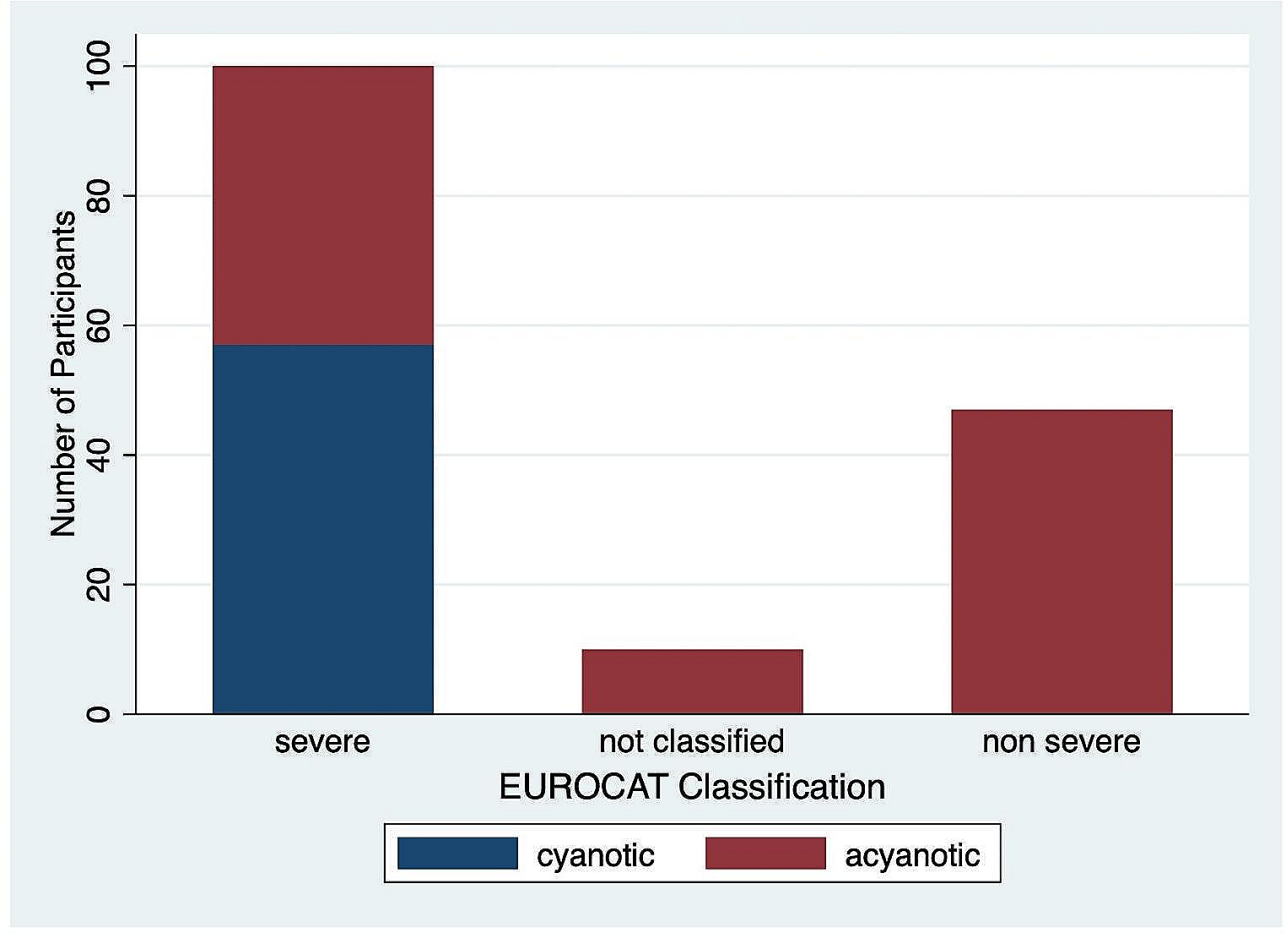
Classification of CHD diagnoses within the sCHD group by EUROCAT severity and clinical classifications. sCHD – structural CHD

Of the 190 sCHD and gCHD participants, 561 CHD diagnoses were recorded (466 sCHD; 95 gCHD). Supplementary Table 5 gives the breakdown of CHD diagnoses present, stratified by the IPCCC/ICD-11 classification (Jacobs et al., 2021). The sCHD group was further classified by severity and clinical diagnosis. 63.7% of sCHD patients had at least 1 EUROCAT-defined severe CHD pathology (EUROCAT, 2013). 36.3% had a clinically defined cyanotic lesion. Figure 1 summarises EUROCAT and clinical classifiers within the sCHD group. This demonstrates the differences in classification methods, with 43.0% of the EUROCAT severe participants having an acyanotic lesion. In the gCHD group, genetic diagnoses included trisomy 21 (*n* = 14), DiGeorge Syndrome (*n* = 5) and other genetic conditions (*n* = 14).

### Metabolomic and lipidomic profiling of sCHD and control mothers

A total of 386 lipids belonging to 17 different subclasses and 113 polar metabolites (amino acids and derivatives, acylcarnitines, fatty acids, nucleotides, organic acids and others) were annotated, with MSI level 2 (supplementary Tables 6 and 7 respectively). Median values obtained for lipids and metabolites respectively were: 943.3 MS/MS score, -0.194 Δppm error, 1.2% ΔCCS and 911.3 MS/MS score, 0.433 Δppm error. 77.5% of the listed lipids achieved an MS/MS score quality higher than 800, with 62.1% having a ΔCCS error lower than 1%. 67.3% of monitored metabolites had a score quality exceeding 800.

**Fig. 2:**
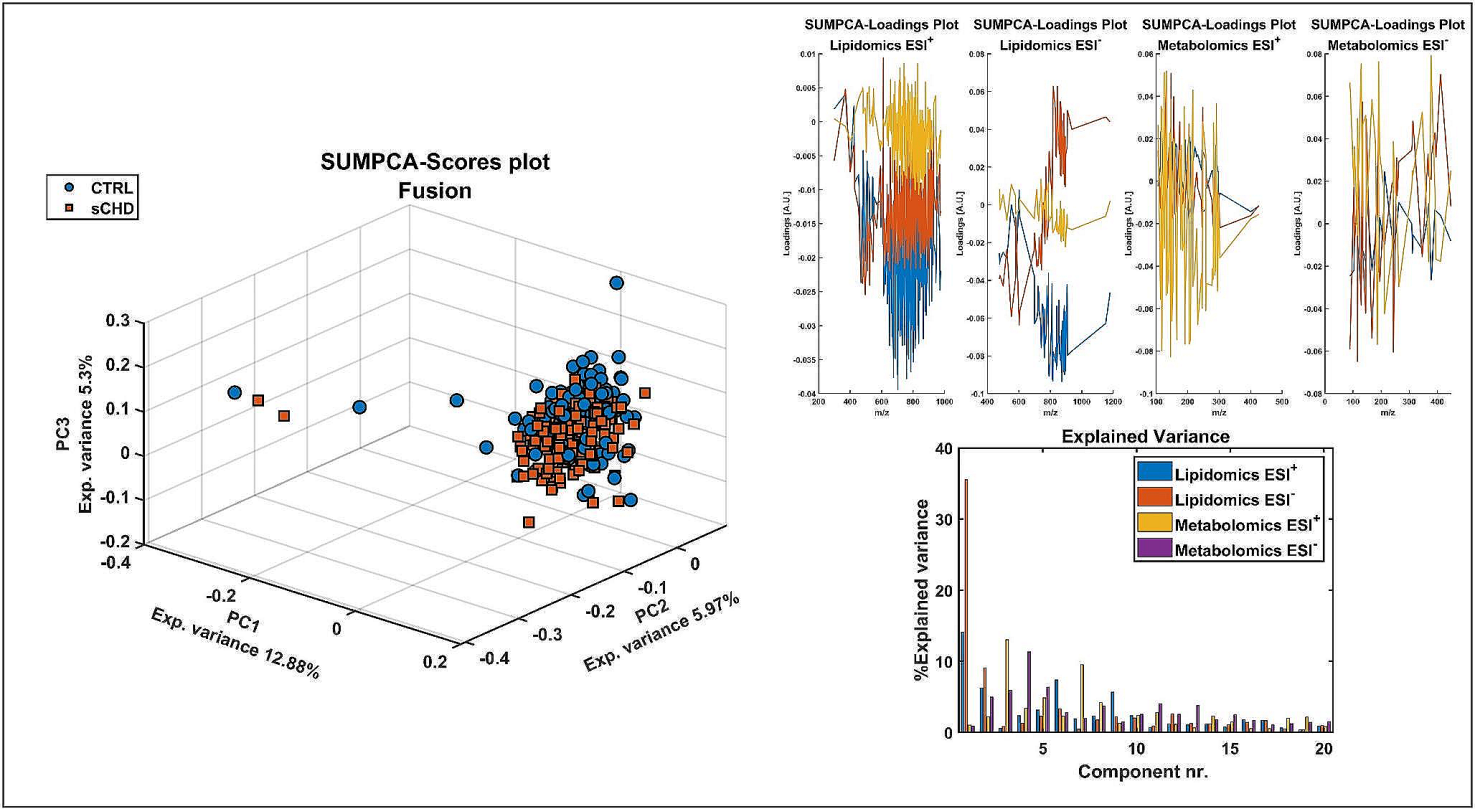
Three-dimensional SUM-PCA illustrating class separation between sCHD (red) and control (blue). SUM-PCA scores, loadings and explained variance plots using low level data fusion of metabolomics and lipidomics analyses. sCHD: structural CHD

Initially, PCA was used to visualise the datasets, firstly, conducted individually on each dataset, including the quality control (QC) samples. The scores plot (not shown) revealed that the pooled QC samples consistently clustered together, suggesting a high level of system stability throughout the experimental batching process. We then assessed the sCHD and control groups. PCA was performed for each block (e.g. different ionisations: metabolomics ESI^+^, metabolomics ESI^−^, lipidomics ESI^+^, lipidomics ESI^−^) shown in supplementary Fig. 1, followed by low level data fusion by SUM-PCA (Fig. 2). There was no clear separation between sCHD and controls on unsupervised analysis, suggesting there is unlikely to be any sampling or experimental bias. This is an important observation given the derivation of case and control samples from different cohorts.

### Accurate classification of sCHD and gCHD mothers by predictive algorithms

Following exploratory analysis, we used PLS-DA for supervised classification of the sCHD and control groups. PLS-DA utilised lipidomics and metabolomics datasets, followed by low-level data fusion. Table 2 shows model performance for each dataset. Modelling with the metabolomics dataset alone performed best on cross validation, with an sCHD test set mean accuracy of 91.58% (sCHD class sensitivity 92.68%; specificity 90.74%). This model included 35 metabolites with VIP scores > 1. Supplementary Fig. 2 shows the 20 metabolites with the highest VIP scores from this model. The metabolomics ESI^+^ model also performed well (sCHD test set mean accuracy 92.63%; sensitivity 92.68%; specificity 92.59%). All metabolites with VIP scores > 1 in the metabolomics ESI^+^ model were common to the metabolomics model. Whilst the classification accuracy through PLS-DA is high, clinical utility remains limited, given the large number of analytes contributing to model performance. Establishing individual metabolites with clinical significance would aid in transition towards targeted assay development.

**Table 2:** PLS-DA modelling of sCHD and control groups. Data analysed as lipidomics and metabolomics in electron spray ionisation (ESI) positive (+) and negative (-) modes independently, followed by low level data fusion. Table A: Training and Cross-Validation (CV) correct classification mean accuracy, sensitivity and specificity shown as %. VIP shows number of analytes with VIP score > 1 within the model. Table B: sCHD test group correct classification mean accuracy, sensitivity and specificity shown as %. VIP: variable important in projection; sCHD: structural CHD

	Mode	No. Analytes	VIP	No. Latent Variables	Accuracy (%)		Sensitivity (%)		Specificity (%)	
					Training	CV	sCHD Training	sCHD CV	sCHD Training	sCHD CV
Table A	Lipidomics ESI^+^	307	101	6	95.54	76.63	95.87	78.51	95.15	74.76
	Lipidomics ESI^−^	79	21	4	79.46	74.62	83.47	79.34	74.76	69.90
	Metabolomics ESI^+^	74	19	15	98.66	93.22	98.35	94.21	99.03	92.23
	Metabolomics ESI^−^	39	12	5	91.96	88.80	94.21	89.26	89.32	88.35
	Lipidomics	386	122	3	86.16	78.02	87.60	79.34	84.47	76.70
	Metabolomics	113	35	9	99.55	95.51	99.17	95.87	100.00	95.15
	Fusion	499	130	7	100.00	92.25	100.00	94.21	100.00	90.29
	Mode				Test Accuracy (%)		sCHD Test Sensitivity (%)		sCHD Test Specificity (%)	
Table B	Lipidomics ESI^+^				73.68		95.12		57.41	
	Lipidomics ESI^−^				67.37		87.80		51.85	
	Metabolomics ESI^+^				92.63		92.68		92.59	
	Metabolomics ESI^−^				87.37		95.12		81.48	
	Lipidomics				72.63		90.24		59.26	
	Metabolomics				91.58		92.68		90.74	
	Fusion				85.26		87.80		83.33	

**Fig. 3:**
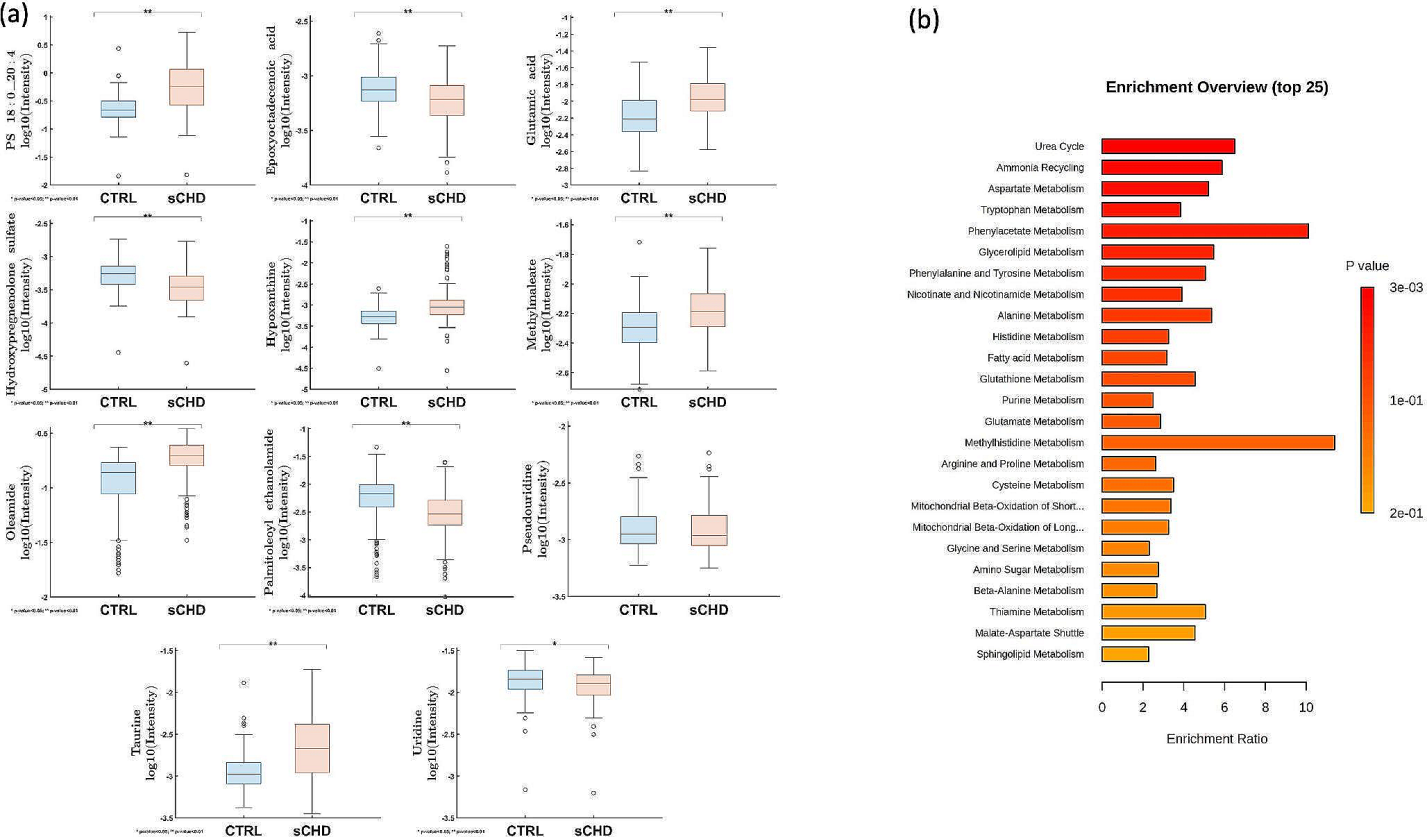
(**a**) Summary of metabolites and lipids identified through SO-CovSel-LDA models classifying sCHD and control groups. Boxplots of normalised intensities for sCHD and control groups. *P* values calculated on univariate analysis through Mann Whitney U Test. (**b**) Summary of enriched analytes from metabolite set enrichment analysis (MSEA) of selected analytes (*n* = 37) derived from SO-CovSel-LDA models. Figures generated from MetaboAnalyst 5.0 (https://new.metaboanalyst.ca/ModuleView.xhtml) showing enrichment ratio and *P*-value (hypergeometric test). sCHD: structural CHD

Therefore, we utilised SO-CovSel-LDA as a multi-block classification method to select the minimum set of non-redundant variables, providing a reliable predictive model (Biancolillo et al., 2020; Smilde et al., 2003). SO-CovSel-LDA selects, during model development, the minimum set of non-redundant variables from different data blocks, providing direct information about explanatory variables. Variables are selected with the greatest covariance to the outcome, whilst eliminating redundant information across blocks. 64 SO-CovSel-LDA models were constructed using all possible combinations of the four blocks for sCHD and controls (supplementary Table 8). After considering variable number and potential overfitting, Table 3 summarises the selected most accurate models on cross validation with the smallest number of variables. The best performing models have an sCHD test set mean accuracy of 94.74% (sCHD class sensitivity 93.33%; specificity 96.00%) utilising only 11 analytes (Table 3B). Supplementary Table 9 summarises the 11 selected analytes from SO-CovSel-LDA models, along with mean normalised intensities, direction of change and *p* values between sCHD and control groups. Trends are reported in Fig. 3A as box plots. These are taurine (HMDB0000251), oleamide (HMDB0002117), palmitoleoyl Ethanolamide (HMDB0013648), epoxyoctadecenoic acid (HMDB0004701), glutamic Acid (HMDB0000148), hydroxypregnenolone Sulfate (HMDB0000416), hypoxanthine (HMDB0000157), methylmaleate (HMDB0000634), pseudouridine (HMDB0000767), uridine (HMDB0000296) and PS 18:0_20:4 (HMDB0012383).The order of the blocks does not impact performance with comparable variables chosen in each combination, demonstrating model stability. Of interest, nine of the 10 metabolites highlighted through SO-CovSel-LDA have VIP scores > 1 in the metabolomics PLS-DA model, showing alignment across different modelling approaches.

**Table 3:** Best performing SO-CovSel-LDA models utilising the four blocks (metabolomics and lipidomics in electron spray ionisation (ESI) positive (+) and negative (-) modes) for sCHD and control groups. Mode shows order of blocks in model. Correct classification mean accuracy, sensitivity and specificity shown as %. Table A: Model performance in training and cross-validation (CV) for sCHD and control. Table B: Model performance in sCHD and control test set. Table C: Model performance for gCHD test set. Met: metabolomics; Lip: lipidomics; sCHD: structural CHD; gCHD: genetic CHD

	Mode	No. Analytes				Accuracy (%)		Sensitivity (%)		Specificity (%)	
		Block 1	Block 2	Block 3	Block 4	Training	CV	sCHD Training	sCHD CV	sCHD Training	sCHD CV
Table A	Met (ESI^+^) Lip (ESI^−^) Met (ESI^−^)	4	1	6	/	97.77	97.32	95.54	94.64	100.00	100.00
	Lip (ESI^+^) Met (ESI^+^) Lip (ESI^−^) Met (ESI^−^)	0	4	1	6	97.77	97.32	95.54	94.64	100.00	100.00
	Met (ESI^+^) Lip (ESI^+^) Lip (ESI^−^) Met (ESI^−^)	4	0	1	6	97.77	97.32	95.54	94.64	100.00	100.00
	Met (ESI^+^) Lip (ESI^−^) Lip (ESI^+^) Met (ESI^−^)	4	1	0	6	97.77	97.32	95.54	94.64	100.00	100.00
	Met (ESI^+^) Lip (ESI^−^) Met (ESI^−^) Lip (ESI^+^)	4	1	6	0	97.77	97.32	95.54	94.64	100.00	100.00
	Mode					Test Accuracy (%)		sCHD Test Sensitivity (%)		sCHD Test Specificity (%)	
Table B	Met (ESI^+^) Lip (ESI^−)^ Met (ESI^−^)					94.74		93.33		96.00	
	Lip (ESI^+^) Met (ESI^+^) Lip (ESI^−^) Met (ESI^−^)					94.74		93.33		96.00	
	Met (ESI^+^) Lip (ESI^+^) Lip (ESI^−^) Met (ESI^−^)					94.74		93.33		96.00	
	Met (ESI^+^) Lip (ESI^−^) Lip (ESI^+^) Met (ESI^−^)					94.74		93.33		96.00	
	Met (ESI^+^) Lip (ESI^−^) Met (ESI^−^) Lip (ESI^+^)					94.74		93.33		96.00	
	Mode					gCHD Test Accuracy (%)		gCHD Test Sensitivity (%)		gCHD Test Specificity (%)	
Table C	Met (ESI^+^) Lip (ESI^−^) Met (ESI^−^)					96.97		96.97		96.97	
	Lip (ESI^+^) Met (ESI^+^) Lip (ESI^−^) Met (ESI^−^)					96.97		96.97		96.97	
	Met (ESI^+^) Lip (ESI^+^) Lip (ESI^−^) Met (ESI^−^)					96.97		96.97		96.97	
	Met (ESI^+^) Lip (ESI^−^) Lip (ESI^+^) Met (ESI^−^)					96.97		96.97		96.97	
	Met (ESI^+^) Lip (ESI^−^) Met (ESI^−^) Lip (ESI^+^)					96.97		96.97		96.97	

Given the smaller sample size of gCHD, models were not independently built for this dataset, but gCHD was utilised as a separate test set. Similar test accuracies for both the sCHD and gCHD groups are seen across various models (Table 3C). To assess the potential influence of maternal age and BMI, the normalised intensity for each analyte was plotted against maternal age and BMI for each sample. Correlation coefficients suggested low correlation of maternal BMI and age to each analyte (supplementary Fig. 3 and supplementary Table 10).

We combined the analytes identified through the selected SO-CovSel-LDA models and metabolomics PLS-DA model (36 polar metabolites, one lipid) for MSEA to assess potential biological pathways. The urea cycle (p0.003; expected 0.623; hits 4), ammonia recycling (p0.004; expected 0.68; hits 4) and aspartate metabolism (p0.005; expected 0.752; hits 4) were the most implicated pathways (Fig. 3B).

### Utilising metabolomic and lipidomic profiles to stratify CHD severity and clinical classification

Following assessment of the sCHD and control groups, we sought to ascertain if metabolomic and lipidomic differences could accurately distinguish mothers of children with different classifications of CHD. PLS-DA used the lipidomics and metabolomics datasets, followed by low-level data fusion within the sCHD group to assess classification by the EUROCAT and clinical classifications. Supplementary Table 11 summarises the most accurate models (metabolomic ESI^+^) in cross validation for the EUROCAT and clinical classifications. Test set mean accuracy was lower for EUROCAT classification (78.72%). Whilst overall correct classification was greater for clinical classifications (mean accuracy 89.36%), this was associated with lower sensitivity for cyanotic and specificity for acyanotic CHD in test performance.

## Discussion

### Main findings

Using comprehensive untargeted metabolomic and lipidomic analysis, we have identified a potential signature in maternal plasma distinguishing mothers of children with CHD compared to controls following pregnancy. We employed multi-block modelling methods to accurately classify cases, with analytes spanning amino acid, lipid, and nucleotide/nucleoside analogue classes particularly important.

Several studies have assessed blood and urine metabolomic profiles in mothers of children with CHD both within and following the index pregnancy (Bahado-Singh et al., 2014; Fang et al., 2023; Friedman et al., 2021; Hobbs et al., 2005a, b; Hsu et al., 2022; Taylor et al., 2022; Troisi et al., 2021; Wang et al., 2021; Xie et al., 2019). Results show promise for identifying potentially discriminating metabolites. However, challenges remain in distinguishing potential fetal effects on the maternal metabolome from analyses within pregnancy and elucidating underlying maternal risk factor profiles (Mires et al., 2023). Our findings offer potential routes to understanding maternal metabolic risk factor profiles for fetal CHD.

#### Amino acids and derivatives

Glutamate is an amino acid involved in amino acid derivation, energy production and cellular protection (Walker & van der Donk, 2016). Higher glutamate is associated with adverse cardiovascular parameters, mediated through endothelial cell oxidative stress and osmotic damage (Durante, 2019; Hinshaw & Burger, 1990; Parolari et al., 1997). Glutamate is important in glutathione formation, essential in redox homeostasis and cellular protection from oxidative damage (Walker & van der Donk, 2016). In oxidative stress, glutathione is oxidised to GSSG, shown to be increased in mothers of children with CHD following pregnancy (Hobbs et al., 2005b; Wu et al., 2004). Alterations in glutamate concentrations have further been seen in the blood of children with CHD (O’Connell et al., 2021; Yu et al., 2018; Yuan et al., 2020). During pregnancy, amino acids are necessary for fetal growth and development (Wu et al., 2015). Glutamate concentrations are higher in the fetus than the mother with lower placental uptake seen in growth restricted pregnancies (Camelo et al., 2004; McIntyre et al., 2020). As such, alterations in maternal glutamate concentrations could influence fetal development.

Taurine is an amino acid with roles in membrane stabilisation, membrane phospholipid metabolism regulation and cardiomyocyte osmoregulation (Hamaguchi et al., 1991; Lambert et al., 2015; Schaffer et al., 2010). Mothers of children with CHD had increased taurine compared to controls in this study. Animal models show taurine is essential to the developing fetus with mice knocked out for the taurine transporter showing defects in multiple systems including markers of cardiomyopathy and heart failure (Ito et al., 2008). Excessive taurine in rats led to accelerated growth, obesity and insulin resistance (Hultman et al., 2007). Taurine has been shown to be increased in the blood of children with CHD compared to controls (Yu et al., 2018; Yuan et al., 2020). Maternal metabolomic assessment during pregnancy showed decreased taurine in mothers of children with CHD compared to controls, however, this was limited by a small sample size (*n* = 17 cases) (Fang et al., 2023). The human fetus and placenta lack the enzymes necessary for taurine synthesis, with taurine transport from maternal plasma to the umbilical circulation (Lambert et al., 2015). Therefore, changes in the maternal circulation are unlikely to be fetally derived.

#### Lipids, lipid-like molecules, and lipid messengers

Phosphatidylserine is a phospholipid involved in cell and mitochondrial membrane structure and function, derivation of phosphatidylethanolamine and formation and stability of lipoproteins for lipid transport and lipogenesis (van der Veen et al., 2017; Vance, 2018). Several studies have identified differences in maternal phospholipid profiles between mothers of children with CHD and controls during and following pregnancy, with similar findings in children and adults with CHD (Bahado-Singh et al., 2014; Guvenc et al., 2023; Hsu et al., 2022; Michel et al., 2020; Taylor et al., 2022).

Palmitoleoyl ethanolamide (PEA) is a fatty amide part of the N-acetylethanolamine (NAE) lipid messenger family, generated from phospholipid metabolism (Mock et al., 2023). Mothers of children with CHD had reduced PEA compared to controls in this study. NAEs have known anti-inflammatory effects. Administration of PEA in rat myocardial ischaemia reduces markers of inflammation and apoptosis in reperfusion, showing potential myocardial protective effects (Di Paola et al., 2016).

Oleamide is an endogenous lipid mediator discovered within the central nervous system in sleep deprivation (Hiley & Hoi, 2007). Epoxyoctadecenoic acid is a medium-chain fatty acid, produced as a perixodation product of linoleic acid from low density lipoprotein. It has been shown to accumulate in cardiovascular disease processes such as atherosclerosis (Jira & Spiteller, 1999). Administration in animal models may lead to heart failure and cardiovascular death (Fukushima et al., 1988). Methylmaleate is a methyl-fatty acid with immunomodulatory and antioxidant roles (Chen et al., 2022). There is little current evidence on the roles of these metabolites in the embryology or structure of the heart.

#### Nucleoside and nucleotide analogues

Hypoxanthine is a purine derivative, formed in the breakdown of adenosine triphosphate (ATP). Increased levels have been identified in tissue and plasma during hypoxic events including myocardial infarction (Farthing et al., 2015; Saugstad, 1988). Hypoxanthine can cross the placenta (Barros, 1994). Previous studies in and following pregnancy have suggested increased hypoxanthine in mothers of children without CHD (Fang et al., 2023; Wang et al., 2021). Hypoxanthine was increased in mothers of children with CHD within this study.

Uridine is a pyrimidine nucleotide for RNA, glycogen synthesis and lipid deposition (Zhang et al., 2020). Plasma metabolomic analysis of mothers of children with CHD following pregnancy has previously suggested increased uridine compared to controls (Wang et al., 2021). Lower uridine in cases was seen in this study. Pseudouridine, an isomer of uridine, a component and regulatory controller of RNA was also lower in cases (Charette & Gray, 2000).

#### Metabolomic and lipidomic profiles stratified by diagnosis

In previous studies considering maternal metabolic profiles in mothers of children with CHD, inclusion criteria are generally limited to isolated CHD without a known underlying genetic syndrome. In this study, the gCHD group allowed for assessment in a presumed genetic or chromosomal aetiology. Interestingly, model performance was similar in the sCHD and gCHD subgroups, suggesting potential commonalities between maternal risk profiles in the presence of genetic conditions. Further investigation with a larger sample size of specific genetic conditions would be beneficial.

We developed models to assess whether mothers of children with different classifications of CHD could be accurately classified on metabolomic and lipidomic markers. Models performed less accurately for the EUROCAT classification, potentially related to its lack of biological basis. Model performance was better for clinical classifications based on cyanosis; however, sensitivity and specificity were not optimal. Further investigation is warranted as greater understanding could aid in understanding maternal risk or prognostic markers, as well as potentially improving clinical classification systems of CHD.

### Strengths and limitations

This study performs comprehensive untargeted and lipidomic analysis in a well characterized cohort of CHD patients. Here, we utilise maternal sampling following pregnancy to infer risk factor profiles for fetal CHD. Whilst studies suggest a large component of an individual’s metabotype is stable over time, samples taken may not be reflective of the periconceptual state (Assfalg et al., 2008; Bernini et al., 2009; Carayol et al., 2015; Ghini et al., 2015; Nicholson et al., 2011; Townsend et al., 2013; Yousri et al., 2014). However, prospective pre-conception sampling is infeasible in this setting. Several metabolites potentially implicated as maternal risk factors have been identified, but the observational nature of the study limits the ability to currently establish causative links.

Samples were taken following pregnancy. Participants known to be pregnant were excluded, however, pregnancy status was not routinely recorded for cOMACp participants and some ALSPAC data was missing. Efforts were made to identify potential differences between case and control groups; however, there remains the potential for unmeasured confounders. It was not possible to accurately compare further maternal characteristics between cohorts. Reassuringly, we did not observe any separation or subclassifications in classes within unsupervised and supervised models; suggesting potential known and unknown confounders are unlikely to have had a substantial effect. Future work will further explore the interactions of maternal characteristics with important metabolites identified as well as considering potential additional unexplored confounders.

## Conclusions

Here, untargeted plasma metabolomic and lipidomic analysis has facilitated the development of sensitive risk prediction models identifying mothers of children with CHD. Implicated metabolites and lipids offer promise for maternal risk factor profiling, and greater understanding of biological mechanisms of CHD pathogenesis. Validation of findings in greater sample sizes, with development of targeted platforms will aid greater understanding going forward.

### Electronic supplementary material

Below is the link to the electronic supplementary material.

Supplementary Material 1

Supplementary Material 2

Supplementary Material 3

Supplementary Material 4

Supplementary Material 5

## Data Availability

Data is provided within the manuscript or supplementary information files.
