# Supplementary figures and images for: Plasma metabolomic and lipidomic profiles accurately classify mothers of children with congenital heart disease: an observational study
Source: Metabolomics. 2024 Jul 2;20(4):70. doi: 10.1007/s11306-024-02129-8 (PMC11219374; doi:10.1007/s11306-024-02129-8)

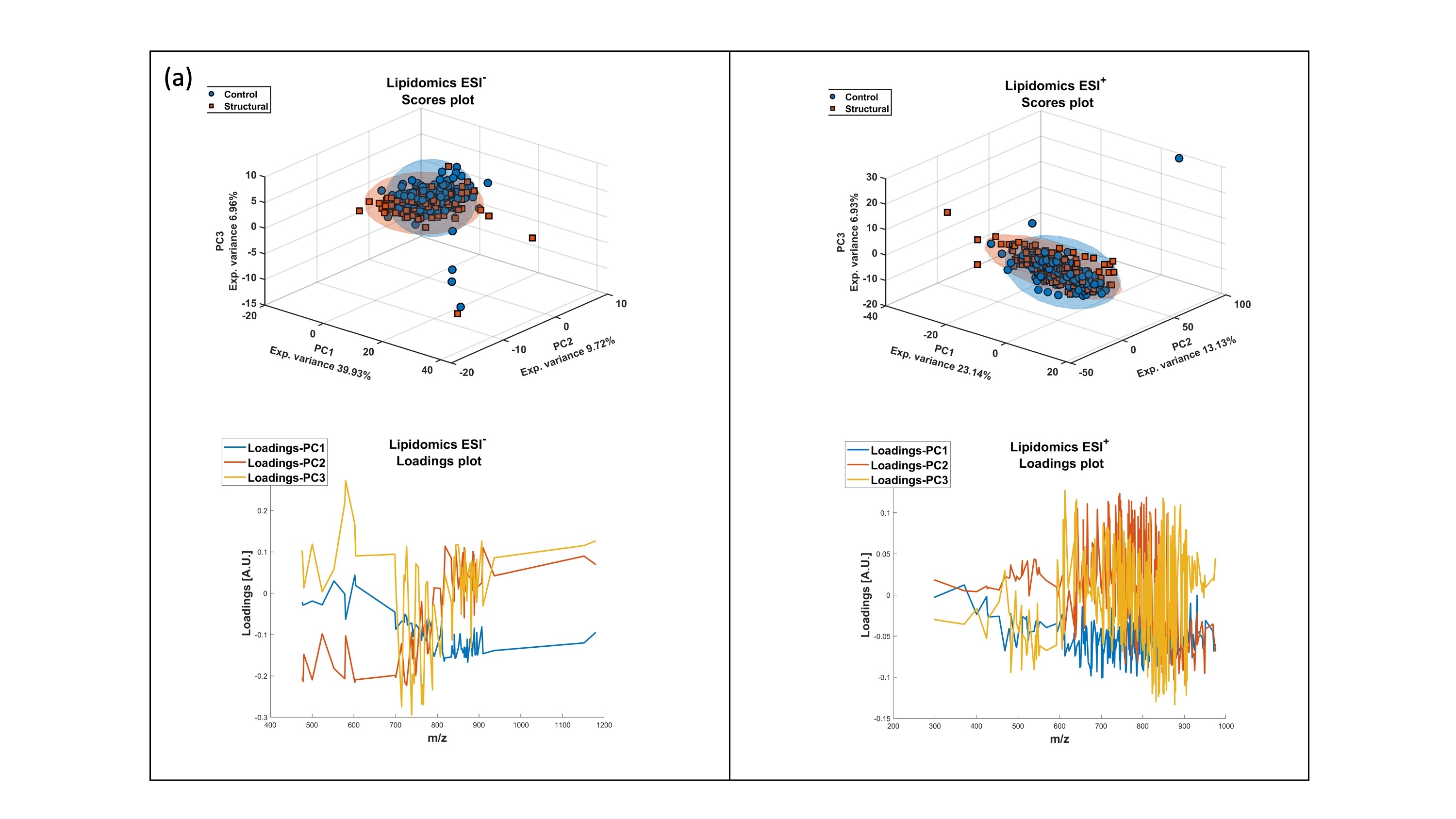

Supplement: Supplementary file 1 — Supplementary Material 1 [file 11306_2024_2129_MOESM1_ESM.jpg]

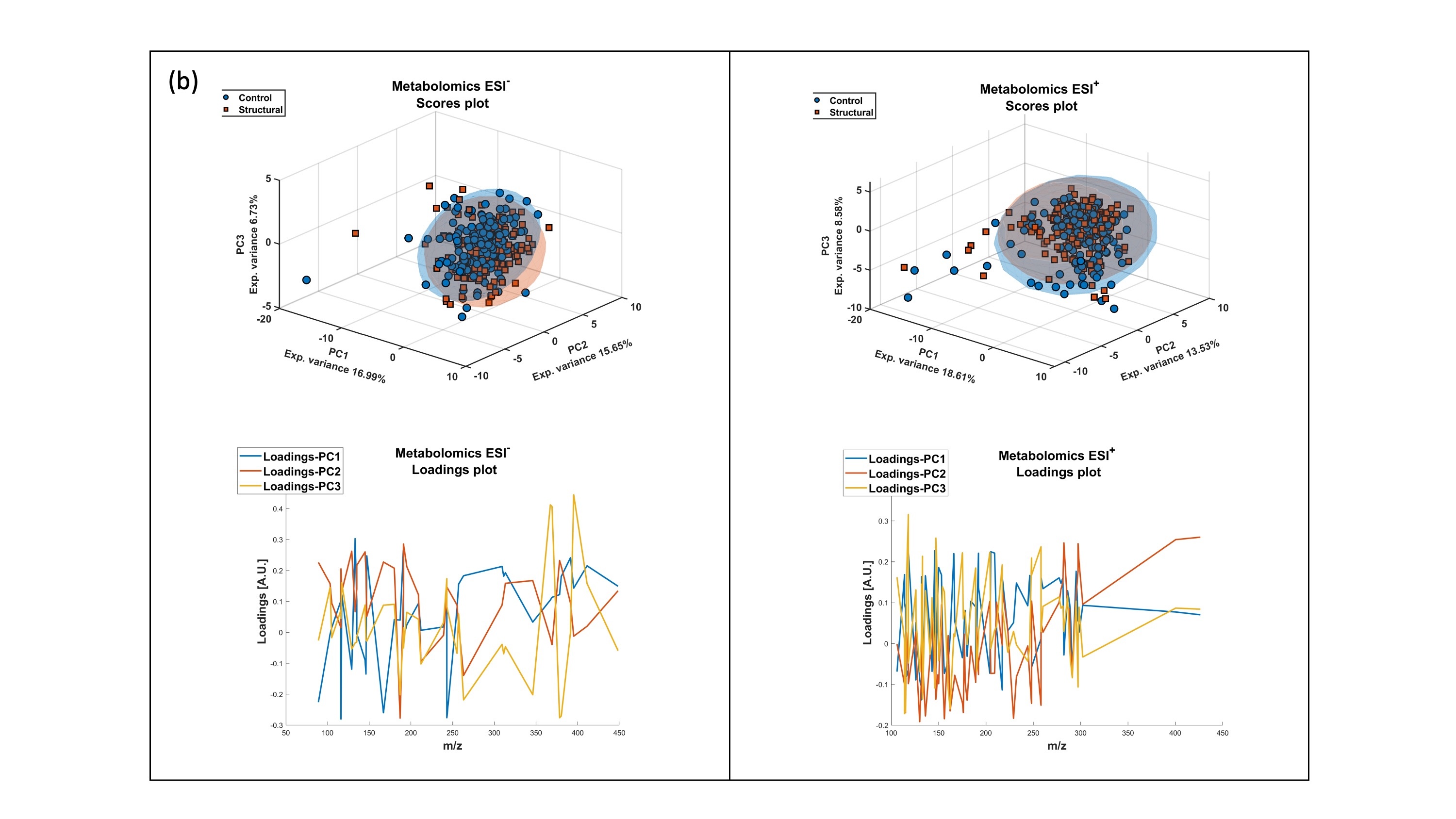

Supplement: Supplementary file 2 — Supplementary Material 2 [file 11306_2024_2129_MOESM2_ESM.jpg]

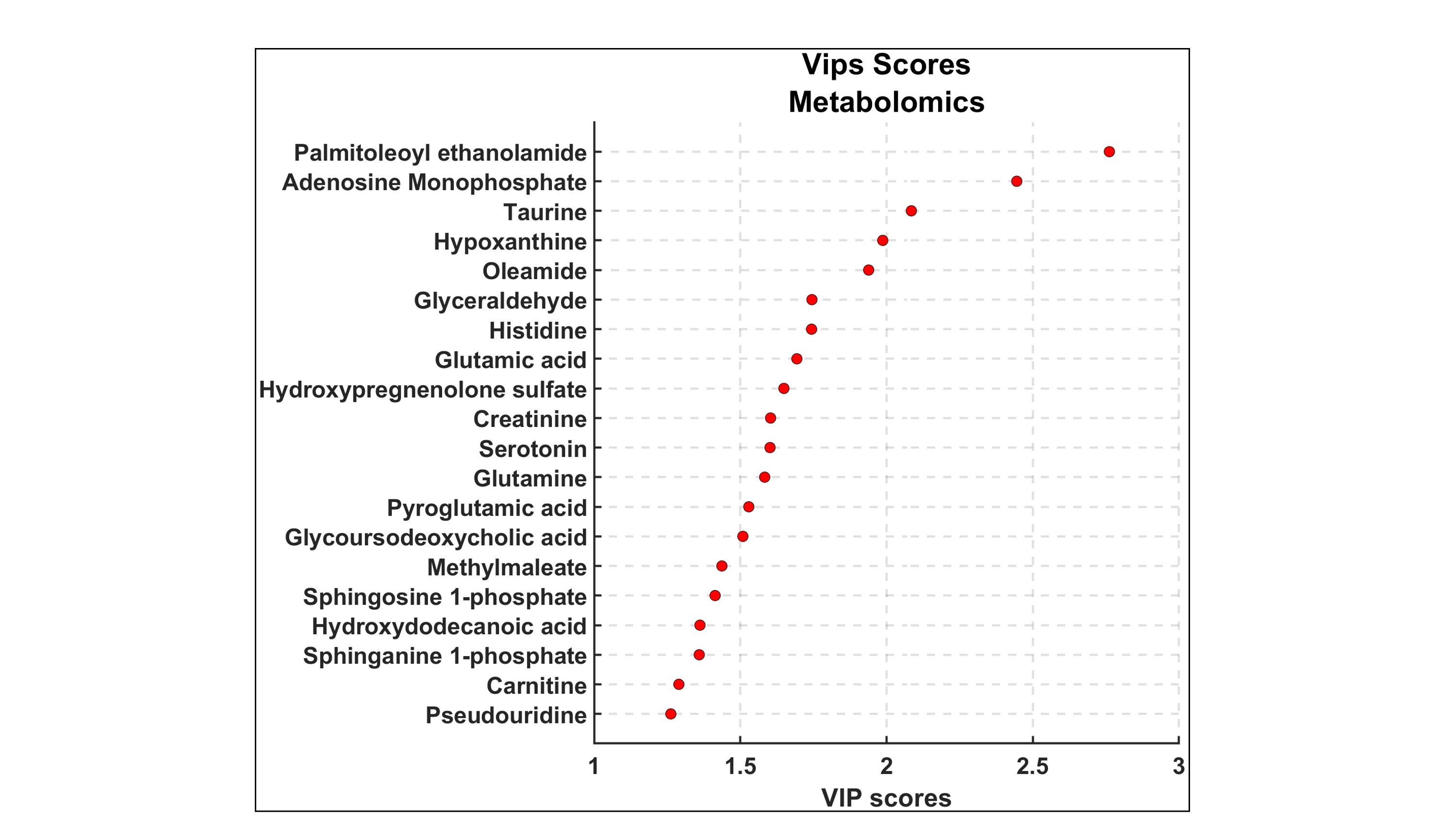

Supplement: Supplementary file 3 — Supplementary Material 3 [file 11306_2024_2129_MOESM3_ESM.jpg]

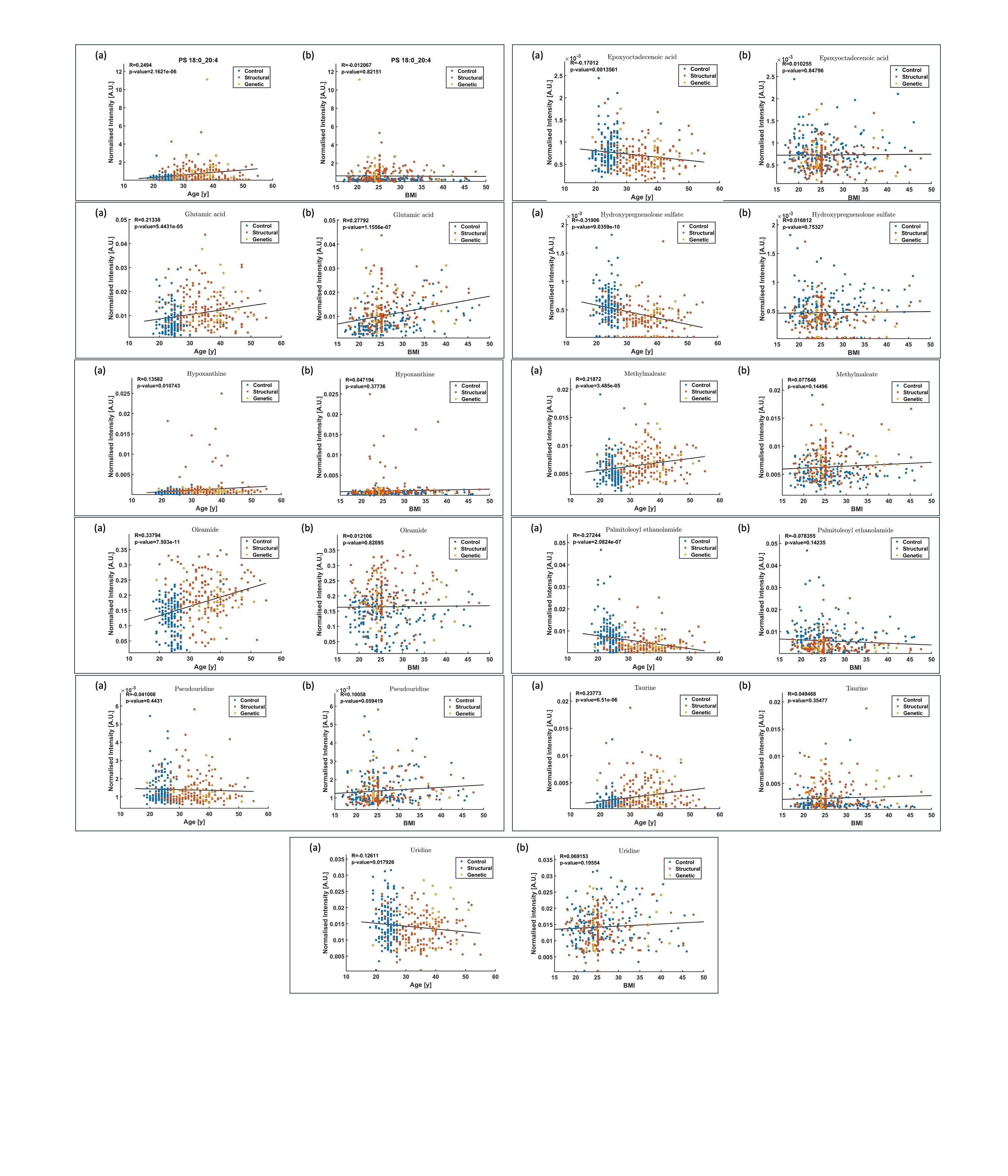

Supplement: Supplementary file 4 — Supplementary Material 4 [file 11306_2024_2129_MOESM4_ESM.jpg]
