# Supplementary material for: Plasma metabolomic and lipidomic profiles accurately classify mothers of children with congenital heart disease: an observational study
Source: Metabolomics. 2024 Jul 2;20(4):70. doi: 10.1007/s11306-024-02129-8 (PMC11219374; doi:10.1007/s11306-024-02129-8)
Supplement: Supplementary file 5 — Supplementary Material 5 [file 11306_2024_2129_MOESM5_ESM.docx]

**Supplementary material for the manuscript:**

**Plasma metabolomic and lipidomic profiles accurately classify mothers of children with congenital heart disease: a case control study.**

Stuart Mires^1,2^, Eduardo Sommella^3^, Fabrizio Merciai^3^, Emanuela Salviati^3^, Vicky Caponigro^3^, Manuela Giovanna Basilicata^3^, Federico Marini^4^, Pietro Campiglia^3^, Mai Baquedano^1^, Tim Dong^1^, Clare Skerritt^2^, Kelly-Ann Eastwood^1,2^, Massimo Caputo^1,2^.

^1^Translational Health Sciences, University of Bristol, Bristol, UK.

^2^University Hospitals Bristol and Weston NHS Foundation Trust, Bristol, UK.

^3^Department of Pharmacy, University of Salerno, Salerno, Italy.

^4^Department of Chemistry, University of Rome, Rome, Italy.

**Supplementary Methods**

**Section S1**

**Chemicals**

LC–MS-grade Water (H_2_O), acetonitrile (ACN), methanol (MeOH), isopropanol (IPA), 1-butanol (BuOH), methyl tert-butyl ether (MTBE), and additives formic acid (HCOOH), acetic acid (CH_3_COOH), ammonium formate (HCOONH_4_) and ammonium acetate (CH_3_COONH_4_) were purchased from VWR (Milan, Italy). Deuterated (SPLASH^®^) and authentic lipid standards (LightSPLASH^®^) were purchased from Avanti Polar Lipids (Alabaster, AL, U.S.A). Unless otherwise stated other reagents were purchased from Merck.]

**Plasma lipidome extraction**

Before extraction, samples from cOMACp and ALSPAC were randomized. Lipids were extracted as follows. Briefly, 20 µL of plasma were thawed on ice and added to 225 µL of ice cold MeOH containing a mix of deuterated standards and vortexed for 10 s. Subsequently, 750 µL of cold MTBE were transferred to the tube and the solution was continuously agitated in a thermomixer (Eppendorf, Milan, Italy) for 10 min, 300 rpm at 4°C. Then, 188 µL of H_2_O was added and samples were shacked for 20 s and then centrifuged at 14680 rpm for 10 min at 4°C to induce phase separation. The upper layer was collected and evaporated using a SpeedVac (Savant, Thermo Scientific, Milan, Italy). The dried samples were dissolved in 100 µL of BuOH/IPA/H_2_O 8/23/69 (*v/v %*) before the UHPLC-TIMS-MS analysis. A quality control (QC) sample was prepared by pooling the same aliquot (5 µL) from each sample and extracted as above.

**Plasma metabolome extraction**

Before extraction, samples from cOMACp and ALSPAC were randomized. 300 µL of ice cold MeOH 80% (*v/v %*) were added to 30 µL of plasma, thawed on ice. Samples were shacked for 12 minutes at 15 Hz, incubated at -20° for 30 minutes and, subsequently, centrifuged for 10 minutes at 14680 rpm. Supernatants were collected, dried using a SpeedVac (Savant, Thermo Scientific, Milan, Italy) and stored at -20° C before LC-MS/MS analysis. QC samples were extracted as previously described.

**Untargeted lipidomics**

UHPLC-TIMS-MS analyses were performed on an Ultimate RS 3000 UHPLC (Thermo Fisher Scientific, Milan, Italy), which comprised a RS 3000 autosampler, column oven and binary pump with a 35 µL mixer. All connections were Viper (Thermo Fisher Scientific) stainless steel capillaries (0.100 mm I.D.). The column outlet was connected to the MS source with a peek tubing (0.100 mm I.D.) of the shortest length possible. The UHPLC system was coupled online to a TimsTOF Pro Quadrupole Time of Flight (Q-TOF) (Bruker Daltonics, Bremen, Germany) equipped with an Apollo II electrospray ionization (ESI) probe. The separation was performed with an Acquity UPLC CSH^TM^ C18 column (50 × 2.1 mm; 1.7 μm, 130 Å) protected with a VanGuard CSH^TM^ precolumn (5.0 × 2.1 mm; 1.7 μm, 130 Å) (Waters, Milford, MA, U.S.A). The column temperature was set at 65°C, a flow rate of 0.55 mL/min was used, mobile phase consisted of (A): ACN/H_2_O 60:40 (*v/v* *%*) and (B): IPA/ACN 90:10 (*v/v %*) both buffered with 10 mM HCOONH_4_ and 0.1% HCOOH for positive ionization while 10 mM CH_3_COONH_4_ and 0.1% CH_3_HCOOH additives were used for negative ionization mode. The following gradient has been used: 0 min, 40% B; 0.4 min, 43% B; 0.425 min, 50% B; 0.9 min, 57% B; 2.0 min, 70% B; 2.950 min, 99% B; 3.3 min, 99% B; 3.301, 40% B and then 0.7 min for column re-equilibration. The TIMS-MS analyses were performed in data-dependent parallel accumulation serial fragmentation (DDA-PASEF) with both positive and negative ionization, in separate runs. For the assessment of repeatability and instrument stability over time, a quality control strategy was applied. Samples were injected in randomized order and blank samples were injected regularly and used to assess carryover and exclude background signals. The injection volume was set at 2 µL. Source parameters: Nebulizer gas (N_2_) pressure: 3.0 Bar, Dry gas (N_2_): 10 L/min, Dry temperature: 250°C. Mass spectra were recorded in the range m/z 50–1500, with an accumulation and ramp time to 100 ms each. The ion mobility was scanned from 0.55 to 1.80 Vs/cm^2^. Precursors for data-dependent acquisition were isolated within ±2 m/z and fragmented with an ion mobility-dependent collision energy ranging from 20 to 40 eV in positive mode and with a fix energy collision (40 eV) in negative mode. The total acquisition cycle was of 0.32 s and comprised one full TIMS-MS scan and two PASEF MS/MS scans. Exclusion time was set to 0.1 min, Ion charge control (ICC) was set to 7.5 Mio. To enhance MS/MS spectra quality, we additionally performed several separate experiments with different collision energy mode (TIMS-STEPPING ion mobility-dependent collision energy mode: CE [eV] #1: 20-40 and CE [eV] #2: 35-50). The instrument was calibrated for both mass and mobility using the ESI-L Low Concentration Tuning Mix with the following composition: [m/z, 1/K_0_: (322.0481, 0.7318 Vs cm^−2^), (622.0290, 0.9848 Vs cm^−2^), (922.0098, 1.1895 Vs cm^−2^), (1221,9906, 1.3820 Vs cm^−2^)] in positive mode and [m/z, 1/K_0_: (301.99814, 0.6678 Vs cm^−2^), (601.97897, 0.8781 Vs cm^−2^), (1033.98811, 1.2525 Vs cm^−2^), (1333.96894, 1.4015 Vs cm^−2^)] in negative mode.

**Untargeted metabolomics**

The analysis of polar metabolome was performed on the same MS platform. For the assessment of repeatability and instrument stability over time, a quality control (QC) strategy was applied. DDA-MS analyses were performed in positive and negative ionization, in separate runs and with different chromatographic approaches. Samples were injected in randomized order and blank samples were injected regularly and used to assess carryover and exclude background signals. In detail, ESI^+^ analysis were performed with an SeQuant^®^ ZIC^®^-HILIC column (100 × 2.1 mm; 3.5 µm, 100Å) protected with a SeQuant^®^ ZIC^®^-HILIC Guard precolumn (20 × 2.1 mm) (Supelco^®^). The column temperature was set to 40°C, and the flow rate was 0.350 mL/min. The mobile phase was (A): 10 mM CH_3_COONH_4_ in H_2_O/ACN (95:5 *v/v %*) and (B): 10 mM CH_3_COONH_4_ in H_2_O/ACN (5:95 *v/v %*). The following gradient was employed: 0 min, 100% B, isocratic for 1.5 min, 1.51-3.5 min, 100-70% B, 3.51-9 min, 70-50% B, 9.01-9.50 min, 50-20% B, isocratic for 2 min, returning to 100% in 0.1 min and 4 minutes for column re-equilibration. 5 µL were injected. ESI^-^ analysis was carried on Acquity UPLC HSS T3 column (150 × 2.1 mm; 1.8 µm, 100Å) protected with Acquity UPLC HSS T3 VanGuard Pre-column (50 × 2.1 mm; 1.8 µm, 100Å). Oven temperature was set to 45°C and 5 µL were injected. The mobile phases employed were H_2_O (A) and ACN (B) both acidified at 0.1% HCOOH (*v/v %*). The following gradient was used: starting at 0% B; isocratic for 1 min, 0% B; 1-1.5 min, 0-20% B; 1.5-6 min, 25-70% B; 6-8 min, 70-80% B; 8-9 min, 80-98% B and then 4 minutes for column re-equilibration. Samples were dissolved in 50 µL of ACN 70% (*v/v %*) and MeOH 30% (*v/v %*), respectively, for positive and negative analysis.

Source parameters: Nebulizer gas (N_2_) pressure: 3.0 Bar, Dry gas (N_2_): 10 L/min, Dry temperature: 220°C. Mass spectra were recorded in the range m/z 50–700, with a total cycle time was of 0.75 s. MS Spectra Rate and MS/MS Spectra rate were set, respectively, to 4 and 12 Hz. Precursors for data-dependent acquisition with a normalized threshold above 200 counts/1000 scans were fragmented with a MultiCE approach (20-80%). Exclusion time was set to 1 min. The instrument was calibrated with a NaFormate solution with the following composition: positive mode, m/z: 90.9766, 158.9641, 226.9515, 294.9389, 362.9263, 430.9138, 498.9012, 566.8886, 634.8760; negative mode, m/z: 112.9854, 180.9730, 248.9604, 316.9478, 384.9353, 452.9227, 520.9101, 588.8975, 656.8850.

**UHPLC-TIMS-MS data analysis, pre-processing and annotation**

TimsTOF-MS data analysis was performed with MetaboScape 2021 (Bruker) employing a feature finding algorithm (T-Rex 4D for lipidomics and T-Rex 3D for metabolomics) that automatically extracts buckets from raw files. At the beginning of each LC-MS run, a mixture (1:1 v/v %) of 10 mM sodium formate calibrant solution and ESI-L Low Concentration Tuning Mix was injected to recalibrate, respectively, the mass and mobility data. Metabolomics raw files were recalibrated exclusively with NaFormate. Lipids feature detection was set to 400 and 150 counts for positive and negative mode. Metabolites feature detection was set to 1000 and 250 counts for positive and negative, respectively. The minimum number of data points in the 4D-TIMS space was set to 100, and recursive feature extraction was used (75 points). Lipid annotation was performed first with a rule-based annotation, based on characteristic fragments and their intensity in acquired MS/MS spectra, and, subsequently, using the LipidBlast spectral library of MS DIAL (<http://prime.psc.riken.jp/compms/msdial/main.html>) with the following parameters: Mass accuracy: narrow 2 ppm, wide 10 ppm; mSigma: narrow 30, wide 250, MS/MS score: narrow 800, wide 150. Collision Cross-Section (CCS)%: narrow 2, wide 3.5. The spectra were processed in positive mode using [M+H]^+^, [M+Na]^+^, [M+K]^+^, [M+H–H_2_O]^+^ and [M+NH_4_]^+^ ions, while [M–H]^-^, [M+Cl]^-^, [M+CH_3_COO]^-^ and [M–H_2_O]^-^ in negative mode. CCS values were compared with those predicted by CCSbase platform (<https://ccsbase.net/predictions>), the assignment of the molecular formula was performed for the detected features using Smart Formula™ (SF). Manual curation of each lipid was then performed following Lipidomics Standard Initiative (LSI) guidelines (https://lipidomics-standards-initiative.org/guidelines/lipid-species-identification/general-rules), specifically, besides MS/MS diagnostic ions, crucial aspects of lipid annotation such as (a) lipid adducts in electrospray ionization and (b) regular retention behavior, e.g., the equivalent carbon number (ECN) model used for RPLC, were carefully evaluated.LipidCreator tool (<https://lifs-tools.org/lipidcreator.html>) extension in the Skyline (<https://skyline.ms/project/home/begin.view>) as used for in silico comparison of specific product ions for manual MS/MS curation. Polar metabolites were annotated using the following libraries of MS-DIAL (http://prime.psc.riken.jp/compms/msdial/main.html): MSMS-Public-Pos and MSMS-Public-Neg. The following parameters were adopted: Mass accuracy: narrow 2 ppm, wide 10 ppm; mSigma: narrow 30, wide 250, MS/MS score: narrow 800, wide 150. Spectra were processed in positive mode using [M+H]^+^ as primary ion and [M+Na]^+^, [M+K]^+^, [M+H–H_2_O]^+^ as seed ions while, in negative mode, [M–H]^−^ was the primary ion and [M+Cl]^−^, [M–H_2_O]^−^ were the seed ions. All spectra were manually curated and investigated. Subsequently, all metabolites missing in more than the 75% of real samples and 50% of QCs samples were excluded. In addition, the polar and apolar molecules with a coefficient of variation (CV) higher than the 30% among QCs were discarded.

**Section S2**

**Maternal Characteristic Data Imputation**

Missing data was present for both cohorts due to incompletion of questionnaires and/or unavailability of clinical records. Prior to statistical comparison, missing data imputation was performed. Mode was the method of data imputation for categorical data and median for continuous data. Statistical comparisons were performed using Stata v17.0 (StataCorp LLC, Texas, USA).

**Lipidomic and Metabolomic Data Pre-processing**

Firstly, data for annotated metabolites and lipids were pre-processed independently for all four modalities: lipidomics positive and negative ionization, and metabolomics positive and negative ionization. The lipidomics datasets were normalized using an internal standard, while the metabolomics datasets were normalized based on the total ion sum. Next, the four datasets underwent the same treatment. Missing values and zeros were replaced with one-fifth of the minimum value recorded in the dataset for that molecule. Logarithm values were then calculated using a base of 10. Prior to further chemometric modelling, the data were scaled using autoscaling. This involved centering each variable by subtracting its average from the data and then scaling it by dividing it by its standard deviation.

**Lipidomic and Metabolomic Data Analysis**

Multivariate data analysis was conducted on the filtered dataset using custom-developed routines and standard functions in Matlab R2022b (The MathWorks Inc, Natick, MA, USA). In this experiment, the same biological sample was analysed using different omics methods, resulting in a multi-block dataset. These blocks correspond to variables measured on the same samples using different analytical platforms. By employing multi-block methodologies, it becomes possible to extract relevant information from all the blocks concurrently, eliminating the need to build separate models for each dataset. In this study, four matrices were created based on the technologies employed for data generation.

Data fusion, also known as multiblock analysis, was applied as a chemometric strategy to integrate multiple sources of information (blocks) and maximize the extraction of information from various omics approaches concurrently. The objective of this approach was to enhance model performance in terms of robustness, consistency, and accuracy. The original pre-processed data, which shared the same sampling mode, were concatenated row-wise, enabling a comprehensive analysis of the combined dataset. (<https://doi.org/10.1002/cem.811>).

The data analysis was performed in two ways: independently on each dataset and by employing both low-level and mid-level data fusion approaches.

**Exploratory tools: Principal Component Analysis (PCA) and SUM-PCA**

After performing column autoscaling, the data sets underwent principal component analysis (PCA). PCA is a statistical technique used for unsupervised data reduction and visualization. It represents the variance of the data matrix using orthogonal variables called "Principal components" (PCs), which capture the maximum variance. These PCs define a new space to represent samples, highlighting similarities and dissimilarities between them. To interpret the observed differences, the loadings (the cosine of the angle between each PC and the original variables) plots were examined. To visualise the effectiveness of preliminary differentiation, Hotelling (T^2^) confidence ellipses were added to the score plots. These T^2^ confidence ellipses were calculated independently for each class, with a confidence level set at 95%.

SUM-PCA, is a basic multiblock analysis technique that applies PCA to the low-level fused data blocks. Each block is modelled with identical super scores (T_sup_), but specific block loadings (P_b_) and residuals (E_b_). T_sup_ represents the best summary of all measured block characteristics and can be considered the consensus score. The regression of the consensus scores on the combined block score matrix expresses the contribution of each block to the consensus, which is represented by the block weight matrix (W). W provides information about the contribution of each block to the consensus for each principal component ([**https://doi.org/10.1002/cem.811**](https://doi.org/10.1002/cem.811)).

**Chemometric classification models**

**Training and test set split, model evaluation**

To ensure the reliability and comparability of the classification models, the Duplex algorithm was employed to establish common training and test sets for all omics modalities. The Duplex algorithm is a sample-splitting method. The Duplex algorithm operates by initially selecting the two most distant samples and assigning them to the training set, while the next two most distant samples are assigned to the test set. The procedure continues by iteratively adding samples to either the training or test set based on their dissimilarity to the samples already selected, using the maximin criterion. The maximin criterion quantifies how different a sample is from all the objects in a particular set, and the sample with the maximum value of the minimum distance to all the objects in a set is added to that set (<http://dx.doi.org/10.1080/00401706.1977.10489581>;<https://doi.org/10.1016/S0003-2670(02)00651-7>).

In the current study, the Duplex algorithm was applied to the super scores derived from SUMPCA. To achieve equal representation of both categories, the algorithm was independently implemented to the two classes (sCHD and control) using a 70:30 splitting ratio. This methodology ensured that the training and test sets effectively covered the sample space while maintaining equal representativeness.

The performance of each model was assessed using confusion matrices, which provide detailed information about the types of errors made. To determine the accuracy of class predictions, two parameters were employed for each class: True Positives (TP), representing the number of samples from the category of interest correctly classified as belonging to that class, and True Negatives (TN), which denotes the number of samples from other categories correctly predicted as not belonging to the class. Conversely, False Positives (FP) refers to the number of samples from other classes that are erroneously assigned to the class of interest, while False Negatives (FN) refers to the number of samples from the specific category not predicted as belonging to that class. When expressed as percentages, TP and TN are defined as Sensitivity and Specificity, respectively, while Accuracy correspond to the overall fraction of correctly classified samples. These metrics provide insights into the performance of the models in terms of correctly identifying the respective classes.

**Partial Least Squares-Discriminant Analysis (PLS-DA) and low-level fusion**

To classify the two different classes (pathological and healthy), Partial Least Squares-Discriminant Analysis (PLS-DA) was individually applied to each dataset. Additionally, the optimal model was applied to the genetic datasets as external data sources. Furthermore, PLS-DA was performed at the low-level data integration stage. This involved concatenating the experimental matrices corresponding to the different data blocks, typically after scaling each block by its Frobenius' norm to ensure a balanced contribution (<https://doi.org/10.1016/B978-0-444-63984-4.00002-8>). The objective was to classify and identify the relevant variables in terms of severity.

PLS-DA is a supervised classification algorithm commonly used with mass spectrometry datasets. It employs PLS regression on a binary-coded categorical variable (Y) for classification purposes. The matrix X contains independent spectra, while Y represents class membership through a binary matrix with each class assigned a specific code (e.g., [1 0 0 0] for the first class, [0 1 0 0] for the second class, and so on). The PLS-DA algorithm establishes the best relationship between X and the categorical response variable Y. Classification is performed using linear discriminant analysis (LDA) applied to the predicted Y or the PLS values. PLS-DA was independently performed on each modality. The Duplex algorithm was used to establish common training and test sets for all omics modalities utilising as input the super scores derived from SUM-PCA ([**https://doi.org/10.1016/S0003-2670(02)00651-7**](https://doi.org/10.1016/S0003-2670(02)00651-7)**).** The optimal number of latent variables (LV) was determined to minimize misclassification errors and maximize accuracy through cross-validation (Venetian blinds 5 folds). (<https://doi.org/10.1016/0003-2670(86)80028-9>; [**https://doi.org/10.1002/cem.1180010306**](https://doi.org/10.1002/cem.1180010306) ).

**Sequential and orthogonalized covariance selection (SO-COVSEL-LDA)**

A limitation of low-level multi-block methods is the lack of clear explanation regarding the variables contributing to the common and distinct variability across different data blocks. Variable selection is particularly challenging, especially when redundant information is present in multiple data blocks and the objective is to utilize complementary information that improves predictive performance. In such cases, novel multi-block methods like sequential and orthogonalized covariance selection (SO-CovSel) ([**https://doi.org/10.1002/cem.3120**](https://doi.org/10.1002/cem.3120)) can be effectively employed. SO-CovSel is a sequential ([**https://doi.org/10.1002/cem.811**](https://doi.org/10.1002/cem.811)) mid-level multi-block algorithm. This algorithm, combined with linear discriminant analysis (LDA) (<https://doi.org/10.1016/j.chemolab.2014.12.001>), performs variable selection while building the classification model. SO-CovSel is closely related to covariance selection (CovSel) (<https://doi.org/10.1016/j.chemolab.2010.10.003>) and shares some advantages with SO-PLS (<https://doi.org/10.1002/cem.1357>;<https://doi.org/10.1016/j.chemolab.2013.03.006>), but it is specifically designed for variable selection and provides direct information about the key variables driving the model. SO-PLS, a multi-block method, sequentially incorporates information from each data matrix into the regression model. Similarly, SO-CovSel incorporates information from different data blocks while eliminating redundancies through orthogonalization. CovSel, on the other hand, is a feature selection approach that identifies the most relevant variables by estimating their covariances with the response variable. In the SO-CovSel algorithm, information is sequentially extracted from each data block using CovSel, and the selected variables are used for predicting the response. This method is computationally efficient and can handle any number of data blocks. SO-CovSel was chosen for this study because it ensures a parsimonious selection of biomarkers while maintaining excellent prediction accuracy.

To facilitate comparison, 64 SO-CovSel-LDA models were constructed using all possible combinations of the four blocks. Similar to the approach used for PLS-DA, the Duplex algorithm was used to establish common training and test sets for all omics modalities, however, and model selection was performed by cross-validation.

**Metabolite Set Enrichment Analysis (MSEA)**

Metabolite set enrichment analysis was performed utilising MetaboAnalyst 5.0. Analysis was performed on analytes identified through PLS-DA and SO-CovSel-LDA modelling. Over representation analysis was utilised; with SMPDB pathway-based compound lists for metabolites. Metabolite sets containing at least 2 entries were used. All compounds in the selected library were used as a reference metabolome. It was not possible to utilise the reference metabolome fom our analytical platform due to over half of uploaded IDs unable to be matched to database.

**Supplementary Tables**

**Supplementary Table 1**

*EUROCAT classification of congenital heart disease (CHD). Diagnoses are classified as severe, non-severe or other with associated anatomical descriptions. Adapted from EUROCAT 2013.*

| **Congenital Heart Defect** | **Anatomical Description** |
| --- | --- |
| ***Classified as severe CHD*** | |
| Common arterial trunk (truncus arteriosus) | Single arterial vessel originating from the ventricles with a large subvulvur septal defect. Aortic arch, pulmonary and coronary arteries originate from this common vessel. |
| Double outlet right ventricle (DORV) | Aorta and pulmonary artery originate from the right ventricle. |
| Transposition of great arteries (TGA) | Aorta originates from the right ventricle and pulmonary artery from the left ventricle. |
| Single ventricle/common ventricle/double inlet left ventricle (DILV) | Absence of ventricular septum, with single ventricle. |
| Atrioventricular septal defect (AVSD) | Central defect of the cardiac septa and a common atrioventricular valve. |
| Tetralogy of Fallot (ToF) | Ventricular septal defect, overriding aorta, pulmonary valve stenosis and right ventricular hypertrophy. |
| Tricuspid atresia/stenosis | Triscupid valve obstruction with right ventricular hypoplasia. |
| Ebstein’s anomaly | Tricuspid valve displacement with small right ventricle and large right atrium. |
| Pulmonary valve atresia | Complete obstruction at the pulmonary valve preventing flow of blood to the pulmonary artery. |
| Aortic valve atresia/stenosis | Occlusion or narrowing of the aortic valve. |
| Mitral valve anomalies | Atresia, stenosis, or insufficiency of the mitral valve. |
| Hypoplastic left heart (HLH) | Hypoplasia of the left ventricle, outflow, and ascending aorta due to left heart obstruction. |
| Hypoplastic right heart (HRH) | Hypoplasia of the right ventricle, associated with other cardiac malformation (e.g. Ebstein’s anomaly). |
| Coarctation of aorta (CoA) | Narrowing of the aorta at the ductus arteriosus insertion. |
| Aortic atresia/interrupted aortic arch | Atresia or interrupted connection of the aorta. |
| Total anomalous pulmonary venous return (TAPVD) | All 4 pulmonary veins drain into the right atrium or venous tributary. |
| ***Classified as non-severe CHD*** | |
| Discordant atrioventricular connection (congenitally corrected TGA) | Aorta and pulmonary artery are transposed alongside transposition of the morphological right and left ventricles and their atrioventricular valves. |
| Double outlet left ventricle (DOLV) | Aorta and pulmonary artery originate from the left ventricle. |
| Ventricular septal defect (VSD) | Defect in the septum separating the ventricles. |
| Atrial septal defect (ASD) | Defect in the septum separating the atria. |
| Pulmonary valve stenosis | Narrowing of the pulmonary valve. |
| Atrial isomerism | Right isomerism – two morphological right atria; left isomerism – two morphological left atria. |
| Patent ductus arteriosus (PDA) as only CHD in term infant | Open duct >6months age requiring interventional closure. |
| ***Other*** |  |
| Unspecified cardiac anomaly | Cardiac diagnosis certain, but completely unknown. |

**Supplementary Table 2**

*Maternal and child characteristic variable descriptions and source data. MQ – maternal questionnaire; MR – maternal medical records; RC – research clinic; SD – sample database; CHR – child health record.*

| **Variable Name** | **Source Data** | | **Variable Description** | **Additional Notes** |
| --- | --- | --- | --- | --- |
|  | ***ALSPAC*** | ***cOMACp*** |  |  |
| Ethnic Group | MQ | MQ  MR | Ethnic group of mother. |  |
| Child Gender | CHR | CHR | Gender of biological child. |  |
| BMI | MQ  RC | MQ  MR | BMI of the mother before or during the index pregnancy.  *cOMACp: BMI obtained from maternal pregnancy record if self-reported pre-pregnancy questionnaire data was missing.*  *ALSPAC: BMI at first pregnancy clinic visit preferentially used. If missing, late pregnancy clinic used. If both clinic measures missing, self-reported pre-pregnancy questionnaire data used.* | Pre- and during index pregnancy time point chosen for comparison as no data for BMI at time of blood sampling in cOMACp.  For ALSPAC due to data availability for multiple clinic time points, these data preferentially used over self-reported BMI. This has a higher accuracy as weight and height measured by a clinician. |
| Age at Sampling | SD | SD | Age of the mother at the time of blood sampling (years). | For ALSPAC age at sampling calculated by adding number of full years completed (age of child in months at time of sampling known) at time of sampling to age at delivery. Potential error of 1 year. |
| Sample Timing | SD | SD | Timing of maternal blood sample (months postnatal). | For cOMACp, calculated from child’s date of birth and data of sampling, rounded to the nearest month postnatal.  For ALSPAC, date of birth and date of sampling not available. Therefore, utilised age of child (completed months postnatal). |

**Supplementary Table 3**

| **Variable Name** | **Baseline Method** | **Baseline Value** | | |
| --- | --- | --- | --- | --- |
|  |  | **ALSPAC**  ***Missing n (%)*** | **cOMACp Structural**  ***Missing n (%)*** | **cOMACp Genetic**  ***Missing n (%)*** |
| Ethnic Group | Mode | White  *52 (32.10%)* | White  *26 (16.56%)* | White  *10 (30.30%)* |
| Child Gender | Mode | Male  *1 (0.62%)* | Male  *0 (0.00%)* | Male  *0 (0.00%)* |
| BMI | Median | 24.1  *10 (6.17%)* | 25.175  *35 (22.29%)* | 24.1  *12 (36.36%)* |
| Age at Sampling | Median | 24  *28 (17.28%)* | 35  *3 (1.91%)* | 39  *0 (0.00%)* |
| Sample Timing | Median | 37  *1 (0.62%)* | 27  *0 (0.00%)* | 12  *0 (0.00%)* |

*Maternal and child characteristic variable methods of data imputation for missing data. Baseline imputation value and number (n) and proportion (%) of missing values shown for each variable by cohort.*

*Missing data was present for cOMACp and ALSPAC due to incompletion of questionnaires and/or unavailability of clinical records. Prior to statistical comparison, missing data imputation was performed. Mode was the method of data imputation for categorical data and median for continuous data.

**Supplementary Table 4**

*Maternal and child characteristic variables following imputation of missing data gCHD vs. control. Number (proportion) presented for categorical variables and median (IQR) presented for continuous variables. Where a cell count is less than 5, this is represented as <5. Cell counts <5 may include 0. P value calculated by chi-squared test ^a^ for categorical variables and Wilcoxon Rank Sum (Mann-Whitney) Test^b^ for continuous variables. gCHD – genetic congenital heart disease.*

| **Maternal Characteristic** | **gCHD (n=33)** | **Control (n=162)** | **P Value** |
| --- | --- | --- | --- |
| **Ethnic Group *n (%)*** | | | |
| White | 32 (96.97%) | 158 (97.53%) | 0.057 ^a^ |
| Mixed/multiple ethnic groups | <5 | <5 |  |
| Asian or Asian British | <5 | <5 |  |
| Black/African/Caribbean/black British | <5 | <5 |  |
| **Child Gender *n (%)*** | | | |
| Female | 14 (42.42%) | 74 (45.68%) | 0.732 ^a^ |
| Male | 19 (57.58%) | 88 (54.32%) |  |
| **BMI *median (IQR)*** | 24.1 (23.5-25.01) | 24.1 (21.37-28.70) | 0.758 |
| **Age at Sampling *median (IQR)*** | 39 (33-40) | 24 (23-26) | <0.001 ^b^ |
| **Sample Timing *Median (IQR)*** | 12 (6-65) | 37 (36-39) | 0.016 ^b^ |

**Supplementary Table 5**

*Breakdown of CHD diagnoses in sCHD and gCHD groups, stratified by IPCCC/ICD-11 classification.*

|  | **sCHD** | **gCHD** |
| --- | --- | --- |
| **Congenital anomaly of the great arteries including the arterial duct** | **130** | **26** |
|  | | |
| aberrant subclavian artery | 7 | <5 |
| aortic arch hypoplasia | 8 | <5 |
| aortic root dilation | <5 | <5 |
| coarctation of aorta | 31 | <5 |
| common origin innominate artery and left common carotid | <5 | <5 |
| interrupted aortic arch | <5 | <5 |
| major aortopulmonary collateral arteries | <5 | <5 |
| patent ductus arteriosus | 53 | 12 |
| pulmonary arterial stenosis/hypoplasia/dysplasia | 6 | <5 |
| retro-aortic innominate vein | <5 | <5 |
| right aortic arch | 9 | <5 |
| vascular ring | 9 | <5 |
|  | | |
| **Congenital anomaly of an atrium or atrial septum** | **75** | **21** |
|  | | |
| atrial septal defect | 42 | 12 |
| patent foramen ovale | 31 | 9 |
| right atrial appendage aneurysm | <5 | <5 |
| right atrial dilation | <5 | <5 |
|  | | |
| **Congenital anomaly of a ventriculo-arterial valve or adjacent regions** | **74** | **12** |
|  | | |
| absent pulmonary valve | <5 | <5 |
| aortic atresia | <5 | <5 |
| aortic regurgitation | <5 | <5 |
| aortic stenosis | 11 | <5 |
| bicuspid aortic valve | 22 | <5 |
| bicuspid pulmonary valve | <5 | <5 |
| pulmonary atresia | 9 | <5 |
| pulmonary regurgitation | 6 | <5 |
| pulmonary stenosis | 16 | 6 |
| subaortic stenosis | <5 | <5 |
|  | | |
| **Congenital anomaly of a ventricle or the ventricular septum** | **71** | **14** |
|  | | |
| left ventricular hypoplasia | <5 | <5 |
| right ventricular hypoplasia | 8 | <5 |
| tetralogy of Fallot | 11 | <5 |
| ventricualr septal defect | 51 | 7 |
|  | | |
| **Congenital anomaly of an atrioventricular valve or atrioventricular septum** | **45** | **16** |
|  | | |
| atrioventricular septal defect | 7 | 9 |
| Ebstein's anomaly | <5 | <5 |
| mitral regurgitation | 9 | <5 |
| mitral stenosis | 5 | <5 |
| mitral valve abnormality (other) | <5 | <5 |
| tricuspid regurgitation | 14 | <5 |
| tricuspid stenosis | <5 | <5 |
|  | | |
| **Congenital anomaly of an atrio-ventricular or ventriculo-arterial connection** | **26** | **<5** |
|  | | |
| bicuspid truncal valve | <5 | <5 |
| double outlet left ventricle | <5 | <5 |
| double outlet right ventricle | 8 | <5 |
| transposition of the great arteries (congenitally corrected) | <5 | <5 |
| transposition of the great arteries | 10 | <5 |
| truncus arteriosus | <5 | <5 |
|  | | |
| **Functionally univentricular heart** | **23** | **<5** |
|  | | |
| double inlet left ventricle | 6 | <5 |
| hypoplastic left heart syndrome | 10 | <5 |
| mitral atresia | <5 | <5 |
| tricuspid atresia | 5 | <5 |
|  | | |
| **Congenital anomaly of mediastinal vein** | **17** | **<5** |
|  | | |
| interrupted inferior caval vein with absent suprarenal segment and azygos continuation | <5 | <5 |
| left superior caval vein | 10 | <5 |
| partial anomalous pulmonary venous drainage | <5 | <5 |
| pulmonary vein abnormality | <5 | <5 |
| total anomalous pulmonary venous drainage | <5 | <5 |
|  | | |
| **Congenital anomaly of position or spatial relationships of the thoraco-abdominal organs** | **<5** | **<5** |
|  | | |
| dextrocardia | <5 | <5 |
| left isomerism | <5 | <5 |
|  | | |
| **Congenital anomaly of the coronary artery** | **<5** | **<5** |
|  | | |
| coronary arterial abnormality | <5 | <5 |

**Supplementary Table 6**

RP-UHPLC-TIMS-Q-TOF based annotation of lipids. RT: retention time; m/z meas: mass to charge measured; M meas.: measured mass; CCS: cross collisional section; Mob.: mobility; Primary ion has been used for normalization and additional adducts, if detected, were reported in round brackets. Abbreviations: cholesteryl esters (CE), ceramides (Cers), diacylglycerols (DG), ganglioside GM3 (GM3), hexosylceramides (Hexcers), dihexosylceramide (Hex2cer), lysophosphatidylcholines (LPC), ether-linked lysophosphatidylcholines (LPC-O) lysophosphatidylethanolamines (LPE), phosphatidylcholines (PC), ether-linked phosphatidylcholine (PC-O), phosphatidylethanolamines (PE), ether-linked phosphatidylethanolamine (PE-P), phosphatidylinositols (PIs), phosphatidylserine (PS), sphingomyelins (SM), triacylglycerols (TG), letters A and B indicate possible isomers.

| **RT [min]** | **CCS (Å²)** | **Mob. 1/K_0_** | **m/z meas.** | **M meas.** | **Ions** | **Name** | **Molecular Formula** | **MS/MS score** | **Δm/z [ppm]** | **ΔCCS [%]** |
| --- | --- | --- | --- | --- | --- | --- | --- | --- | --- | --- |
| 1.81 | 219.3 | 1.061 | 431.3881 | 430.38082 | [M+H]^+^ | α-tocopherol | C_29_H_50_O_2_ | 980.3 | 0.81 | 0.8 |
| 0.34 | 204.3 | 0.984 | 370.2961 | 369.28882 | [M+H]^+^ | CAR 14:1 | C_21_H_39_NO_4_ | 915.1 | 0.66 | 2.5 |
| 0.47 | 216.5 | 1.045 | 400.34384 | 399.33656 | [M+H]^+^ | CAR 16:0 | C_23_H_45_NO_4_ | 897.3 | 4.24 | 0.1 |
| 0.61 | 224.2 | 1.085 | 428.37519 | 427.36791 | [M+H]^+^ | CAR 18:0 | C_25_H_49_NO_4_ | 878.8 | 4.1 | 0.1 |
| 0.49 | 218.7 | 1.058 | 426.35925 | 425.35198 | [M+H]^+^ | CAR 18:1 | C_25_H_47_NO_4_ | 911.1 | 1.03 | 0.02 |
| 0.42 | 213.7 | 1.034 | 424.34152 | 423.33424 | [M+H]^+^ | CAR 18:2 | C_25_H_45_NO_4_ | 861.3 | 0.43 | 0.04 |
| 3.29 | 284.1 | 1.389 | 642.61767 | 624.58486 | [M+NH_4_]^+^, ([M+K]^+^, [M+Na]^+^) | CE 16:0 | C_43_H_76_O_2_ | 986.9 | -1.075 | 1.8 |
| 3.30 | 286.6 | 1.402 | 656.63282 | 638.59941 | [M+NH_4_]^+^, ([M+Na]^+^) | CE 17:0 | C_44_H_78_O_2_ | 969.2 | -1.744 | 1.9 |
| 3.24 | 285.9 | 1.398 | 654.61878 | 636.58496 | [M+NH_4_]^+^ | CE 17:1 | C_44_H_76_O_2_ | 985.8 | 0.593 | 2.1 |
| 3.28 | 289.3 | 1.415 | 668.63434 | 650.60052 | [M+NH_4_]^+^ | CE 18:1 | C_45_H_78_O_2_ | 871.6 | 0.512 | 2.1 |
| 3.21 | 289.7 | 1.417 | 666.6191 | 648.58528 | [M+NH_4_]^+^ | CE 18:2 | C_45_H_76_O_2_ | 983.5 | 0.983 | 2.7 |
| 3.08 | 289.3 | 1.415 | 662.58695 | 644.55312 | [M+NH_4_]^+^ | CE 18:4 | C_45_H_72_O_2_ | 967.6 | -0.197 | 4.5 |
| 3.30 | 292.8 | 1.433 | 682.64785 | 664.61402 | [M+NH_4_]^+^ | CE 19:1 | C_46_H_80_O_2_ | 301.8 | -2.746 | 2.5 |
| 3.17 | 293.0 | 1.434 | 690.61825 | 672.58402 | [M+NH_4_]^+^, ([M+K]^+^, [M+Na]^+^, [M+H]^+^) | CE 20:4 | C_47_H_76_O_2_ | 978.6 | -0.063 | 2.5 |
| 3.13 | 295.1 | 1.446 | 714.61796 | 696.58423 | [M+NH_4_]^+^, ([M+Na]^+^) | CE 22:6 | C_49_H_76_O_2_ | 966.5 | -0.55 | 2.3 |
| 2.53 | 272.9 | 1.333 | 608.59853 | 607.59126 | [M+H]^+^ | Cer 16:1;2O/23:0 | C_39_H_77_NO_3_ | 822.1 | 1.517 | 1.0 |
| 2.42 | 270.1 | 1.318 | 594.58495 | 593.57658 | [M+H]^+^, ([M+Na]^+^, [M+H-H_2_O]^+^) | Cer 18:1;2O/20:0 | C_38_H_75_NO_3_ | 930.0 | 4.939 | 1.2 |
| 2.64 | 278.4 | 1.36 | 622.61375 | 621.60605 | [M+H]^+^, ([M+Na]^+^, [M+H-H_2_O]^+^, [M+K]^+^) | Cer 18:1;2O/22:0 | C_40_H_79_NO_3_ | 949.8 | 0.816 | 2.0 |
| 2.72 | 279.6 | 1.366 | 636.62837 | 635.62137 | [M+H]^+^, ([M+Na]^+^, [M+K]^+^, [M+H-H_2_O]^+^) | Cer 18:1;2O/23:0 | C_41_H_81_NO_3_ | 941.2 | -0.914 | 2.1 |
| 2.80 | 282.7 | 1.382 | 650.64416 | 649.63832 | [M+H]^+^, ([M+Na]^+^, [M+K]^+^, [M+H-H_2_O]^+^) | Cer 18:1;2O/24:0 | C_42_H_83_NO_3_ | 960.9 | -0.596 | 2.2 |
| 2.63 | 280.5 | 1.372 | 648.62889 | 647.62232 | [M+H]^+^, ([M+K]^+^, [M+Na]^+^) | Cer 18:1;2O/24:1 | C_42_H_81_NO_3_ | 944.3 | -0.081 | 1.9 |
| 2.85 | 285.3 | 1.396 | 664.65958 | 663.65102 | [M+H]^+^, ([M+Na]^+^, [M+H-H_2_O]^+^) | Cer 18:1;2O/25:0 | C_43_H_85_NO_3_ | 608.9 | 0.3 | 1.2 |
| 2.44 | 272.0 | 1.329 | 620.59914 | 619.59186 | [M+H]^+^ | Cer 18:2;2O/22:0 | C_40_H_77_NO_3_ | 862.1 | 2.436 | 0.4 |
| 2.56 | 275.2 | 1.345 | 634.6136 | 633.60519 | [M+H]^+^, ([M+Na]^+^) | Cer 18:2;2O/23:0 | C_41_H_79_NO_3_ | 866.7 | 0.527 | 0.7 |
| 2.44 | 275.5 | 1.347 | 646.61127 | 645.60437 | [M+H]^+^, ([M+Na]^+^) | Cer 18:2;2O/24:1 | C_42_H_79_NO_3_ | 870.7 | -2.956 | 1.0 |
| 2.69 | 282.6 | 1.382 | 662.64576 | 661.63848 | [M+H]^+^ | Cer 18:2;2O/25:0 | C_43_H_83_NO_3_ | 121.6 | 1.6 | 1.0 |
| 2.39 | 264.9 | 1.294 | 612.55608 | 594.52219 | [M+NH_4_]^+^, ([M+H]^+^, [M+Na]^+^, [M+H-H_2_O]^+^, [M+K]^+^) | DG 16:0_18:1 | C_37_H_70_O_5_ | 963.6 | -0.042 | 1.0 |
| 2.26 | 260.0 | 1.27 | 610.53987 | 592.50605 | [M+NH_4_]^+^ | DG 16:1_18:1 | C_37_H_68_O_5_ | 848.4 | -1.062 | 0.8 |
| 2.01 | 255.6 | 1.248 | 608.52377 | 590.49256 | [M+NH_4_]^+^, ([M+K]^+^, [M+Na]^+^) | DG 16:1_18:2 | C_37_H_66_O_5_ | 452.0 | -1.169 | 0.7 |
| 2.61 | 271.0 | 1.325 | 640.58672 | 622.55338 | [M+NH_4_]^+^, ([M+Na]^+^, [M+K]^+^) | DG 18:0_18:1 | C_39_H_74_O_5_ | 919.0 | -1.127 | 2.3 |
| 2.41 | 267.4 | 1.307 | 638.57141 | 620.53833 | [M+NH_4_]^+^, ([M+H]^+^, [M+H-H_2_O]^+^, [M+Na]^+^, [M+K]^+^) | DG 18:1_18:1 | C_39_H_72_O_5_ | 964.1 | -0.539 | 2.1 |
| 2.23 | 263.7 | 1.289 | 636.55585 | 618.52215 | [M+NH_4_]^+^, ([M+H-H_2_O]^+^, [M+K]^+^, [M+Na]^+^, [M+H]^+^) | DG 18:1_18:2 | C_39_H_70_O_5_ | 961.9 | -0.424 | 1.6 |
| 2.17 | 267.7 | 1.309 | 660.55613 | 642.51959 | [M+NH_4_]^+^, ([M+K]^+^, [M+Na]^+^) | DG 18:1_20:4 | C_41_H_70_O_5_ | 737.6 | -0.048 | 2.0 |
| 2.04 | 258.6 | 1.264 | 634.54036 | 616.5067 | [M+NH_4_]^+^, ([M+K]^+^, [M+Na]^+^) | DG 18:2_18:2 | C_39_H_68_O_5_ | 967.5 | -0.21 | 1.2 |
| 2.00 | 263.3 | 1.288 | 658.53857 | 640.50475 | [M+NH_4_]^+^ | DG 18:2_20:4 | C_41_H_68_O_5_ | 705.0 | -3.156 | 1.9 |
| 1.27 | 338.4 | 1.67 | 1151.70534 | 1152.71262 | [M-H]^-^ | GM3 34:1;2O | C_57_H_104_N_2_O_21_ | 990.1 | -0.416 | 0.8 |
| 1.46 | 344.1 | 1.698 | 1179.73704 | 1180.74431 | [M-H]^-^ | GM3 36:1;2O | C_59_H_108_N_2_O_21_ | 970.1 | 0.07 | 3.0 |
| 1.62 | 305.0 | 1.499 | 862.62492 | 861.61796 | [M+H]^+^, ([M+Na]^+^) | Hex2Cer 18:1;2O/16:0 | C_46_H_87_NO_13_ | 386.8 | -0.105 | 1.7 |
| 2.62 | 304.3 | 1.494 | 812.6943 | 811.68839 | [M+H]^+^, ([M+K]^+^, [M+Na]^+^) | HexCer 18:1;2O/24:0 | C_48_H_93_NO_8_ | 438.5 | -3.727 | 1.0 |
| 0.39 | 225.0 | 1.091 | 468.30901 | 467.30173 | [M+H]^+^ | LPC 14:0 | C_22_H_46_NO_7_P | 992.3 | 1.152 | 0.3 |
| 0.43 | 227.7 | 1.105 | 482.32479 | 481.31751 | [M+H]^+^ | LPC 15:0 | C_23_H_48_NO_7_P | 976.6 | 1.39 | 0.1 |
| 0.49 | 232.3 | 1.129 | 496.33988 | 495.3326 | [M+H]^+^ | LPC 16:0 | C_24_H_50_NO_7_P | 996.1 | 0.25 | 1.8 |
| 0.40 | 227.2 | 1.104 | 494.32469 | 493.31804 | [M+H]^+^, ([M+Na]^+^) | LPC 16:1_A | C_24_H_48_NO_7_P | 989.3 | 1.166 | 0.3 |
| 0.40 | 239.8 | 1.168 | 552.3314 | 493.31754 | [M+CH_3_COO]^-^ | LPC 16:1_B | C_24_H_48_NO_7_P | 971.4 | 1.236 | 3.4 |
| 0.54 | 236.4 | 1.15 | 510.35579 | 509.34881 | [M+H]^+^, ([M+Na]^+^) | LPC 17:0 | C_25_H_52_NO_7_P | 981.2 | 0.72 | 0.6 |
| 0.45 | 231.5 | 1.126 | 508.34075 | 507.33347 | [M+H]^+^ | LPC 17:1 | C_25_H_50_NO_7_P | 795.3 | 1.894 | 1.8 |
| 0.64 | 240.2 | 1.169 | 524.37118 | 523.3639 | [M+H]^+^ | LPC 18:0 | C_26_H_54_NO_7_P | 988.2 | 0.231 | 0.6 |
| 0.50 | 235.0 | 1.143 | 522.35591 | 521.34862 | [M+H]^+^, ([M+K]^+^, [M+Na]^+^) | LPC 18:1_A | C_26_H_52_NO_7_P | 987.3 | 0.995 | 0.1 |
| 0.50 | 246.7 | 1.203 | 580.36212 | 521.34827 | [M+CH_3_COO]^-^ | LPC 18:1_B | C_26_H_52_NO_7_P | 974.9 | 0.22 | 2.8 |
| 0.43 | 229.6 | 1.117 | 520.34003 | 519.33275 | [M+H]^+^ | LPC 18:2_A | C_26_H_50_NO_7_P | 997.2 | 0.47 | 1.1 |
| 0.42 | 243.9 | 1.19 | 578.34648 | 519.33263 | [M+CH_3_COO]^-^ | LPC 18:2_B | C_26_H_50_NO_7_P | 977.2 | 0.241 | 4.2 |
| 0.38 | 226.0 | 1.099 | 518.32441 | 517.31713 | [M+H]^+^ | LPC 18:3 | C_26_H_48_NO_7_P | 980.3 | 0.413 | 0.1 |
| 0.74 | 243.3 | 1.185 | 538.38759 | 537.38031 | [M+H]^+^ | LPC 19:0 | C_27_H_56_NO_7_P | 988.9 | 1.27 | 0.5 |
| 0.87 | 247.0 | 1.203 | 552.40252 | 551.39524 | [M+H]^+^ | LPC 20:0 | C_28_H_58_NO_7_P | 970.6 | 0.148 | 0.7 |
| 0.66 | 242.6 | 1.182 | 550.38712 | 549.37985 | [M+H]^+^ | LPC 20:1 | C_28_H_56_NO_7_P | 983.3 | 0.684 | 1.0 |
| 0.53 | 237.4 | 1.156 | 548.37092 | 547.36364 | [M+H]^+^ | LPC 20:2 | C_28_H_54_NO_7_P | 988.9 | -0.271 | 0.5 |
| 0.45 | 233.9 | 1.139 | 546.35423 | 545.34695 | [M+H]^+^ | LPC 20:3_A | C_28_H_52_NO_7_P | 963.2 | -2.16 | 0.01 |
| 0.45 | 248.7 | 1.214 | 604.36029 | 545.34644 | [M+CH_3_COO]^-^ | LPC 20:3_B | C_28_H_52_NO_7_P | 902.4 | -2.729 | 3.2 |
| 0.41 | 233.2 | 1.136 | 544.34001 | 543.33215 | [M+H]^+^, ([M+Na]^+^) | LPC 20:4_A | C_28_H_50_NO_7_P | 994.0 | 0.461 | 0.7 |
| 0.41 | 248.0 | 1.211 | 602.34661 | 543.33275 | [M+CH_3_COO]^-^ | LPC 20:4_B | C_28_H_50_NO_7_P | 979.0 | 0.411 | 3.6 |
| 0.37 | 228.9 | 1.115 | 542.32452 | 541.31725 | [M+H]^+^ | LPC 20:5 | C_28_H_48_NO_7_P | 990.9 | 0.8 | 0.3 |
| 0.40 | 235.6 | 1.148 | 568.33966 | 567.33181 | [M+H]^+^, ([M+Na]^+^) | LPC 22:6 | C_30_H_50_NO_7_P | 979.6 | -0.194 | 0.01 |
| 1.35 | 261.5 | 1.277 | 608.46677 | 607.4595 | [M+H]^+^ | LPC 24:0 | C_32_H_66_NO_7_P | 922.3 | 2.513 | 1.0 |
| 1.14 | 255.7 | 1.249 | 606.45009 | 605.44281 | [M+H]^+^ | LPC 24:1 | C_32_H_64_NO_7_P | 923.5 | 0.741 | 0.7 |
| 0.55 | 233.4 | 1.133 | 482.36072 | 481.35344 | [M+H]^+^ | LPC O-16:0 | C_24_H_52_NO_6_P | 418.4 | 0.393 | 0.7 |
| 0.74 | 240.8 | 1.171 | 510.39132 | 509.38405 | [M+H]^+^ | LPC O-18:0 | C_26_H_56_NO_6_P | 409.8 | -1.208 | 0.2 |
| 0.57 | 235.7 | 1.146 | 508.37677 | 507.36949 | [M+H]^+^ | LPC O-18:1 | C_26_H_54_NO_6_P | 418.4 | 1.062 | 0.6 |
| 1.25 | 256.8 | 1.253 | 592.47024 | 591.46297 | [M+H]^+^ | LPC O-24:1 | C_32_H_66_NO_6_P | 185.9 | 0.29 | 1.0 |
| 0.50 | 216.0 | 1.047 | 454.29202 | 453.28474 | [M+H]^+^ | LPE 16:0 | C_21_H_44_NO_7_P | 839.8 | -1.76 | 0.6 |
| 0.65 | 218.1 | 1.059 | 480.30977 | 481.31704 | [M-H]^-^ | LPE 18:0 | C_23_H_48_NO_7_P | 987.5 | 0.425 | 2.9 |
| 0.52 | 217.6 | 1.056 | 480.30897 | 479.3017 | [M+H]^+^ | LPE 18:1_A | C_23_H_46_NO_7_P | 976.1 | 1.092 | 0.01 |
| 0.52 | 215.1 | 1.044 | 478.29388 | 479.30115 | [M-H]^-^ | LPE 18:1_B | C_23_H_46_NO_7_P | 993.9 | -0.063 | 3.3 |
| 0.43 | 212.5 | 1.032 | 478.29372 | 477.28645 | [M+H]^+^ | LPE 18:2_A | C_23_H_44_NO_7_P | 979.1 | 1.888 | 0.4 |
| 0.43 | 212.6 | 1.032 | 476.27845 | 477.28573 | [M-H]^-^ | LPE 18:2_B | C_23_H_44_NO_7_P | 985.2 | 0.381 | 3.5 |
| 0.42 | 216.1 | 1.05 | 502.29367 | 501.2864 | [M+H]^+^ | LPE 20:4_A | C_25_H_44_NO_7_P | 961.5 | 1.622 | 0.3 |
| 0.42 | 217.4 | 1.057 | 500.27844 | 501.28571 | [M-H]^-^ | LPE 20:4_B | C_25_H_44_NO_7_P | 985.2 | 0.346 | 3.5 |
| 0.41 | 219.7 | 1.069 | 526.29347 | 525.28619 | [M+H]^+^ | LPE 22:6_A | C_27_H_44_NO_7_P | 971.8 | 1.279 | 0.2 |
| 0.41 | 221.5 | 1.078 | 524.27857 | 525.28584 | [M-H]^-^ | LPE 22:6_B | C_27_H_44_NO_7_P | 924.4 | 0.623 | 3.4 |
| 1.91 | 276.1 | 1.353 | 726.54105 | 725.53377 | [M+H]^+^ | LPE 36:4 | C_41_H_76_NO_7_P | 857.2 | -2.653 | 0.8 |
| 2.08 | 286.8 | 1.408 | 796.52336 | 795.51608 | [M+H]^+^ | LPE 42:11 | C_47_H_74_NO_7_P | 229.7 | -5.215 | 1.6 |
| 1.46 | 277.5 | 1.359 | 704.52266 | 703.5159 | [M+H]^+^, ([M+Na]^+^) | PC 12:0_18:1 | C_38_H_74_NO_8_P | 984.0 | 0.228 | 0.8 |
| 1.41 | 275.2 | 1.347 | 678.50653 | 677.49925 | [M+H]^+^ | PC 14:0_14:0 | C_36_H_72_NO_8_P | 928.6 | -0.647 | 0.9 |
| 1.48 | 287.6 | 1.411 | 788.54485 | 729.531 | [M+CH_3_COO]^-^ | PC 14:0_18:2_A | C_40_H_76_NO_8_P | 877.6 | 0.121 | 2.7 |
| 1.5 | 280.3 | 1.374 | 730.53878 | 729.53063 | [M+H]^+^, ([M+K]^+^, [M+Na]^+^) | PC 14:0_18:2_B | C_40_H_76_NO_8_P | 995.2 | 0.905 | 0.6 |
| 1.35 | 277.6 | 1.361 | 728.51973 | 727.51245 | [M+H]^+^ | PC 14:0_18:3 | C_40_H_74_NO_8_P | 994.8 | -3.817 | 1.0 |
| 1.45 | 290.8 | 1.428 | 812.5448 | 753.53095 | [M+CH_3_COO]^-^ | PC 14:0_20:4_A | C_42_H_76_NO_8_P | 778.6 | 0.086 | 3.1 |
| 1.46 | 283.8 | 1.391 | 754.53799 | 753.53115 | [M+H]^+^, ([M+Na]^+^) | PC 14:0_20:4_B | C_42_H_76_NO_8_P | 993.2 | -0.143 | 1.6 |
| 1.32 | 280.5 | 1.375 | 752.5224 | 751.51512 | [M+H]^+^ | PC 14:0_20:5 | C_42_H_74_NO_8_P | 995.8 | -0.341 | 0.7 |
| 1.39 | 286.5 | 1.406 | 778.53834 | 777.53106 | [M+H]^+^ | PC 14:0_22:6 | C_44_H_76_NO_8_P | 995.2 | 0.807 | 0.7 |
| 1.43 | 285.1 | 1.398 | 766.53826 | 765.53098 | [M+H]^+^ | PC 15:0_20:5 | C_43_H_76_NO_8_P | 995.9 | 0.13 | 1.2 |
| 1.52 | 290.0 | 1.423 | 792.55465 | 791.54708 | [M+H]^+^, ([M+Na]^+^) | PC 15:0_22:6 | C_45_H_78_NO_8_P | 993.1 | 1.089 | 0.79 |
| 1.87 | 286.5 | 1.404 | 734.56947 | 733.56229 | [M+H]^+^, ([M+Na]^+^) | PC 32:0 | C_40_H_80_NO_8_P | 997.8 | 0.043 | 1.2 |
| 1.85 | 289.9 | 1.423 | 792.57594 | 733.56209 | [M+CH_3_COO]^-^ | PC 16:0_16:0 | C_40_H_80_NO_8_P | 930.1 | -0.077 | 2.6 |
| 1.67 | 283.1 | 1.388 | 732.55377 | 731.54709 | [M+H]^+^, ([M+Na]^+^) | PC 16:0_16:1 | C_40_H_78_NO_8_P | 995.1 | 0.017 | 0.5 |
| 1.9 | 289.9 | 1.422 | 760.58579 | 759.57851 | [M+H]^+^ | PC 16:0_18:1_A | C_42_H_82_NO_8_P | 994.7 | 0.924 | 0.4 |
| 1.88 | 295.2 | 1.449 | 818.59269 | 759.57883 | [M+CH_3_COO]^-^ | PC 16:0_18:1_B | C_42_H_82_NO_8_P | 842.4 | 1.313 | 2.5 |
| 1.71 | 293.8 | 1.443 | 816.57744 | 757.56358 | [M+CH_3_COO]^-^ | PC 16:0_18:2_A | C_42_H_80_NO_8_P | 840.3 | 1.775 | 2.7 |
| 1.72 | 286.8 | 1.407 | 758.57027 | 757.563 | [M+H]^+^ | PC 16:0_18:2_B | C_42_H_80_NO_8_P | 995.7 | 1.11 | 0.5 |
| 1.54 | 291.3 | 1.43 | 814.56037 | 755.54651 | [M+CH_3_COO]^-^ | PC 16:0_18:3 | C_42_H_78_NO_8_P | 777.1 | 0.08 | 1.9 |
| 1.77 | 291.3 | 1.429 | 784.58534 | 783.57807 | [M+H]^+^ | PC 16:0_20:3_A | C_44_H_82_NO_8_P | 995.2 | 0.338 | 0.8 |
| 1.76 | 298.2 | 1.465 | 842.59195 | 783.57809 | [M+CH_3_COO]^-^ | PC 16:0_20:3_B | C_44_H_82_NO_8_P | 790.3 | 0.356 | 3.1 |
| 1.68 | 290.0 | 1.423 | 782.57008 | 781.5628 | [M+H]^+^ | PC 16:0_20:4_A | C_44_H_80_NO_8_P | 995.5 | 0.813 | 0.6 |
| 1.67 | 296.8 | 1.458 | 840.57692 | 781.56307 | [M+CH_3_COO]^-^ | PC 16:0_20:4_B | C_44_H_80_NO_8_P | 838.7 | 1.06 | 2.8 |
| 1.52 | 295.1 | 1.45 | 838.56024 | 779.54639 | [M+CH_3_COO]^-^ | PC 16:0_20:5_A | C_44_H_78_NO_8_P | 839.7 | -0.097 | 3.3 |
| 1.54 | 287.3 | 1.41 | 780.5543 | 779.54702 | [M+H]^+^ | PC 16:0_20:5_B | C_44_H_78_NO_8_P | 995.7 | 0.697 | 0.8 |
| 1.69 | 301.5 | 1.482 | 866.59204 | 807.57818 | [M+CH_3_COO]^-^ | PC 16:0_22:5 | C_46_H_82_NO_8_P | 771.2 | 0.457 | 3.2 |
| 1.62 | 292.3 | 1.435 | 806.5698 | 805.56252 | [M+H]^+^ | PC 16:0_22:6_A | C_46_H_80_NO_8_P | 994.3 | 0.454 | 0.5 |
| 1.61 | 300.3 | 1.476 | 864.57625 | 805.56239 | [M+CH_3_COO]^-^ | PC 16:0_22:6_B | C_46_H_80_NO_8_P | 819.1 | 0.283 | 3.1 |
| 1.56 | 283.3 | 1.389 | 756.55403 | 755.54676 | [M+H]^+^ | PC 16:1_18:2 | C_42_H_78_NO_8_P | 994.3 | 0.34 | 0.01 |
| 2.01 | 292.6 | 1.435 | 774.60045 | 773.59318 | [M+H]^+^ | PC 17:0_18:1 | C_43_H_84_NO_8_P | 995.8 | -0.389 | 0.6 |
| 2.13 | 295.3 | 1.449 | 788.61685 | 787.60957 | [M+H]^+^ | PC 18:0_18:1 | C_44_H_86_NO_8_P | 995.1 | 0.594 | 0.4 |
| 1.95 | 292.9 | 1.437 | 786.60135 | 785.59408 | [M+H]^+^ | PC 18:0_18:2 | C_44_H_84_NO_8_P | 995.1 | 0.77 | 0.59 |
| 2.24 | 297.9 | 1.462 | 802.63141 | 801.62414 | [M+H]^+^ | PC 18:0_19:1 | C_45_H_88_NO_8_P | 987.0 | -0.81 | 0.4 |
| 2.15 | 298.7 | 1.467 | 814.63145 | 813.62382 | [M+H]^+^, ([M+K]^+^, [M+Na]^+^) | PC 18:0_20:2_A | C_46_H_88_NO_8_P | 995.8 | -0.687 | 0.6 |
| 2.15 | 304.1 | 1.495 | 872.63901 | 813.62516 | [M+CH_3_COO]^-^ | PC 18:0_20:2_B | C_46_H_88_NO_8_P | 742.5 | 0.459 | 2.7 |
| 2.01 | 297.4 | 1.46 | 812.61675 | 811.60947 | [M+H]^+^ | PC 18:0_20:3_A | C_46_H_86_NO_8_P | 994.7 | 0.469 | 0.6 |
| 2.00 | 302.9 | 1.489 | 870.62331 | 811.60946 | [M+CH_3_COO]^-^ | PC 18:0_20:3_B | C_46_H_86_NO_8_P | 800.4 | 0.397 | 2.7 |
| 1.90 | 302.6 | 1.488 | 868.60786 | 809.59401 | [M+CH_3_COO]^-^ | PC 18:0_20:4_A | C_46_H_84_NO_8_P | 816.8 | 0.584 | 3.0 |
| 1.91 | 296.1 | 1.454 | 810.60076 | 809.59349 | [M+H]^+^ | PC 18:0_20:4_B | C_46_H_84_NO_8_P | 995.2 | 0.068 | 0.78 |
| 2.06 | 306.9 | 1.509 | 896.63799 | 837.62414 | [M+CH_3_COO]^-^ | PC 18:0_22:4_A | C_48_H_88_NO_8_P | 824.4 | -0.805 | 2.7 |
| 2.08 | 302.1 | 1.484 | 838.63136 | 837.62197 | [M+H]^+^, ([M+Na]^+^) | PC 18:0_22:4_B | C_48_H_88_NO_8_P | 994.6 | -0.732 | 0.88 |
| 1.99 | 306.9 | 1.509 | 894.62294 | 835.60909 | [M+CH_3_COO]^-^ | PC 18:0_22:5_A | C_48_H_86_NO_8_P | 748.1 | -0.047 | 3.0 |
| 1.91 | 306.6 | 1.508 | 894.6226 | 835.60875 | [M+CH_3_COO]^-^ | PC 18:0_22:5_B | C_48_H_86_NO_8_P | 727.7 | -0.478 | 3.0 |
| 1.83 | 305.2 | 1.501 | 892.60744 | 833.59359 | [M+CH_3_COO]^-^ | PC 18:0_22:6_A | C_48_H_84_NO_8_P | 772.1 | 0.157 | 3.1 |
| 1.85 | 298.9 | 1.468 | 834.60075 | 833.59148 | [M+H]^+^, ([M+H-H_2_O]^+^, [M+Na]^+^, [M+K]^+^) | PC 18:0_22:6_B | C_48_H_84_NO_8_P | 993.8 | 0.093 | 0.99 |
| 2.36 | 307.9 | 1.513 | 866.66258 | 865.65531 | [M+H]^+^ | PC 18:0_24:4 | C_50_H_92_NO_8_P | 797.7 | -0.842 | 0.9 |
| 2.11 | 305.8 | 1.503 | 864.64421 | 863.63694 | [M+H]^+^ | PC 18:0_24:5 | C_50_H_90_NO_8_P | 966.2 | -3.971 | 0.9 |
| 1.94 | 298.9 | 1.469 | 844.6083 | 785.59445 | [M+CH_3_COO]^-^ | PC 18:1_18:1 | C_44_H_84_NO_8_P | 796.2 | 1.197 | 2.8 |
| 1.80 | 301.7 | 1.483 | 868.60637 | 809.59251 | [M+CH_3_COO]^-^ | PC 18:1_20:3_A | C_46_H_84_NO_8_P | 789.8 | -1.166 | 2.9 |
| 1.80 | 294.9 | 1.448 | 810.59917 | 809.59168 | [M+H]^+^, ([M+H-H_2_O]^+^) | PC 18:1_20:3_B | C_46_H_84_NO_8_P | 994.7 | -1.839 | 0.4 |
| 1.70 | 293.8 | 1.443 | 808.5842 | 807.57734 | [M+H]^+^, ([M+K]^+^) | PC 18:1_20:4 | C_46_H_82_NO_8_P | 995.0 | -1.083 | 0.8 |
| 1.63 | 303.7 | 1.493 | 890.58966 | 831.57581 | [M+CH_3_COO]^-^ | PC 18:1_22:6_A | C_48_H_82_NO_8_P | 758.9 | -2.2 | 3.1 |
| 1.65 | 296.4 | 1.456 | 832.58417 | 831.57689 | [M+H]^+^ | PC 18:1_22:6_B | C_48_H_82_NO_8_P | 994.4 | -1.082 | 1.0 |
| 1.56 | 294.8 | 1.448 | 840.57425 | 781.56039 | [M+CH_3_COO]^-^ | PC 18:2_18:2 | C_44_H_80_NO_8_P | 878.7 | -2.049 | 2.6 |
| 1.54 | 291.3 | 1.43 | 806.56935 | 805.56148 | [M+H]^+^, ([M+K]^+^, [M+Na]^+^) | PC 18:2_20:4_A | C_46_H_80_NO_8_P | 993.7 | -0.033 | 0.7 |
| 1.52 | 298.6 | 1.468 | 864.57458 | 805.56072 | [M+CH_3_COO]^-^ | PC 18:2_20:4_B | C_46_H_80_NO_8_P | 836.3 | -1.665 | 2.7 |
| 1.42 | 289.3 | 1.42 | 804.55354 | 803.54626 | [M+H]^+^ | PC 18:2_20:5 | C_46_H_78_NO_8_P | 992.4 | -0.344 | 1.0 |
| 1.47 | 301.0 | 1.48 | 888.57432 | 829.56047 | [M+CH_3_COO]^-^ | PC 18:2_22:6 | C_48_H_80_NO_8_P | 850.8 | -1.903 | 3.0 |
| 1.49 | 293.8 | 1.443 | 830.56907 | 829.56179 | [M+H]^+^ | PC 20:4_20:4 | C_48_H_80_NO_8_P | 995.3 | -0.437 | 0.8 |
| 1.35 | 291.0 | 1.429 | 828.55225 | 827.54497 | [M+H]^+^ | PC 20:4_20:5 | C_48_H_78_NO_8_P | 995.4 | -1.87 | 0.6 |
| 1.23 | 271.5 | 1.329 | 676.48882 | 675.48155 | [M+H]^+^ | PC 28:1 | C_36_H_70_NO_8_P | 1000 | -2.607 | 1.7 |
| 1.53 | 277.9 | 1.361 | 692.52307 | 691.51579 | [M+H]^+^ | PC 29:0 | C_37_H_74_NO_8_P | 1000 | 0.993 | 0.8 |
| 1.63 | 280.7 | 1.375 | 706.53811 | 705.5311 | [M+H]^+^, ([M+Na]^+^) | PC 30:0 | C_38_H_76_NO_8_P | 995.0 | -0.028 | 0.9 |
| 1.75 | 283.2 | 1.387 | 720.55521 | 719.54793 | [M+H]^+^ | PC 31:0 | C_39_H_78_NO_8_P | 1000 | 2.029 | 0.4 |
| 1.56 | 280.1 | 1.372 | 718.53726 | 717.52999 | [M+H]^+^ | PC 31:1 | C_39_H_76_NO_8_P | 1000 | -1.483 | 0.1 |
| 1.95 | 288.9 | 1.417 | 748.58477 | 747.57749 | [M+H]^+^ | PC 33:0 | C_41_H_82_NO_8_P | 1000 | -0.367 | 0.2 |
| 1.78 | 286.2 | 1.403 | 746.56949 | 745.56222 | [M+H]^+^ | PC 33:1 | C_41_H_80_NO_8_P | 1000 | 0.079 | 0.1 |
| 1.60 | 282.4 | 1.384 | 744.55417 | 743.5484 | [M+H]^+^, ([M+Na]^+^) | PC 33:2 | C_41_H_78_NO_8_P | 988.0 | 0.533 | 0.01 |
| 1.46 | 280.3 | 1.374 | 742.53991 | 741.53263 | [M+H]^+^ | PC 33:3 | C_41_H_76_NO_8_P | 1000 | 2.71 | 0.3 |
| 2.18 | 295.2 | 1.448 | 776.61487 | 775.60759 | [M+H]^+^ | PC 35:0 | C_43_H_86_NO_8_P | 697.0.0 | -1.607 | 0.5 |
| 1.83 | 289.5 | 1.42 | 772.58518 | 771.5779 | [M+H]^+^ | PC 35:2 | C_43_H_82_NO_8_P | 978 | -0.071 | 0.2 |
| 1.66 | 286.8 | 1.407 | 770.56915 | 769.56188 | [M+H]^+^ | PC 35:3 | C_43_H_80_NO_8_P | 922.0 | -0.415 | 0.01 |
| 1.57 | 285.9 | 1.403 | 768.55356 | 767.54628 | [M+H]^+^ | PC 35:4 | C_43_H_78_NO_8_P | 1000 | -0.294 | 0.3 |
| 2.33 | 297.9 | 1.462 | 790.62847 | 789.6212 | [M+H]^+^ | PC 36:0 | C_44_H_88_NO_8_P | 995.7 | -4.636 | 0.8 |
| 1.79 | 293.5 | 1.44 | 796.58416 | 795.57689 | [M+H]^+^ | PC 37:4 | C_45_H_82_NO_8_P | 830.0 | -1.193 | 0.8 |
| 1.62 | 290.4 | 1.425 | 794.57007 | 793.56279 | [M+H]^+^ | PC 37:5 | C_45_H_80_NO_8_P | 754.0 | 1.008 | 0.2 |
| 2.33 | 300.1 | 1.474 | 816.64694 | 815.63964 | [M+H]^+^, ([M+Na]^+^) | PC 38:1 | C_46_H_90_NO_8_P | 663.0 | -0.887 | 0.01 |
| 1.72 | 295.5 | 1.451 | 820.58505 | 819.57777 | [M+H]^+^ | PC 39:6 | C_47_H_82_NO_8_P | 916.0 | -0.099 | 0.6 |
| 2.56 | 305.9 | 1.503 | 844.67809 | 843.67081 | [M+H]^+^ | PC 40:1 | C_48_H_94_NO_8_P | 703.0 | -1.113 | 0.01 |
| 2.39 | 304.1 | 1.494 | 842.66459 | 841.65731 | [M+H]^+^ | PC 40:2 | C_48_H_92_NO_8_P | 1000 | 1.463 | 0.3 |
| 1.92 | 299.6 | 1.472 | 836.61521 | 835.60794 | [M+H]^+^ | PC 40:5_A | C_48_H_86_NO_8_P | 992.4 | -0.967 | 0.9 |
| 2.00 | 301.4 | 1.48 | 836.61435 | 835.6063 | [M+H]^+^, ([M+Na]^+^) | PC 40:5_B | C_48_H_86_NO_8_P | 989.7 | -2.357 | 1.1 |
| 1.44 | 295.9 | 1.454 | 854.56724 | 853.55996 | [M+H]^+^ | PC 42:10 | C_50_H_80_NO_8_P | 986.4 | -2.619 | 0.7 |
| 2.61 | 310.3 | 1.525 | 870.69311 | 869.68583 | [M+H]^+^ | PC 42:2 | C_50_H_96_NO_8_P | 1000 | -1.983 | 0.8 |
| 2.39 | 307.7 | 1.513 | 868.67871 | 867.67143 | [M+H]^+^ | PC 42:3 | C_50_H_94_NO_8_P | 337.2 | -0.36 | 0.4 |
| 1.99 | 304.1 | 1.495 | 862.62866 | 861.62138 | [M+H]^+^ | PC 42:6 | C_50_H_88_NO_8_P | 1000 | -3.391 | 0.5 |
| 1.66 | 299.2 | 1.471 | 858.59952 | 857.59225 | [M+H]^+^ | PC 42:8 | C_50_H_84_NO_8_P | 1000 | -1.414 | 0.01 |
| 1.52 | 297.3 | 1.461 | 856.58281 | 855.57553 | [M+H]^+^ | PC 42:9 | C_50_H_82_NO_8_P | 995.0 | -2.758 | 0.2 |
| 2.57 | 313.4 | 1.541 | 894.69434 | 893.68706 | [M+H]^+^ | PC 44:4 | C_52_H_96_NO_8_P | 612.0 | -0.178 | 0.8 |
| 2.35 | 310.8 | 1.528 | 892.67583 | 891.66855 | [M+H]^+^ | PC 44:5 | C_52_H_94_NO_8_P | 378.3 | -3.273 | 0.2 |
| 1.97 | 304.7 | 1.498 | 878.62647 | 819.61261 | [M+CH_3_COO]^-^ | PC O-18:0_22:6 | C_48_H_86_NO_7_P | 647.1 | -1.673 | 3.0 |
| 2.27 | 305.7 | 1.503 | 882.66035 | 823.6465 | [M+CH_3_COO]^-^ | PC O-20:0_20:4 | C_48_H_90_NO_7_P | 610.5 | 1.056 | 1.6 |
| 2.07 | 308.3 | 1.516 | 906.65925 | 847.6454 | [M+CH_3_COO]^-^ | PC O-20:1_22:5 | C_50_H_90_NO_7_P | 551.7 | 0.082 | 1.5 |
| 2.47 | 314.7 | 1.549 | 936.70599 | 877.69214 | [M+CH_3_COO]^-^ | PC O-24:1_20:4 | C_52_H_96_NO_7_P | 664.4 | -0.36 | 1.5 |
| 1.82 | 285.4 | 1.398 | 718.57436 | 717.56708 | [M+H]^+^ | PC O-32:1_A | C_40_H_80_NO_7_P | 999.8 | -0.199 | 1.2 |
| 1.98 | 285.4 | 1.398 | 718.57464 | 717.56737 | [M+H]^+^ | PC O-32:1_B | C_40_H_80_NO_7_P | 997.5 | 0.202 | 1.2 |
| 1.78 | 282.5 | 1.384 | 716.55915 | 715.55188 | [M+H]^+^ | PC O-32:2 | C_40_H_78_NO_7_P | 994.5 | 0.492 | 1.2 |
| 2.04 | 291.5 | 1.429 | 746.60515 | 745.59787 | [M+H]^+^ | PC O-34:1 | C_42_H_84_NO_7_P | 995.9 | -0.951 | 1.2 |
| 2.00 | 288.7 | 1.415 | 744.5899 | 743.58432 | [M+H]^+^, ([M+Na]^+^) | PC O-34:2_A | C_42_H_82_NO_7_P | 997.7 | -0.385 | 0.9 |
| 1.86 | 288.1 | 1.413 | 744.58831 | 743.58103 | [M+H]^+^ | PC O-34:2_B | C_42_H_82_NO_7_P | 998.4 | -2.459 | 0.7 |
| 1.82 | 285 | 1.397 | 742.57438 | 741.56725 | [M+H]^+^, ([M+Na]^+^) | PC O-34:3 | C_42_H_80_NO_7_P | 998.0 | -0.215 | 0.2 |
| 1.66 | 282.4 | 1.384 | 740.55819 | 739.55092 | [M+H]^+^ | PC O-34:4 | C_42_H_78_NO_7_P | 981.8 | -0.533 | 0.5 |
| 2.10 | 291.5 | 1.429 | 758.60724 | 757.59997 | [M+H]^+^ | PC O-35:2 | C_43_H_84_NO_7_P | 993.7 | 1.88 | 1.2 |
| 1.92 | 288.0 | 1.412 | 756.59037 | 755.5831 | [M+H]^+^ | PC O-35:3 | C_43_H_82_NO_7_P | 982.5 | 0.297 | 0.2 |
| 2.47 | 300.0 | 1.472 | 776.65291 | 775.64563 | [M+H]^+^ | PC O-36:0 | C_44_H_90_NO_7_P | 999.6 | 0.213 | 1.2 |
| 2.27 | 297.1 | 1.457 | 774.63621 | 773.62894 | [M+H]^+^ | PC O-36:1 | C_44_H_88_NO_7_P | 996.2 | -1.089 | 1.4 |
| 2.24 | 294.8 | 1.446 | 772.62094 | 771.61366 | [M+H]^+^ | PC O-36:2 | C_44_H_86_NO_7_P | 999.8 | -0.438 | 1.1 |
| 2.05 | 292.3 | 1.434 | 770.606 | 769.59965 | [M+H]^+^, ([M+Na]^+^) | PC O-36:3 | C_44_H_84_NO_7_P | 999.4 | 0.175 | 0.9 |
| 1.83 | 291.2 | 1.428 | 768.58945 | 767.58218 | [M+H]^+^ | PC O-36:4 | C_44_H_82_NO_7_P | 998.3 | -1.069 | 1.2 |
| 1.67 | 288.4 | 1.414 | 766.5741 | 765.56683 | [M+H]^+^ | PC O-36:5_A | C_44_H_80_NO_7_P | 998.6 | -0.533 | 0.9 |
| 1.78 | 288.5 | 1.415 | 766.57435 | 765.56707 | [M+H]^+^ | PC O-36:5_B | C_44_H_80_NO_7_P | 997.2 | -0.236 | 0.9 |
| 1.63 | 286.2 | 1.404 | 764.55919 | 763.55192 | [M+H]^+^ | PC O-36:6 | C_44_H_78_NO_7_P | 941.7 | 0.422 | 0.4 |
| 1.87 | 292.5 | 1.435 | 780.59108 | 779.58381 | [M+H]^+^ | PC O-37:5 | C_45_H_82_NO_7_P | 962.9 | 1.198 | 1.1 |
| 2.05 | 297.5 | 1.46 | 796.62103 | 795.61376 | [M+H]^+^, ([M+Na]^+^) | PC O-38:4 | C_46_H_86_NO_7_P | 997.6 | -0.572 | 1.6 |
| 2.01 | 294.7 | 1.446 | 794.6068 | 793.59952 | [M+H]^+^ | PC O-38:5 | C_46_H_84_NO_7_P | 998.1 | 1.27 | 1.0 |
| 1.78 | 300.5 | 1.476 | 850.59548 | 791.58163 | [M+CH_3_COO]^-^ | PC O-38:6 | C_46_H_82_NO_7_P | 916.0 | -1.442 | 2.8 |
| 1.71 | 292.3 | 1.434 | 790.57497 | 789.56769 | [M+H]^+^ | PC O-38:7 | C_46_H_80_NO_7_P | 693.3 | 0.585 | 1.0 |
| 2.53 | 305.8 | 1.502 | 828.6843 | 827.67702 | [M+H]^+^ | PC O-40:2 | C_48_H_94_NO_7_P | 999.2 | 0.204 | 0.6 |
| 2.28 | 302.7 | 1.486 | 824.65256 | 823.6451 | [M+H]^+^, ([M+Na]^+^) | PC O-40:4 | C_48_H_90_NO_7_P | 997.2 | -0.259 | 1.7 |
| 2.06 | 301.1 | 1.479 | 822.63594 | 821.62867 | [M+H]^+^ | PC O-40:5 | C_48_H_88_NO_7_P | 999.8 | -1.442 | 1.5 |
| 1.99 | 300.2 | 1.474 | 820.62021 | 819.61294 | [M+H]^+^ | PC O-40:6 | C_48_H_86_NO_7_P | 997.6 | -1.281 | 1.4 |
| 1.94 | 298.7 | 1.467 | 818.60582 | 817.59854 | [M+H]^+^ | PC O-40:7_A | C_48_H_84_NO_7_P | 960.5 | 0.174 | 1.9 |
| 1.77 | 297.5 | 1.461 | 818.60455 | 817.59727 | [M+H]^+^ | PC O-40:7_B | C_48_H_84_NO_7_P | 996.6 | -1.527 | 1.5 |
| 2.01 | 298.7 | 1.467 | 816.58572 | 815.57844 | [M+H]^+^ | PC O-40:8 | C_48_H_82_NO_7_P | 331.9 | -5.175 | 2.4 |
| 1.80 | 294.9 | 1.448 | 814.57131 | 813.56403 | [M+H]^+^ | PC O-40:9 | C_48_H_80_NO_7_P | 999.8 | -3.675 | 1.4 |
| 2.69 | 311.9 | 1.533 | 856.71405 | 855.70677 | [M+H]^+^ | PC O-42:2 | C_50_H_98_NO_7_P | 999.2 | -1.506 | 1.3 |
| 2.49 | 309.0 | 1.518 | 852.68438 | 851.67577 | [M+H]^+^, ([M+Na]^+^) | PC O-42:4 | C_50_H_94_NO_7_P | 999.8 | 0.367 | 1.2 |
| 2.29 | 306.1 | 1.504 | 850.66843 | 849.6594 | [M+H]^+^, ([M+Na]^+^) | PC O-42:5 | C_50_H_92_NO_7_P | 999.8 | 0.025 | 0.6 |
| 2.10 | 304.6 | 1.496 | 848.65167 | 847.64339 | [M+H]^+^, ([M+K]^+^) | PC O-42:6 | C_50_H_90_NO_7_P | 996.1 | -1.477 | 0.7 |
| 1.99 | 303.3 | 1.49 | 846.6362 | 845.62893 | [M+H]^+^ | PC O-42:7 | C_50_H_88_NO_7_P | 931.6 | -1.076 | 1.3 |
| 2.68 | 314.4 | 1.546 | 880.71684 | 879.70957 | [M+H]^+^ | PC O-44:4 | C_52_H_98_NO_7_P | 996.3 | 1.69 | 0.8 |
| 2.49 | 312.0 | 1.534 | 878.69905 | 877.69165 | [M+H]^+^, ([M+Na]^+^) | PC O-44:5 | C_52_H_96_NO_7_P | 997.5 | -0.76 | 0.7 |
| 2.31 | 310.3 | 1.525 | 876.68284 | 875.67557 | [M+H]^+^ | PC O-44:6 | C_52_H_94_NO_7_P | 999.1 | -1.433 | 0.7 |
| 2.21 | 308.7 | 1.517 | 874.66723 | 873.65996 | [M+H]^+^ | PC O-44:7 | C_52_H_92_NO_7_P | 999.8 | -1.352 | 1.2 |
| 2.11 | 305.7 | 1.503 | 870.6318 | 869.62452 | [M+H]^+^ | PC O-44:9 | C_52_H_88_NO_7_P | 294.6 | -5.902 | 1.4 |
| 2.66 | 317.2 | 1.56 | 906.72964 | 905.72237 | [M+H]^+^ | PC O-46:5 | C_54_H_100_NO_7_P | 986.5 | -1.58 | 0.5 |
| 2.50 | 315.5 | 1.552 | 904.71084 | 903.70357 | [M+H]^+^ | PC O-46:6 | C_54_H_98_NO_7_P | 995.0 | -4.841 | 0.7 |
| 2.31 | 313.2 | 1.54 | 902.69621 | 901.68893 | [M+H]^+^ | PC O-46:7_A | C_54_H_96_NO_7_P | 651.4 | -3.82 | 0.6 |
| 2.42 | 314.3 | 1.546 | 902.69859 | 901.69131 | [M+H]^+^ | PC O-46:7_B | C_54_H_96_NO_7_P | 992.3 | -1.235 | 0.9 |
| 2.24 | 311.6 | 1.533 | 900.68238 | 899.67511 | [M+H]^+^ | PC O-46:8 | C_54_H_94_NO_7_P | 988.7 | -1.872 | 0.5 |
| 1.78 | 274.2 | 1.343 | 716.52298 | 715.51571 | [M+H]^+^ | PE 16:0_18:2 | C_39_H_74_NO_8_P | 801.6 | 0.611 | 1.9 |
| 1.73 | 270.3 | 1.325 | 738.50806 | 739.51534 | [M-H]^-^ | PE 16:0_20:4 | C_41_H_74_NO_8_P | 785.6 | 0.197 | 1.3 |
| 1.68 | 280.1 | 1.374 | 764.52233 | 763.51505 | [M+H]^+^ | PE 16:1_22:5 | C_43_H_74_NO_8_P | 910.6 | -0.219 | 1.1 |
| 1.96 | 276.7 | 1.357 | 766.53919 | 767.54646 | [M-H]^-^ | PE 18:0_20:4_A | C_43_H_78_NO_8_P | 798.3 | -0.033 | 1.3 |
| 1.97 | 284.4 | 1.395 | 768.55348 | 767.5462 | [M+H]^+^ | PE 18:0_20:4_B | C_43_H_78_NO_8_P | 926.0 | -0.355 | 1.3 |
| 2.19 | 283.7 | 1.391 | 746.56808 | 745.56156 | [M+H]^+^, ([M+Na]^+^) | PE 18:1_18:0 _B | C_41_H_80_NO_8_P | 932.0 | -1.691 | 0.8 |
| 2.17 | 274.6 | 1.346 | 744.55502 | 745.56229 | [M-H]^-^ | PE 18:1_18:0_A | C_41_H_80_NO_8_P | 854.7 | 0.077 | 1.9 |
| 1.80 | 277.1 | 1.358 | 742.53776 | 741.53048 | [M+H]^+^ | PE 18:1_18:2 | C_41_H_76_NO_8_P | 721.0 | -0.528 | 1.6 |
| 1.77 | 280.5 | 1.376 | 766.53869 | 765.53141 | [M+H]^+^ | PE 18:1_20:4_A | C_43_H_76_NO_8_P | 836.9 | 0.574 | 1.1 |
| 1.75 | 274.8 | 1.348 | 764.52424 | 765.53152 | [M-H]^-^ | PE 18:1_20:4_B | C_43_H_76_NO_8_P | 477.6 | 0.834 | 1.3 |
| 1.68 | 278.7 | 1.368 | 788.5229 | 789.53017 | [M-H]^-^ | PE 18:1_22:6 | C_45_H_76_NO_8_P | 689.5 | -0.473 | 1.5 |
| 1.76 | 266.4 | 1.305 | 714.50813 | 715.51541 | [M-H]^-^ | PE 18:2_16:0 | C_39_H_74_NO_8_P | 871.1 | 0.273 | 1.5 |
| 1.99 | 272.9 | 1.338 | 742.53932 | 743.5466 | [M-H]^-^ | PE 18:2_18:0 | C_41_H_78_NO_8_P | 750.6 | 0.137 | 1.6 |
| 1.79 | 270.8 | 1.327 | 740.5228 | 741.53008 | [M-H]^-^ | PE 18:2_18:1 | C_41_H_76_NO_8_P | 973.8 | -1.057 | 1.2 |
| 1.66 | 274.7 | 1.347 | 762.50815 | 763.51542 | [M-H]^-^ | PE 22:6_16:0 | C_43_H_74_NO_8_P | 626.3 | 0.269 | 1.4 |
| 1.96 | 276.9 | 1.357 | 718.53823 | 717.53095 | [M+H]^+^ | PE 34:1 | C_39_H_76_NO_8_P | 1000 | -0.234 | 0.9 |
| 2.01 | 280.9 | 1.377 | 744.55396 | 743.54595 | [M+H]^+^, ([M+Na]^+^) | PE 36:2 | C_41_H_78_NO_8_P | 941.0 | 0.315 | 1.8 |
| 1.73 | 277.9 | 1.362 | 740.52252 | 739.51524 | [M+H]^+^ | PE 36:4 | C_41_H_74_NO_8_P | 874.9 | 0.152 | 1.2 |
| 2.07 | 286.6 | 1.406 | 770.56979 | 769.56251 | [M+H]^+^ | PE 38:3 | C_43_H_80_NO_8_P | 855.0 | 0.332 | 0.7 |
| 1.91 | 286.2 | 1.405 | 792.55465 | 791.54738 | [M+H]^+^ | PE 40:6 | C_45_H_78_NO_8_P | 1000 | 0.535 | 0.6 |
| 1.58 | 290.4 | 1.425 | 790.53791 | 789.53063 | [M+H]^+^ | PE 40:7 | C_45_H_76_NO_8_P | 711.0 | -0.5 | 2.3 |
| 2.08 | 275.6 | 1.35 | 702.54329 | 701.53601 | [M+H]^+^ | PE P-16:0_18:1_A | C_39_H_76_NO_7_P | 928.3 | 0.145 | 1.9 |
| 2.06 | 267.6 | 1.31 | 700.52839 | 701.53567 | [M-H]^-^ | PE P-16:0_18:1_B | C_39_H_76_NO_7_P | 935.0 | -0.297 | 0.18 |
| 1.87 | 266.0 | 1.302 | 698.5125 | 699.51978 | [M-H]^-^ | PE P-16:0_18:2 | C_39_H_74_NO_7_P | 985.0 | -0.745 | 1.4 |
| 1.85 | 275.1 | 1.348 | 724.52764 | 723.52036 | [M+H]^+^ | PE P-16:0_20:4_A | C_41_H_74_NO_7_P | 934.1 | 0.108 | 2.1 |
| 1.83 | 270.2 | 1.324 | 722.51298 | 723.52026 | [M-H]^-^ | PE P-16:0_20:4_B | C_41_H_74_NO_79_ | 983.2 | -0.028 | 1.7 |
| 1.67 | 269.0 | 1.318 | 720.4975 | 721.50477 | [M-H]^-^ | PE P-16:0_20:5 | C_41_H_72_NO_7_P | 972.0 | 0.186 | 0.19 |
| 1.87 | 278.9 | 1.368 | 750.54271 | 749.53543 | [M+H]^+^ | PE P-16:0_22:5_A | C_43_H_76_NO_7_P | 813.1 | -0.656 | 0.2 |
| 1.85 | 274.5 | 1.346 | 748.52764 | 749.53492 | [M-H]^-^ | PE P-16:0_22:5_B | C_43_H_76_NO_7_P | 862.0 | -1.222 | 0.25 |
| 1.78 | 278.2 | 1.364 | 748.52761 | 747.52033 | [M+H]^+^ | PE P-16:0_22:6_A | C_43_H_74_NO_7_P | 944.9 | -0.049 | 2.0 |
| 1.76 | 273.7 | 1.342 | 746.51307 | 747.52035 | [M-H]^-^ | PE P-16:0_22:6_B | C_43_H_74_NO_7_P | 966.0 | 0.333 | 1.6 |
| 2.31 | 282.2 | 1.383 | 730.57365 | 729.56638 | [M+H]^+^ | PE P-18:0_18:1_A | C_41_H_80_NO_7_P | 921.6 | -1.088 | 0.27 |
| 2.28 | 273.9 | 1.342 | 728.56131 | 729.56858 | [M-H]^-^ | PE P-18:0_18:1_B | C_41_H_80_NO_7_P | 945.0 | 1.664 | 2.0 |
| 2.12 | 278.8 | 1.366 | 728.55898 | 727.55183 | [M+H]^+^, ([M+Na]^+^) | PE P-18:0_18:2_A | C_41_H_78_NO_7_P | 930.4 | 0.061 | 1.7 |
| 2.10 | 272.2 | 1.334 | 726.54412 | 727.55139 | [M-H]^-^ | PE P-18:0_18:2_B | C_41_H_78_NO_7_P | 867.0 | -0.287 | 1.7 |
| 2.08 | 282.7 | 1.386 | 752.5586 | 751.55142 | [M+H]^+^, ([M+Na]^+^) | PE P-18:0_20:4 | C_43_H_78_NO_7_P | 957.9 | -0.353 | 1.6 |
| 2.30 | 288.7 | 1.416 | 780.58699 | 779.57972 | [M+H]^+^ | PE P-18:0_22:4_A | C_45_H_82_NO_7_P | 914.8 | -5.045 | 0.8 |
| 2.26 | 281.9 | 1.383 | 778.57572 | 779.583 | [M-H]^-^ | PE P-18:0_22:4_B | C_45_H_82_NO_7_P | 968.0 | 0.215 | 0.16 |
| 2.07 | 280.7 | 1.377 | 776.55919 | 777.56647 | [M-H]^-^ | PE P-18:0_22:5 | C_45_H_80_NO_7_P | 925.0 | -1.001 | 1.6 |
| 1.99 | 279.8 | 1.373 | 774.54431 | 775.55159 | [M-H]^-^ | PE P-18:0_22:6 | C_45_H_78_NO_7_P | 896.0 | 0.013 | 1.9 |
| 1.79 | 278.2 | 1.365 | 772.52881 | 773.53609 | [M-H]^-^ | PE P-18:1_22:6 | C_45_H_76_NO_7_P | 975.0 | 0.194 | 0.02 |
| 2.33 | 278.5 | 1.366 | 754.57518 | 755.58246 | [M-H]^-^ | PE P-20:0_18:2 | C_43_H_82_NO_7_P | 965.0 | -0.411 | 1.9 |
| 1.34 | 283.5 | 1.392 | 807.50307 | 808.51035 | [M-H]^-^ | PI 16:0_16:1 | C_41_H_77_O_13_P | 698.4 | 0.228 | 2.7 |
| 1.39 | 287.7 | 1.413 | 833.51839 | 834.52566 | [M-H]^-^ | PI 16:0_18:2 | C_43_H_79_O_13_P | 658.9 | -0.13 | 2.0 |
| 1.36 | 291.3 | 1.432 | 857.51867 | 858.52594 | [M-H]^-^ | PI 16:0_20:4 | C_45_H_79_O_13_P | 607.4 | 0.08 | 2.6 |
| 1.31 | 294.7 | 1.449 | 881.51854 | 882.52582 | [M-H]^-^ | PI 16:0_22:6 | C_47_H_79_O_13_P | 567.6 | -0.002 | 2.9 |
| 1.75 | 295.9 | 1.454 | 863.56687 | 864.57415 | [M-H]^-^ | PI 18:0_18:1 | C_45_H_85_O_13_P | 662.3 | 1.619 | 2.9 |
| 1.59 | 293.9 | 1.445 | 861.54995 | 862.55723 | [M-H]^-^ | PI 18:0_18:2 | C_45_H_83_O_13_P | 687.1 | 0.083 | 2.9 |
| 1.65 | 298.6 | 1.468 | 887.56535 | 888.57263 | [M-H]^-^ | PI 18:0_20:3 | C_47_H_85_O_13_P | 529.3 | -0.188 | 2.5 |
| 1.56 | 297.6 | 1.463 | 885.55054 | 886.55781 | [M-H]^-^ | PI 18:0_20:4 | C_47_H_83_O_13_P | 658.0 | 0.791 | 3.0 |
| 1.51 | 300.7 | 1.479 | 909.54897 | 910.55625 | [M-H]^-^ | PI 18:0_22:6 | C_49_H_83_O_13_P | 619.4 | -0.959 | 2.8 |
| 1.54 | 288.7 | 1.418 | 835.53436 | 836.54163 | [M-H]^-^ | PI 18:1_16:0 | C_43_H_81_O_13_P | 588.0 | 0.198 | 1.7 |
| 1.42 | 292.2 | 1.436 | 859.53318 | 860.54046 | [M-H]^-^ | PI 18:1_18:2 | C_45_H_81_O_13_P | 508.7 | -1.177 | 2.6 |
| 1.38 | 295.6 | 1.453 | 883.5337 | 884.54098 | [M-H]^-^ | PI 18:1_20:4 | C_47_H_81_O_13_P | 420.2 | -0.53 | 2.9 |
| 1.47 | 296.1 | 1.455 | 852.55779 | 834.52472 | [M+NH_4_]^+^, ([M+Na]^+^, [M+H]^+^) | PI 34:2 | C_43_H_79_O_13_P | 931.6 | -2.373 | 2.5 |
| 1.85 | 304.8 | 1.498 | 882.60539 | 864.57157 | [M+NH_4_]^+^ | PI 36:1 | C_45_H_85_O_13_P | 934.8 | -1.274 | 3.4 |
| 1.68 | 301.3 | 1.481 | 880.59067 | 862.55673 | [M+NH_4_]^+^, ([M+H]^+^) | PI 36:2 | C_45_H_83_O_13_P | 859.3 | -0.327 | 2.7 |
| 1.51 | 299.7 | 1.473 | 878.57506 | 860.54123 | [M+NH_4_]^+^ | PI 36:3 | C_45_H_81_O_13_P | 924.2 | -0.192 | 2.5 |
| 1.44 | 299.6 | 1.473 | 876.56112 | 858.52023 | [M+NH_4_]^+^, ([M+Na]^+^, [M+H]^+^) | PI 36:4 | C_45_H_79_O_13_P | 934.4 | 1.672 | 3.0 |
| 1.46 | 294.3 | 1.446 | 871.53166 | 872.53893 | [M-H]^-^ | PI 37:4 | C_46_H_81_O_13_P | 368.0 | -2.859 | 3.0 |
| 1.74 | 305.8 | 1.504 | 906.60646 | 888.57263 | [M+NH_4_]^+^ | PI 38:3 | C_47_H_85_O_13_P | 1000 | -0.201 | 0.4 |
| 1.66 | 305.1 | 1.501 | 904.59077 | 886.55664 | [M+NH_4_]^+^, ([M+H]^+^, [M+Na]^+^) | PI 38:4 | C_47_H_83_O_13_P | 943.3 | -0.226 | 3.4 |
| 1.47 | 302.6 | 1.488 | 902.57575 | 884.54037 | [M+NH_4_]^+^, ([M+H]^+^) | PI 38:5 | C_47_H_81_O_13_P | 946.1 | 0.325 | 3.0 |
| 1.39 | 302.3 | 1.487 | 900.56267 | 882.52884 | [M+NH_4_]^+^ | PI 38:6 | C_47_H_79_O_13_P | 928.4 | 3.223 | 3.3 |
| 1.66 | 308.3 | 1.517 | 930.60538 | 912.57155 | [M+NH_4_]^+^ | PI 40:5 | C_49_H_85_O_13_P | 966.2 | -1.312 | 2.9 |
| 1.60 | 307.6 | 1.514 | 928.58993 | 910.55611 | [M+NH_4_]^+^ | PI 40:6 | C_49_H_83_O_13_P | 956.7 | -1 | 2.9 |
| 1.81 | 283.9 | 1.393 | 788.54482 | 789.5521 | [M-H]^-^ | PS 18:0_18:1 | C_42_H_80_NO_10_P | 658.7 | 0.059 | 1.4 |
| 1.60 | 285.5 | 1.402 | 810.52933 | 811.53661 | [M-H]^-^ | PS 18:0_20:4 | C_44_H_78_NO_10_P | 683.6 | 0.4 | 1.7 |
| 1.79 | 299.6 | 1.472 | 854.59007 | 855.59735 | [M-H]^-^ | PS 21:0_20:3 | C_47_H_86_NO_10_P | 185.0 | -1.844 | 4.1 |
| 1.39 | 281.5 | 1.378 | 675.54446 | 674.53759 | [M+H]^+^, ([M+K]^+^, [M+Na]^+^) | SM 16:1;2O/16:0 | C_37_H_75_N_2_O_6_P | 999.5 | 1.331 | 1.4 |
| 1.62 | 286.2 | 1.402 | 703.57578 | 702.56851 | [M+H]^+^ | SM 18:1;2O/16:0 | C_39_H_79_N_2_O_6_P | 998.9 | 1.328 | 0.3 |
| 1.86 | 292.5 | 1.434 | 731.60623 | 730.59923 | [M+H]^+^, ([M+Na]^+^) | SM 18:1;2O/18:0 | C_41_H_83_N_2_O_6_P | 998.9 | 0.114 | 0.6 |
| 2.10 | 298.3 | 1.463 | 759.63762 | 758.63043 | [M+H]^+^, ([M+K]^+^, [M+Na]^+^) | SM 18:1;2O/20:0 | C_43_H_87_N_2_O_6_P | 999.0 | 0.267 | 1.0 |
| 2.44 | 305.6 | 1.5 | 801.68445 | 800.67655 | [M+H]^+^, ([M+K]^+^, [M+Na]^+^) | SM 18:1;2O/23:0 | C_46_H_93_N_2_O_6_P | 998.5 | 0.072 | 0.7 |
| 2.55 | 308.7 | 1.516 | 815.70027 | 814.69216 | [M+H]^+^, ([M+K]^+^, [M+Na]^+^) | SM 18:1;2O/24:0 | C_47_H_95_N_2_O_6_P | 999.0 | 0.291 | 0.8 |
| 2.32 | 306.3 | 1.504 | 813.68473 | 812.67745 | [M+H]^+^ | SM 18:1;2O/24:1 | C_47_H_93_N_2_O_6_P | 999.1 | 0.426 | 1.0 |
| 1.65 | 288.3 | 1.413 | 729.59038 | 728.5832 | [M+H]^+^, ([M+Na]^+^) | SM 18:2;2O/18:0 | C_41_H_81_N_2_O_6_P | 999.7 | -0.115 | 0.2 |
| 1.16 | 275.5 | 1.347 | 647.51122 | 646.50395 | [M+H]^+^ | SM 30:1;2O | C_35_H_71_N_2_O_6_P | 989.0 | -1.883 | 2.7 |
| 1.27 | 278.2 | 1.361 | 661.52833 | 660.52105 | [M+H]^+^ | SM 31:1;2O | C_36_H_73_N_2_O_6_P | 960.0 | 0.657 | 2.3 |
| 1.47 | 285.0 | 1.395 | 677.56012 | 676.55284 | [M+H]^+^ | SM 32:0;2O | C_37_H_77_N_2_O_6_P | 907.0 | 1.316 | 2.2 |
| 1.20 | 277.0 | 1.355 | 673.52681 | 672.52153 | [M+H]^+^, ([M+Na]^+^) | SM 32:2;2O | C_37_H_73_N_2_O_6_P | 948.0 | -1.414 | 0.7 |
| 1.50 | 284.3 | 1.392 | 689.55974 | 688.55266 | [M+H]^+^, ([M+Na]^+^) | SM 33:1;2O | C_38_H_77_N_2_O_6_P | 1000 | 0.785 | 1.1 |
| 1.32 | 279.8 | 1.37 | 687.54337 | 686.53609 | [M+H]^+^ | SM 33:2;2O | C_38_H_75_N_2_O_6_P | 975.0 | -0.597 | 0.2 |
| 1.70 | 290.3 | 1.422 | 705.59115 | 704.58388 | [M+H]^+^ | SM 34:0;2O | C_39_H_81_N_2_O_6_P | 955.0 | 0.921 | 1.2 |
| 1.51 | 291.7 | 1.429 | 721.5839 | 720.57662 | [M+H]^+^ | SM 34:0;3O | C_39_H_81_N_2_O_7_P | 858.8 | -1.89 | 2.1 |
| 1.29 | 288.7 | 1.415 | 719.5696 | 718.56232 | [M+H]^+^ | SM 34:1;3O | C_39_H_79_N_2_O_7_P | 888.2 | -0.093 | 1.3 |
| 1.42 | 283.5 | 1.388 | 701.56015 | 700.55295 | [M+H]^+^, ([M+K]^+^, [M+Na]^+^) | SM 34:2;2O | C_39_H_77_N_2_O_6_P | 970.0 | 1.358 | 0.6 |
| 1.35 | 289.2 | 1.417 | 717.55539 | 716.54812 | [M+H]^+^ | SM 34:2;3O | C_39_H_77_N_2_O_7_P | 917.1 | 1.502 | 2.1 |
| 1.26 | 280.1 | 1.371 | 699.54199 | 698.53471 | [M+H]^+^ | SM 34:3;2O | C_39_H_75_N_2_O_6_P | 1000 | -2.419 | 0.4 |
| 1.73 | 290.0 | 1.421 | 717.59075 | 716.58347 | [M+H]^+^ | SM 35:1;2O | C_40_H_81_N_2_O_6_P | 968.0 | 0.343 | 0.3 |
| 1.54 | 285.4 | 1.398 | 715.57406 | 714.56697 | [M+H]^+^, ([M+Na]^+^) | SM 35:2;2O | C_40_H_79_N_2_O_6_P | 911.0 | -1.066 | 0.01 |
| 1.94 | 295.8 | 1.45 | 733.62316 | 732.61589 | [M+H]^+^ | SM 36:0;2O | C_41_H_85_N_2_O_6_P | 1000 | 1.866 | 1.2 |
| 1.52 | 295.5 | 1.449 | 747.60012 | 746.59285 | [M+H]^+^ | SM 36:1;3O | C_41_H_83_N_2_O_7_P | 882.7 | -1.308 | 1.9 |
| 1.47 | 287.0 | 1.406 | 727.57404 | 726.56676 | [M+H]^+^ | SM 36:3;2O | C_41_H_79_N_2_O_6_P | 958.0 | -1.163 | 0.01 |
| 1.62 | 287.7 | 1.41 | 725.55679 | 724.54951 | [M+H]^+^ | SM 36:4;2O | C_41_H_77_N_2_O_6_P | 970.8 | -3.33 | 1.6 |
| 1.98 | 295.6 | 1.449 | 745.62183 | 744.61456 | [M+H]^+^ | SM 37:1;2O | C_42_H_85_N_2_O_6_P | 1000 | 0.064 | 0.8 |
| 2.18 | 300.2 | 1.472 | 761.65446 | 760.64718 | [M+H]^+^ | SM 38:0;2O | C_43_H_89_N_2_O_6_P | 981.0 | 1.745 | 0.3 |
| 1.89 | 295.1 | 1.447 | 757.62111 | 756.61384 | [M+H]^+^ | SM 38:2;2O | C_43_H_85_N_2_O_6_P | 889.0 | -0.914 | 0.3 |
| 2.22 | 299.9 | 1.471 | 773.6528 | 772.64439 | [M+H]^+^, ([M+Na]^+^) | SM 39:1;2O | C_44_H_89_N_2_O_6_P | 968.0 | -0.396 | 0.01 |
| 2.01 | 297.9 | 1.461 | 771.63746 | 770.63019 | [M+H]^+^ | SM 39:2;2O | C_44_H_87_N_2_O_6_P | 892.0 | 0.002 | 0.01 |
| 2.22 | 306.2 | 1.503 | 805.67793 | 804.67065 | [M+H]^+^ | SM 40:0;3O | C_45_H_93_N_2_O_7_P | 894.2 | -1.654 | 1.1 |
| 2.33 | 302.4 | 1.484 | 787.66903 | 786.66149 | [M+H]^+^, ([M+K]^+^, [M+Na]^+^) | SM 40:1;2O | C_45_H_91_N_2_O_6_P | 934.0 | 0.407 | 0.01 |
| 1.98 | 304.4 | 1.494 | 803.66214 | 802.65487 | [M+H]^+^ | SM 40:1;3O | C_45_H_91_N_2_O_7_P | 841.5 | -1.844 | 1.5 |
| 2.13 | 300.7 | 1.476 | 785.65293 | 784.64556 | [M+H]^+^, ([M+Na]^+^, [M+K]^+^) | SM 40:2;2O | C_45_H_89_N_2_O_6_P | 945.0 | -0.154 | 0.01 |
| 1.54 | 300.1 | 1.473 | 801.64483 | 800.63755 | [M+H]^+^ | SM 40:2;3O | C_45_H_89_N_2_O_7_P | 924.7 | -3.301 | 1.1 |
| 1.90 | 298.2 | 1.463 | 783.6363 | 782.62903 | [M+H]^+^ | SM 40:3;2O | C_45_H_87_N_2_O_6_P | 478.0 | -1.436 | 0.5 |
| 2.24 | 302.6 | 1.485 | 799.6682 | 798.66067 | [M+H]^+^, ([M+Na]^+^) | SM 41:2;2O | C_46_H_91_N_2_O_6_P | 942.0 | -0.592 | 0.1 |
| 2.02 | 301.2 | 1.478 | 797.65296 | 796.64568 | [M+H]^+^ | SM 41:3;2O | C_46_H_89_N_2_O_6_P | 876.0 | -0.3 | 0.3 |
| 2.46 | 312.7 | 1.536 | 831.69553 | 830.68825 | [M+H]^+^ | SM 42:1;3O_A | C_47_H_95_N_2_O_7_P | 707.0 | 1.096 | 2.0 |
| 2.21 | 308.8 | 1.517 | 831.69404 | 830.68676 | [M+H]^+^ | SM 42:1;3O_B | C_47_H_95_N_2_O_7_P | 901.1 | -1.055 | 0.7 |
| 1.99 | 307.8 | 1.512 | 829.67851 | 828.67124 | [M+H]^+^ | SM 42:2;3O | C_47_H_93_N_2_O_7_P | 725.6 | -1.008 | 0.9 |
| 2.12 | 303.6 | 1.49 | 811.66865 | 810.66149 | [M+H]^+^, ([M+K]^+^, [M+Na]^+^) | SM 42:3;2O | C_47_H_91_N_2_O_6_P | 935.0 | 0.005 | 0.7 |
| 2.05 | 306.5 | 1.505 | 827.66394 | 826.65666 | [M+H]^+^ | SM 42:3;3O | C_47_H_91_N_2_O_7_P | 894.2 | 0.439 | 1.6 |
| 1.79 | 299.2 | 1.469 | 807.63476 | 806.62748 | [M+H]^+^ | SM 42:5;2O | C_47_H_87_N_2_O_6_P | 719.0 | -3.544 | 0.01 |
| 2.64 | 310.6 | 1.525 | 829.71537 | 828.70877 | [M+H]^+^, ([M+Na]^+^) | SM 43:1;2O | C_48_H_97_N_2_O_6_P | 980.0 | -0.435 | 0.6 |
| 2.40 | 308.2 | 1.514 | 827.6996 | 826.69153 | [M+H]^+^, ([M+Na]^+^) | SM 43:2;2O | C_48_H_95_N_2_O_6_P | 924.0 | -0.512 | 0.01 |
| 2.24 | 305.8 | 1.502 | 825.68417 | 824.67689 | [M+H]^+^ | SM 43:3;2O | C_48_H_93_N_2_O_6_P | 881.0 | -0.274 | 0.01 |
| 2.74 | 313.4 | 1.54 | 843.732 | 842.72472 | [M+H]^+^ | SM 44:1;2O | C_49_H_99_N_2_O_6_P | 1000 | 0.767 | 0.6 |
| 2.54 | 312.4 | 1.535 | 841.71463 | 840.70735 | [M+H]^+^ | SM 44:2;2O | C_49_H_97_N_2_O_6_P | 909.0 | -1.22 | 0.9 |
| 2.35 | 309.4 | 1.52 | 839.69588 | 838.6886 | [M+H]^+^ | SM 44:3;2O | C_49_H_95_N_2_O_6_P | 1000 | -5.042 | 0.4 |
| 2.16 | 306.9 | 1.508 | 837.68013 | 836.67285 | [M+H]^+^ | SM 44:4;2O | C_49_H_93_N_2_O_6_P | 876.0 | -5.041 | 0.3 |
| 2.32 | 306.0 | 1.503 | 835.66567 | 834.65839 | [M+H]^+^ | SM 44:5;2O | C_49_H_91_N_2_O_6_P | 1000 | -3.774 | 0.1 |
| 2.66 | 278.1 | 1.36 | 656.58249 | 638.54825 | [M+NH_4_]^+^, ([M+K]^+^) | TG 12:0_12:0_12:0 | C_39_H_74_O_6_ | 979.4 | 0.099 | 4.0 |
| 2.82 | 283.7 | 1.389 | 684.6139 | 666.57969 | [M+NH_4_]^+^, ([M+K]^+^) | TG 12:0_12:0_14:0 | C_41_H_78_O_6_ | 973.6 | 0.319 | 3.1 |
| 2.93 | 289.9 | 1.42 | 712.64399 | 694.60943 | [M+NH_4_]^+^, ([M+Na]^+^, [M+K]^+^) | TG 12:0_12:0_16:0 | C_43_H_82_O_6_ | 949.2 | -1.331 | 2.8 |
| 2.98 | 305.0 | 1.498 | 816.70573 | 798.67294 | [M+NH_4_]^+^, ([M+K]^+^, [M+Na]^+^) | TG 12:0_18:2_18:2 | C_51_H_90_O_6_ | 958.0 | -2.334 | 2.6 |
| 3.12 | 313.6 | 1.541 | 846.75418 | 828.72079 | [M+NH_4_]^+^, ([M+K]^+^, [M+Na]^+^) | TG 14:0_18:1_18:2 | C_53_H_96_O_6_ | 723.8 | -0.365 | 0.3 |
| 3.06 | 311.7 | 1.532 | 844.73828 | 826.70436 | [M+NH_4_]^+^, ([M+Na]^+^, [M+K]^+^) | TG 14:0_18:2_18:2 | C_53_H_94_O_6_ | 897.0 | -0.709 | 2.7 |
| 3.00 | 309.5 | 1.52 | 842.71933 | 824.6855 | [M+NH_4_]^+^ | TG 14:0_18:2_18:3 | C_53_H_92_O_6_ | 768.1 | -4.333 | 0.1 |
| 3.15 | 316.9 | 1.557 | 860.76895 | 842.73519 | [M+NH_4_]^+^, ([M+Na]^+^, [M+K]^+^) | TG 15:0_18:1_18:2 | C_54_H_98_O_6_ | 914.3 | -1.415 | 2.1 |
| 3.23 | 317.7 | 1.561 | 850.78473 | 832.7504 | [M+NH_4_]^+^, ([M+Na]^+^, [M+K]^+^) | TG 16:0_16:0_18:1 | C_53_H_100_O_6_ | 956.0 | -1.212 | 1.8 |
| 3.18 | 315.6 | 1.551 | 848.76966 | 830.73584 | [M+NH_4_]^+^ | TG 16:0_16:1_18:1 | C_53_H_98_O_6_ | 898.5 | -0.568 | 2.1 |
| 3.28 | 323.9 | 1.592 | 878.8173 | 860.78347 | [M+NH_4_]^+^ | TG 16:0_18:0_18:1 | C_55_H_104_O_6_ | 615.1 | 0.234 | 2.0 |
| 3.23 | 321.7 | 1.581 | 876.80074 | 858.76648 | [M+NH_4_]^+^, ([M+K]^+^, [M+Na]^+^) | TG 16:0_18:1_18:1 | C_55_H_102_O_6_ | 961.4 | -0.811 | 2.5 |
| 3.18 | 319.5 | 1.57 | 874.78535 | 856.75114 | [M+NH_4_]^+^, ([M+Na]^+^ ) | TG 16:0_18:1_18:2 | C_55_H_100_O_6_ | 943.5 | -0.532 | 2.4 |
| 3.13 | 317.4 | 1.56 | 872.76976 | 854.73547 | [M+NH_4_]^+^, ([M+Na]^+^, [M+K]^+^) | TG 16:0_18:2_18:2 | C_55_H_98_O_6_ | 904.9 | -0.447 | 2.8 |
| 2.97 | 316.4 | 1.556 | 892.73611 | 874.70222 | [M+NH_4_]^+^, ([M+Na]^+^, [M+K]^+^) | TG 16:0_18:3_20:5 | C_57_H_94_O_6_ | 455.7 | -2.927 | 0.9 |
| 2.96 | 323.6 | 1.593 | 942.75132 | 924.71984 | [M+NH_4_]^+^, ([M+K]^+^, [M+Na]^+^) | TG 16:0_20:5_22:6 | C_61_H_96_O_6_ | 692.5 | -3.608 | 1.9 |
| 3.07 | 315.7 | 1.552 | 870.75322 | 852.71756 | [M+NH_4_]^+^, ([M+Na]^+^, [M+K]^+^) | TG 16:1_18:2_18:2 | C_55_H_96_O_6_ | 865.7 | -1.521 | 2.6 |
| 3.01 | 313.9 | 1.543 | 868.7365 | 850.70241 | [M+NH_4_]^+^, ([M+Na]^+^, [M+K]^+^) | TG 16:1_18:2_18:3 | C_55_H_94_O_6_ | 921.1 | -2.628 | 2.2 |
| 2.95 | 312.4 | 1.535 | 866.71882 | 848.68401 | [M+NH_4_]^+^, ([M+Na]^+^) | TG 16:1_18:3_18:3 | C_55_H_92_O_6_ | 624.3 | -4.546 | 0.1 |
| 3.25 | 324.7 | 1.597 | 890.81487 | 872.78047 | [M+NH_4_]^+^, ([M+Na]^+^) | TG 17:0_18:1_18:1 | C_56_H_104_O_6_ | 907.6 | -2.5 | 1.7 |
| 3.20 | 322.2 | 1.584 | 888.79983 | 870.76601 | [M+NH_4_]^+^ | TG 17:0_18:1_18:2 | C_56_H_102_O_6_ | 867.1 | -1.846 | 2.5 |
| 3.22 | 326.7 | 1.607 | 914.81446 | 896.78063 | [M+NH_4_]^+^ | TG 17:0_18:1_20:3 | C_58_H_104_O_6_ | 564.0 | -3.321 | 2.4 |
| 3.16 | 321.0 | 1.578 | 886.78429 | 868.75046 | [M+NH_4_]^+^ | TG 17:1_18:1_18:2 | C_56_H_100_O_6_ | 883.2 | -1.819 | 2.8 |
| 3.13 | 324.5 | 1.596 | 910.78122 | 892.7474 | [M+NH_4_]^+^ | TG 17:1_18:1_20:4 | C_58_H_100_O_6_ | 610.8 | -4.414 | 2.5 |
| 3.33 | 330.0 | 1.623 | 906.84776 | 888.81394 | [M+NH_4_]^+^ | TG 18:0_18:0_18:1 | C_57_H_108_O_6_ | 955.6 | -0.623 | 2.7 |
| 3.28 | 327.5 | 1.611 | 904.83229 | 886.79742 | [M+NH_4_]^+^, ([M+Na]^+^, [M+K]^+^) | TG 18:0_18:1_18:1 | C_57_H_106_O_6_ | 395.4 | -0.443 | 0.5 |
| 3.24 | 325.8 | 1.602 | 902.81639 | 884.78171 | [M+NH_4_]^+^, ([M+K]^+^, [M+Na]^+^) | TG 18:1_18:1_18:1 | C_57_H_104_O_6_ | 929.2 | -0.77 | 2.4 |
| 3.18 | 323.6 | 1.591 | 900.80022 | 882.76639 | [M+NH_4_]^+^ | TG 18:1_18:1_18:2 | C_57_H_102_O_6_ | 945.2 | -1.362 | 2.8 |
| 3.28 | 331.2 | 1.629 | 930.8482 | 912.81352 | [M+NH_4_]^+^, ([M+Na]^+^) | TG 18:1_18:1_20:1 | C_59_H_108_O_6_ | 871.2 | -1.085 | 2.6 |
| 3.16 | 327.4 | 1.611 | 924.79881 | 906.76357 | [M+NH_4_]^+^, ([M+Na]^+^, [M+K]^+^) | TG 18:1_18:1_20:4 | C_59_H_102_O_6_ | 756.4 | -2.929 | 2.4 |
| 3.20 | 330.9 | 1.629 | 950.81018 | 932.77636 | [M+NH_4_]^+^ | TG 18:1_18:1_22:5 | C_61_H_104_O_6_ | 835.2 | -4.086 | 2.8 |
| 3.14 | 329.7 | 1.623 | 948.79921 | 930.76538 | [M+NH_4_]^+^ | TG 18:1_18:1_22:6 | C_61_H_102_O_6_ | 717.5 | -1.822 | 2.9 |
| 3.13 | 321.6 | 1.582 | 898.78408 | 880.74761 | [M+NH_4_]^+^, ([M+K]^+^, [M+Na]^+^) | TG 18:1_18:2_18:2 | C_57_H_100_O_6_ | 942.0 | -1.815 | 2.3 |
| 3.12 | 325.6 | 1.602 | 922.78344 | 904.74961 | [M+NH_4_]^+^ | TG 18:1_18:2_20:4 | C_59_H_100_O_6_ | 769.0 | -2.423 | 2.4 |
| 3.07 | 324.4 | 1.596 | 920.76816 | 902.73434 | [M+NH_4_]^+^ | TG 18:1_18:2_20:5 | C_59_H_98_O_6_ | 602.7 | -2.028 | 2.5 |
| 3.08 | 328.1 | 1.615 | 946.78352 | 928.7494 | [M+NH_4_]^+^, ([M+Na]^+^, [M+K]^+^) | TG 18:1_18:2_22:6 | C_61_H_100_O_6_ | 757.1 | -2.538 | 2.7 |
| 3.15 | 333.1 | 1.640 | 974.81296 | 956.77913 | [M+NH_4_]^+^ | TG 18:1_20:3_22:5 | C_63_H_104_O_6_ | 767.7 | -3.578 | 2.9 |
| 3.06 | 330.9 | 1.629 | 970.78191 | 952.74808 | [M+NH_4_]^+^ | TG 18:1_20:4_22:6 | C_63_H_100_O_6_ | 788.8 | -3.922 | 2.9 |
| 3.00 | 329.0 | 1.619 | 968.76892 | 950.73477 | [M+NH_4_]^+^, ([M+K]^+^, [M+Na]^+^) | TG 18:1_20:5_22:6 | C_63_H_98_O_6_ | 730.8 | -1.569 | 0.8 |
| 3.08 | 319.8 | 1.572 | 896.76882 | 878.73464 | [M+NH_4_]^+^, ([M+Na]^+^, [M+K]^+^) | TG 18:2_18:2_18:2 | C_57_H_98_O_6_ | 952.1 | -1.452 | 2.7 |
| 3.02 | 317.8 | 1.563 | 894.75209 | 876.71864 | [M+NH_4_]^+^, ([M+K]^+^, [M+Na]^+^) | TG 18:2_18:2_18:3 | C_57_H_96_O_6_ | 868.3 | -2.511 | 2.7 |
| 3.02 | 325.9 | 1.604 | 944.77065 | 926.73543 | [M+NH_4_]^+^, ([M+K]^+^, [M+Na]^+^) | TG 18:2_18:2_22:6 | C_61_H_98_O_6_ | 669.7 | 0.5 | 0.2 |
| 3.00 | 321.6 | 1.582 | 918.75152 | 900.7177 | [M+NH_4_]^+^ | TG 18:2_18:3_20:4 | C_59_H_96_O_6_ | 709.9 | -3.136 | 0.2 |
| 2.93 | 319.1 | 1.570 | 916.73549 | 898.70131 | [M+NH_4_]^+^, ([M+K]^+^) | TG 18:2_18:3_20:5 | C_59_H_94_O_6_ | 849.5 | -3.049 | 2.4 |

**Supplementary Table 7**

List of identified metabolites. RT: retention time; m/z meas.: mass to charge measured; M meas.: measured mass. Primary ion has been used for normalisation and additional adducts, if detected, were reported in round brackets.

| **RT [min]** | **m/z meas.** | **M meas.** | **Ions** | **Name** | **Molecular Formula** | **Δm/z [ppm]** | **MS/MS score** |
| --- | --- | --- | --- | --- | --- | --- | --- |
| 5.36 | 146.08116 | 145.07389 | [M+H]^+^ | Acetamidobutanoic acid | C_6_H_11_NO_3_ | -0.05 | 295.0 |
| 5.92 | 217.12967 | 216.12239 | [M+H]^+^ | Acetylarginine | C_8_H_16_N_4_O_3_ | 0.704 | 836.1 |
| 5.63 | 204.12327 | 203.116 | [M+H]^+^ | Acetyl-Carnitine | C_9_H_17_NO_4_ | 1.103 | 998.6 |
| 6.33 | 189.12364 | 188.11636 | [M+H]^+^ | Acetyllysine | C_8_H_16_N_2_O_3_ | 1.415 | 997.8 |
| 2.18 | 346.05597 | 347.06324 | [M-H]^-^ | Adenosine Monophosphate | C_10_H_14_N_5_O_7_P | 0.459 | 515.5 |
| 7.24 | 189.12359 | 188.11631 | [M+H]^+^ | Ala-Val | C_8_H_16_N_2_O_3_ | 1.152 | 505.7 |
| 4.88 | 132.10191 | 131.09471 | [M+H]^+^, ([M+Na]^+^) | Alloisoleucine | C_6_H_13_NO_2_ | 0.076 | 998.2 |
| 1.03 | 297.2425 | 296.23522 | [M+H]^+^ | Alpha-dimorphecolic acid | C_18_H_32_O_3_ | 0.284 | 481.3 |
| 1.74 | 145.01427 | 146.02155 | [M-H]^-^ | Alpha-Ketoglutaric acid | C_5_H_6_O_5_ | 0.156 | 987.8 |
| 4.95 | 114.05489 | 131.0582 | [M+H-H_2_O]^+^ | Aminolevulinate | C_5_H_9_NO_3_ | -0.534 | 677.5 |
| 5.96 | 369.17407 | 370.18135 | [M-H]^-^ | Androsterone sulfate | C_19_H_30_O_5_S | -0.137 | 527.9 |
| 7.75 | 175.11902 | 174.11174 | [M+H]^+^ | Arginine | C_6_H_14_N_4_O_2_ | 0.421 | 999.8 |
| 6.48 | 291.12973 | 290.12245 | [M+H]^+^ | Argininosuccinic acid | C_10_H_18_N_4_O_6_ | -0.63 | 715.6 |
| 6.05 | 133.06088 | 132.05358 | [M+H]^+^, ([M+Na]^+^) | Asparagine | C_4_H_8_N_2_O_3_ | 0.729 | 998.1 |
| 1.89 | 147.07666 | 146.06939 | [M+H]^+^ | Asparagine methyl ester | C_5_H_10_N_2_O_3_ | 1.662 | 508.1 |
| 4.90 | 232.15456 | 231.14728 | [M+H]^+^ | Butyryl carnitine | C_11_H_21_NO_4_ | 0.998 | 876.6 |
| 6.46 | 162.11262 | 161.10534 | [M+H]^+^ | Carnitine | C_7_H_15_NO_3_ | 0.931 | 899.8 |
| 1.89 | 147.02993 | 148.03721 | [M-H]^-^ | Citramalic acid | C_5_H_8_O_5_ | 0.251 | 448.8 |
| 2.31 | 191.01981 | 192.02708 | [M-H]^-^ | Citrate | C_6_H_8_O_7_ | 0.433 | 998.0 |
| 6.22 | 176.10318 | 175.09575 | [M+H]^+^, ([M+H-H_2_O]^+^, [M+K]^+^) | Citrulline | C_6_H_13_N_3_O_3_ | 1.199 | 994.7 |
| 5.02 | 239.09099 | 240.09827 | [M-H]^-^ | CMPF | C_12_H_16_O_5_ | -4.5 | 979.3 |
| 5.89 | 132.07673 | 131.0697 | [M+H]^+^, ([M+H-H_2_O]^+^) | Creatine | C_4_H_9_N_3_O_2_ | -0.158 | 915.7 |
| 4.46 | 114.06612 | 113.05889 | [M+H]^+^, ([M+Na]^+^) | Creatinine | C_4_H_7_N_3_O | -0.474 | 871.1 |
| 1.02 | 211.14436 | 210.13708 | [M+H]^+^ | Cyclo(Leu-Pro) | C_11_H_18_N_2_O_2_ | 1.229 | 936.5 |
| 1.04 | 223.07495 | 222.06767 | [M+H]^+^ | Cystathionine | C_7_H_14_N_2_O_4_S | 1.022 | 861.0 |
| 5.39 | 367.15842 | 368.16569 | [M-H]^-^ | Dehydroisoandrosterone sulfate | C_19_H_28_O_5_S | -0.139 | 1000 |
| 9.93 | 391.2854 | 392.29268 | [M-H]^-^ | Deoxycholic acid | C_24_H_40_O_4_ | 0.069 | 645.9 |
| 2.49 | 115.05019 | 114.04292 | [M+H]^+^ | Dihydrouracil | C_4_H_6_N_2_O_2_ | -0.096 | 660.3 |
| 4.81 | 154.084 | 153.0767 | [M+H]^+^ | Dopamine | C_8_H_11_NO_2_ | 5.0 | 862.0 |
| 5.81 | 143.0815 | 142.07423 | [M+H]^+^ | Ectoine | C_6_H_10_N_2_O_2_ | -0.001 | 948.7 |
| 1.36 | 289.21643 | 288.20916 | [M+H]^+^ | Epitestosterone | C_19_H_28_O_2_ | 0.784 | 577.4 |
| 4.11 | 297.24242 | 296.23515 | [M+H]^+^ | Epoxyoctadecenoic acid | C_18_H_32_O_3_ | 0.233 | 460.5 |
| 1.24 | 295.22696 | 294.21968 | [M+H]^+^ | FA 18:3+1O | C_18_H_30_O_3_ | 0.711 | 239.1 |
| 7.28 | 309.20725 | 310.21453 | [M-H]^-^ | FA 18:3+2O | C_18_H_30_O_4_ | 0.414 | 710.4 |
| 4.78 | 146.05998 | 145.0527 | [M+H]^+^ | Formylindole | C_9_H_7_NO | -0.477 | 990.3 |
| 0.99 | 195.05112 | 196.0584 | [M-H]^-^ | Gluconate | C_6_H_12_O_7_ | 0.494 | 953.0 |
| 5.35 | 180.08686 | 179.07953 | [M+H]^+^, ([M+H-H_2_O]^+^) | Glucosamine | C_6_H_13_NO_5_ | 1.222 | 243.2 |
| 0.96 | 146.04586 | 147.05314 | [M-H]^-^ | Glutamic acid | C_5_H_9_NO_4_ | -0.131 | 981.7 |
| 5.98 | 147.0764 | 146.0694 | [M+H]^+^, ([M+Na]^+^, [M+K]^+^) | Glutamine | C_5_H_10_N_2_O_3_ | -0.313 | 999.9 |
| 1.80 | 89.0244 | 90.03167 | [M-H]^-^, ([M-H-H_2_O]^-^) | Glyceraldehyde | C_3_H_6_O_3_ | -0.159 | 556.8 |
| 1.09 | 105.01925 | 106.02653 | [M-H]^-^ | Glycerate | C_3_H_6_O_4_ | -0.767 | 999.1 |
| 6.66 | 258.11027 | 258.1106 | [M]^+^ | Glycerophosphocholine | C_8_H_21_NO_6_P | 0.698 | 925.0 |
| 6.03 | 118.06139 | 117.05411 | [M+H]^+^ | Glycocyamine | C_3_H_7_N_3_O_2_ | 2.003 | 1000 |
| 5.95 | 448.30679 | 449.31406 | [M-H]^-^ | Glycoursodeoxycholic acid | C_26_H_43_NO_5_ | -0.133 | 699.9 |
| 4.61 | 260.18584 | 259.17856 | [M+H]^+^ | Hexanoyl-Carnitine | C_13_H_25_NO_4_ | 0.766 | 998.4 |
| 4.66 | 258.17022 | 257.16294 | [M+H]^+^ | Hexenoylcarnitine | C_13_H_23_NO_4_ | 0.827 | 911.3 |
| 6.10 | 156.07683 | 155.06888 | [M+H]^+^, ([M+H-H_2_O]^+^, [M+Na]^+^) | Histidine | C_6_H_9_N_3_O_2_ | 0.522 | 947.5 |
| 7.86 | 311.22285 | 312.23013 | [M-H]^-^ | HPODE | C_18_H_32_O_4_ | 0.237 | 866.8 |
| 2.98 | 103.04006 | 104.04731 | [M-H]^-^, ([M-H-H_2_O]^-^) | Hydroxybutyric acid | C_4_H_8_O_3_ | -0.096 | 240.6 |
| 5.60 | 248.1494 | 247.14213 | [M+H]^+^ | Hydroxybutyrylcarnitine | C_11_H_21_NO_5_ | 0.67 | 919.8 |
| 1.15 | 217.18015 | 216.17287 | [M+H]^+^ | Hydroxydodecanoic acid | C_12_H_24_O_3_ | 1.525 | 311.7 |
| 4.72 | 192.06572 | 191.05844 | [M+H]^+^ | Hydroxyindoleacetic acid | C_10_H_9_NO_3_ | 1.036 | 920.9 |
| 4.46 | 411.18465 | 412.19193 | [M-H]^-^ | Hydroxypregnenolone sulfate | C_21_H_32_O_6_S | -0.084 | 391.3 |
| 6.33 | 132.06549 | 131.05821 | [M+H]^+^ | Hydroxyproline | C_5_H_9_NO_3_ | -0.218 | 981.9 |
| 6.41 | 178.08665 | 177.07938 | [M+H]^+^ | Hydroxytryptophol | C_10_H_11_NO_2_ | 2.24 | 370.0 |
| 3.20 | 117.05574 | 118.06302 | [M-H]^-^ | Hydroxyvaleric acid | C_5_H_10_O_3_ | 0.244 | 992.9 |
| 2.56 | 135.03118 | 136.03845 | [M-H]^-^ | Hypoxanthine | C_5_H_4_N_4_O | -0.431 | 999.8 |
| 5.38 | 141.06589 | 140.05861 | [M+H]^+^ | Imidazolepropionic acid | C_6_H_8_N_2_O_2_ | 0.24 | 310.0 |
| 4.79 | 118.06538 | 117.0581 | [M+H]^+^ | Indole | C_8_H_7_N | 1.681 | 947.8 |
| 2.92 | 190.08642 | 189.07911 | [M+H]^+^, ([M+H-H_2_O]^+^, [M+Na]^+^) | Indolepropionic acid | C_11_H_11_NO_2_ | 0.856 | 995.8 |
| 3.43 | 212.00236 | 213.00964 | [M-H]^-^ | Indoxyl sulfate | C_8_H_7_NO_4_S | 0.277 | 995.0 |
| 1.13 | 191.01978 | 192.02704 | [M-H]^-^, ([M-H-H_2_O]^-^) | Isocitric acid | C_6_H_8_O_7_ | 0.306 | 988.4 |
| 5.38 | 160.09678 | 159.08951 | [M+H]^+^ | Isovalerylglycine | C_7_H_13_NO_3_ | 0.13 | 299.9 |
| 4.72 | 209.09242 | 208.08514 | [M+H]^+^ | Kynurenine | C_10_H_12_N_2_O_3_ | 1.572 | 942.9 |
| 1.20 | 281.2478 | 280.2405 | [M+H]^+^, ([M+H-H_2_O]^+^) | Linoleic acid | C_18_H_32_O_2_ | 1.051 | 851.9 |
| 1.22 | 279.2321 | 278.22482 | [M+H]^+^ | Linolenic acid | C_18_H_30_O_2_ | 0.877 | 841.1 |
| 8.12 | 147.11286 | 146.10558 | [M+H]^+^ | Lysine | C_6_H_14_N_2_O_2_ | 0.198 | 892.9 |
| 1.13 | 133.01418 | 134.02149 | [M-H]^-^, ([M-H-H_2_O]^-^) | Malic acid | C_4_H_6_O_5_ | -0.469 | 969.7 |
| 5.89 | 248.11291 | 247.10564 | [M+H]^+^ | Malonylcarnitine | C_10_H_17_NO_6_ | 0.199 | 910.7 |
| 4.98 | 150.05839 | 149.05112 | [M+H]^+^ | Methionine | C_5_H_11_NO_2_S | 0.49 | 845.7 |
| 4.96 | 192.10208 | 191.09481 | [M+H]^+^ | Methoxytryptophol | C_11_H_13_NO_2_ | 0.93 | 279.3 |
| 6.24 | 126.10251 | 125.09524 | [M+H]^+^ | Methylistamine | C_6_H_11_N_3_ | -0.465 | 422.5 |
| 2.98 | 129.01929 | 130.02657 | [M-H]^-^, ([M-H-H_2_O]^-^) | Methylmaleate | C_5_H_6_O_4_ | -0.328 | 437.0 |
| 1.6 | 153.06595 | 152.05867 | [M+H]^+^ | Methyl-pyridone-carboxamide | C_7_H_8_N_2_O_2_ | 0.655 | 443.9 |
| 2.40 | 167.05645 | 166.04917 | [M+H]^+^ | Methylxanthine | C_6_H_6_N_4_O_2_ | 0.595 | 978.3 |
| 3.34 | 149.08088 | 148.07361 | [M+H]^+^ | Mevalonic acid | C_6_H_12_O_4_ | 0.323 | 311.1 |
| 1.74 | 116.03544 | 117.04271 | [M-H]^-^ | N-Acetylglycine | C_4_H_7_NO_3_ | 1.039 | 1000 |
| 4.47 | 302.23278 | 301.22551 | [M+H]^+^ | Nonanoylcarnitine | C_16_H_31_NO_4_ | 0.653 | 991.6 |
| 1.26 | 277.21644 | 276.20912 | [M+H]^+^, ([M+H-H_2_O]^+^) | Norandrosterone | C_18_H_28_O_2_ | 0.837 | 483.1 |
| 8.6 | 313.23859 | 314.24586 | [M-H]^-^ | Octadecanedioic acid | C_18_H_34_O_4_ | 0.485 | 472.0 |
| 4.5 | 288.21683 | 287.20956 | [M+H]^+^ | Octanoyl-Carnitine | C_15_H_29_NO_4_ | -0.192 | 998.7 |
| 4.54 | 286.20146 | 285.19418 | [M+H]^+^ | Octenoylcarnitine | C_15_H_27_NO_4_ | 0.59 | 993.9 |
| 0.87 | 282.27949 | 281.27222 | [M+H]^+^ | Oleamide | C_18_H_35_NO | 1.192 | 907.0 |
| 4.37 | 426.35829 | 425.35102 | [M+H]^+^ | Oleoyl-L-Carnitine | C_25_H_47_NO_4_ | 1.19 | 990.1 |
| 6.23 | 133.09721 | 132.08993 | [M+H]^+^ | Ornithine | C_5_H_12_N_2_O_2_ | 0.115 | 997.6 |
| 11.15 | 255.23311 | 256.24039 | [M-H]^-^ | Palmitic acid | C_16_H_32_O_2_ | 0.643 | 540.0 |
| 0.90 | 298.27438 | 297.2671 | [M+H]^+^ | Palmitoleoyl ethanolamide | C_18_H_35_NO_2_ | 1.048 | 685.0 |
| 4.38 | 400.34259 | 399.33532 | [M+H]^+^ | Palmitoyl-Carnitine | C_23_H_45_NO_4_ | 1.187 | 998.3 |
| 3.75 | 187.00707 | 188.01435 | [M-H]^-^ | p-Cresol sulfate | C_7_H_8_O_4_S | 0.092 | 711.1 |
| 5.16 | 136.07569 | 135.06842 | [M+H]^+^ | Phenylacetamide | C_8_H_9_NO | -0.008 | 962.4 |
| 3.27 | 263.10394 | 264.11122 | [M-H]^-^ | Phenylacetylglutamine | C_13_H_16_N_2_O_4_ | 0.83 | 996.4 |
| 4.71 | 166.08639 | 165.07911 | [M+H]^+^ | Phenylalanine | C_9_H_11_NO_2_ | 0.791 | 907.6 |
| 6.55 | 395.18969 | 396.19697 | [M-H]^-^ | Pregnenolone sulfate | C_21_H_32_O_5_S | -0.205 | 1000 |
| 5.59 | 116.07053 | 115.06325 | [M+H]^+^ | Proline | C_5_H_9_NO_2_ | -0.605 | 999.7 |
| 6.31 | 229.11859 | 228.11131 | [M+H]^+^ | Prolylhydroxyproline | C_10_H_16_N_2_O_4_ | 1.313 | 981.1 |
| 1.95 | 243.06249 | 244.06976 | [M-H]^-^ | Pseudouridine | C_9_H_12_N_2_O_6_ | 0.93 | 990.9 |
| 1.25 | 184.06064 | 183.0534 | [M+H]^+^, ([M+H-H_2_O]^+^) | Pyridoxic acid | C_8_H_9_NO_4_ | 1.116 | 892.4 |
| 5.97 | 130.04982 | 129.04261 | [M+H]^+^, ([M+Na]^+^) | Pyroglutamic acid | C_5_H_7_NO_3_ | -0.346 | 999.8 |
| 1.01 | 209.03036 | 210.03763 | [M-H]^-^ | Saccharic acid | C_6_H_10_O_8_ | 0.32 | 814.5 |
| 6.31 | 106.04984 | 105.04256 | [M+H]^+^ | Serine | C_3_H_7_NO_3_ | -0.287 | 971.7 |
| 4.92 | 177.1024 | 176.09512 | [M+H]^+^ | Serotonin | C_10_H_12_N_2_O | 0.894 | 965.3 |
| 6.86 | 380.25704 | 381.26431 | [M-H]^-^ | Sphinganine 1-phosphate | C_18_H_40_NO_5_P | -0.254 | 671.1 |
| 6.61 | 378.24141 | 379.24868 | [M-H]^-^ | Sphingosine 1-phosphate | C_18_H_38_NO_5_P | -0.173 | 693.5 |
| 5.74 | 126.02189 | 125.01461 | [M+H]^+^, ([M+H-H_2_O]^+^) | Taurine | C_2_H_7_NO_3_S | -0.402 | 889.0 |
| 5.96 | 257.17602 | 258.18329 | [M-H]^-^ | Tetradecanedioic acid | C_14_H_26_O_4_ | 0.7 | 611.1 |
| 1.01 | 135.02984 | 136.03712 | [M-H]^-^ | Threonic acid | C_4_H_8_O_5_ | -0.383 | 964.7 |
| 4.81 | 244.15455 | 243.14727 | [M+H]^+^ | Tiglylcarnitine | C_12_H_21_NO_4_ | 0.903 | 897.9 |
| 4.78 | 205.09733 | 204.09005 | [M+H]^+^ | Tryptophan | C_11_H_12_N_2_O_2_ | 0.961 | 988.3 |
| 2.98 | 180.06666 | 181.07394 | [M-H]^-^ | Tyrosine | C_9_H_11_NO_3_ | 0.236 | 978.3 |
| 2.37 | 167.02106 | 168.02834 | [M-H]^-^ | Urate | C_5_H_4_N_4_O_3_ | 0.006 | 993.2 |
| 2.98 | 243.0624 | 244.06967 | [M-H]^-^ | Uridine | C_9_H_12_N_2_O_6_ | 0.579 | 890.6 |
| 4.74 | 246.17023 | 245.16295 | [M+H]^+^ | Valerylcarnitine | C_12_H_23_NO_4_ | 0.996 | 977.9 |
| 1.60 | 116.07175 | 117.07903 | [M-H]^-^ | Valine | C_5_H_11_NO_2_ | 0.408 | 919.3 |

**Supplementary Table 8**

*SO-CovSel-LDA models utilising all possible combinations of the four blocks (lipidomics positive; lipidomics negative; metabolomics positive; metabolomics negative) for sCHD (n=157) and control (n=162) groups. Modality shows order of blocks in model. Correct classification accuracy shown as %. Selected models illustrated in table 3 are shaded in green.* Met ESI^+^ *– metabolomics positive;* Met ESI^-^ *– metabolomics negative;* Lip ESI^-^ *– lipidomics negative;* Lip ESI^+^ *– lipidomics positive; sCHD – structural CHD.*

| **Modality** | **Cross Validation Accuracy (%)** | **Number of Variables**  **Block 1** | **Number of Variables**  **Block 2** | **Number of Variables**  **Block 3** | **Number of Variables**  **Block 4** |
| --- | --- | --- | --- | --- | --- |
| Lip ESI^+^ | 71.43% | 6 | / | / | / |
| Lip ESI^-^ | 74.11% | 5 | / | / | / |
| Met ESI^+^ | 91.96% | 3 | / | / | / |
| Met ESI^-^ | 84.82% | 6 | / | / | / |
| Lip ESI^+^; Lip ESI^-^ | 76.79% | 6 | 4 | / | / |
| Lip ESI^+^; Met ESI^+^ | 94.64% | 1 | 4 | / | / |
| Lip ESI^+^; Met ESI^-^ | 87.05% | 4 | 6 | / | / |
| Lip ESI^-^; Lip ESI^+^ | 75.89% | 5 | 1 | / | / |
| Lip ESI^-^; Met ESI^+^ | 95.54% | 1 | 5 | / | / |
| Lip ESI^-^; Met ESI^-^ | 86.61% | 1 | 6 | / | / |
| Met ESI^+^; Lip ESI^+^ | 95.09% | 3 | 1 | / | / |
| Met ESI^+^; Lip ESI^-^ | 95.98% | 4 | 1 | / | / |
| Met ESI^+^; Met ESI^-^ | 95.98% | 3 | 5 | / | / |
| Met ESI^-^; Lip ESI^+^ | 85.71% | 5 | 2 | / | / |
| Met ESI^-^; Lip ESI^-^ | 86.16% | 4 | 3 | / | / |
| Met ESI^-^; Met ESI^+^ | 94.20% | 4 | 2 | / | / |
| Lip ESI^+^; Lip ESI^-^; Met ESI^+^ | 95.98% | 1 | 3 | 4 | / |
| Lip ESI^+^; Lip ESI^-^; Met ESI^-^ | 88.84% | 1 | 6 | 6 | / |
| Lip ESI^+^; Met ESI^+^; Lip ESI^-^ | 95.98% | 0 | 4 | 1 | / |
| Lip ESI^+^; Met ESI^+^; Met ESI^-^ | 96.88% | 1 | 3 | 5 | / |
| Lip ESI^+^; Met ESI^-^; Lip ESI^-^ | 91.52% | 1 | 6 | 5 | / |
| Lip ESI^+^; Met ESI^-^; Met ESI^+^ | 95.98% | 1 | 1 | 3 | / |
| Lip ESI^-^; Lip ESI^+^; Met ESI^+^ | 95.54% | 1 | 0 | 5 | / |
| Lip ESI^-^; Lip ESI^+^; Met ESI^-^ | 88.84% | 1 | 5 | 5 | / |
| Lip ESI^-^; Met ESI^+^; Lip ESI^+^ | 95.54% | 1 | 5 | 0 | / |
| Lip ESI^-^; Met ESI^+^; Met ESI^-^ | 96.88% | 1 | 3 | 6 | / |
| Lip ESI^-^; Met ESI^-^; Lip ESI^+^ | 87.50% | 1 | 6 | 2 | / |
| Lip ESI^-^; Met ESI^-^; Met ESI^+^ | 96.43% | 3 | 6 | 6 | / |
| Met ESI^+^; Lip ESI^+^; Lip ESI^-^ | 95.98% | 4 | 0 | 1 | / |
| Met ESI^+^; Lip ESI^+^; Met ESI^-^ | 96.88% | 3 | 1 | 5 | / |
| Met ESI^+^; Lip ESI^-^; Lip ESI^+^ | 95.98% | 4 | 1 | 0 | / |
| Met ESI^+^; Lip ESI^-^; Met ESI^-^ | 97.32% | 4 | 1 | 6 | / |
| Met ESI^+^; Met ESI^-^; Lip ESI^+^ | 95.98% | 3 | 5 | 0 | / |
| Met ESI^+^; Met ESI^-^; Lip ESI^-^ | 96.88% | 3 | 3 | 2 | / |
| Met ESI^-^; Lip ESI^+^; Lip ESI^-^ | 89.29% | 6 | 1 | 4 | / |
| Met ESI^-^; Lip ESI^+^; Met ESI^+^ | 96.43% | 5 | 5 | 3 | / |
| Met ESI^-^; Lip ESI^-^; Lip ESI^+^ | 87.50% | 5 | 1 | 5 | / |
| Met ESI^-^; Lip ESI^-^; Met ESI^+^ | 96.88% | 2 | 1 | 4 | / |
| Met ESI^-^; Met ESI^+^; Lip ESI^+^ | 96.43% | 6 | 6 | 2 | / |
| Met ESI^-^; Met ESI^+^; Lip ESI^-^ | 96.43% | 5 | 4 | 1 | / |
| Lip ESI^+^; Lip ESI^-^; Met ESI^+^; Met ESI^-^ | 96.88% | 1 | 0 | 3 | 5 |
| Lip ESI^+^; Lip ESI^-^; Met ESI^-^; Met ESI^+^ | 96.88% | 1 | 2 | 4 | 4 |
| Lip ESI^+^; Met ESI^+^; Lip ESI^-^; Met ESI^-^ | 97.32% | 0 | 4 | 1 | 6 |
| Lip ESI^+^; Met ESI^+^; Met ESI^-^; Lip ESI^-^ | 96.88% | 0 | 3 | 3 | 2 |
| Lip ESI^+^; Met ESI^-^; Lip ESI^-^; Met ESI^+^ | 96.88% | 0 | 2 | 1 | 4 |
| Lip ESI^+^; Met ESI^-^; Met ESI^+^; Lip ESI^-^ | 96.43% | 1 | 4 | 2 | 1 |
| Lip ESI^-^; Lip ESI^+^; Met ESI^+^; Met ESI^-^ | 96.88% | 0 | 1 | 3 | 5 |
| Lip ESI^-^; Lip ESI^+^; Met ESI^-^; Met ESI^+^ | 96.88% | 1 | 6 | 5 | 2 |
| Lip ESI^-^; Met ESI^+^; Lip ESI^+^; Met ESI^-^ | 96.88% | 0 | 3 | 1 | 5 |
| Lip ESI^-^; Met ESI^+^; Met ESI^-^; Lip ESI^+^ | 96.88% | 1 | 3 | 6 | 0 |
| Lip ESI^-^; Met ESI^-^; Lip ESI^+^; Met ESI^+^ | 96.43% | 0 | 5 | 5 | 3 |
| Lip ESI^-^; Met ESI^-^; Met ESI^+^; Lip ESI^+^ | 96.88% | 3 | 5 | 5 | 1 |
| Met ESI^+^; Lip ESI^+^; Lip ESI^-^; Met ESI^-^ | 97.32% | 4 | 0 | 1 | 6 |
| Met ESI^+^; Lip ESI^+^; Met ESI^-^; Lip ESI^-^ | 96.88% | 3 | 0 | 3 | 2 |
| Met ESI^+^; Lip ESI^-^; Lip ESI^+^; Met ESI^-^ | 97.32% | 4 | 1 | 0 | 6 |
| Met ESI^+^; Lip ESI^-^; Met ESI^-^; Lip ESI^+^ | 97.32% | 4 | 1 | 6 | 0 |
| Met ESI^+^; Met ESI^-^; Lip ESI^+^; Lip ESI^-^ | 97.32% | 4 | 5 | 1 | 5 |
| Met ESI^+^; Met ESI^-^; Lip ESI^-^; Lip ESI^+^ | 96.88% | 3 | 3 | 2 | 0 |
| Met ESI^-^; Lip ESI^+^; Lip ESI^-^; Met ESI^+^ | 97.77% | 6 | 2 | 6 | 6 |
| Met ESI^-^; Lip ESI^+^; Met ESI^+^; Lip ESI^-^ | 96.88% | 5 | 1 | 3 | 1 |
| Met ESI^-^; Lip ESI^-^; Lip ESI^+^; Met ESI^+^ | 97.32% | 6 | 5 | 3 | 5 |
| Met ESI^-^; Lip ESI^-^; Met ESI^+^; Lip ESI^+^ | 97.32% | 5 | 1 | 6 | 2 |
| Met ESI^-^; Met ESI^+^; Lip ESI^+^; Lip ESI^-^ | 97.32% | 5 | 4 | 1 | 1 |
| Met ESI^-^; Met ESI^+^; Lip ESI^-^; Lip ESI^+^ | 97.32% | 5 | 5 | 1 | 2 |

**Supplementary Table 9**

*Metabolites and lipids identified through SO-CovSel-LDA models classifying sCHD and control groups. Analyte names with mean normalised intensity, standard deviation (SD), direction of change and P value. Intensities and SD presented to three significant figures. P values calculated on univariate analysis through Mann Whitney U Test. sCHD: structural CHD.*

|  | **HMDB Code** | **Normalised Intensity**  **sCHD**  *Mean (SD)* | **Normalised Intensity**  **Control**  *Mean (SD)* | **Direction of Change sCHD** | **Direction of Change Control** | **P value** |
| --- | --- | --- | --- | --- | --- | --- |
| **Metabolomics ESI^+^** | | | | | | |
| Taurine | HMDB0000251 | 0.003 (0.003) | 0.001 (0.001) | ↑ | ↓ | <0.0001 |
| Oleamide | HMDB0002117 | 0.199 (0.070) | 0.128 (0.055) | ↑ | ↓ | <0.0001 |
| Palmitoleoyl Ethanolamide | HMDB0013648 | 0.004 (0.004) | 0.008 (0.007) | ↓ | ↑ | <0.0001 |
| Epoxyoctadecenoic acid | HMDB0004701 | 0.001 (0.000) | 0.001 (0.000) | ↓ | ↑ | <0.0001 |
| **Metabolomics ESI^-^** | | | | | | |
| Glutamic Acid | HMDB0000148 | 0.013 (0.007) | 0.008 (0.005) | ↑ | ↓ | <0.0001 |
| Hydroxypregnenolone Sulfate | HMDB0000416 | 0.000 (0.000) | 0.001 (0.000) | ↓ | ↑ | <0.0001 |
| Hypoxanthine | HMDB0000157 | 0.002 (0.003) | 0.001 (0.000) | ↑ | ↓ | <0.0001 |
| Methylmaleate | HMDB0000634 | 0.007 (0.003) | 0.005 (0.002) | ↑ | ↓ | <0.0001 |
| Pseudouridine | HMDB0000767 | 0.001 (0.001) | 0.001 (0.001) | ↓ | ↑ | 0.5670 |
| Uridine | HMDB0000296 | 0.013 (0.005) | 0.015(0.006) | ↓ | ↑ | 0.016 |
| **Lipids ESI^-^** | | | | | | |
| PS 18:0_20:4 | HMDB0012383 | 0.812 (0.754) | 0.268 (0.247) | ↑ | ↓ | <0.0001 |

**Supplementary Table 10**

*Correlation coefficients (R) for selected metabolites and lipids by SO-CovSel-LDA to maternal age and BMI for sCHD, gCHD and control groups.*

|  | **Age Correlation Coefficient (R)** | **BMI Correlation Coefficient (R)** |
| --- | --- | --- |
| **Metabolomics Positive** | | |
| Taurine | 0.238 | 0.049 |
| Oleamide | 0.338 | 0.012 |
| Palmitoleoyl Ethanolamide | -0.272 | -0.078 |
| Epoxyoctadecenoic acid | -0.170 | 0.010 |
| **Metabolomics Negative** | | |
| Glutamic Acid | 0.213 | 0.278 |
| Hydroxypregnenolone Sulfate | -0.319 | 0.017 |
| Hypoxanthine | 0.136 | 0.047 |
| Methylmaleate | 0.219 | 0.078 |
| Pseudouridine | -0.041 | 0.101 |
| Uridine | -0.126 | 0.069 |
| **Lipids Negative** | | |
| PS 18:0_20:4 | 0.249 | -0.012 |

**Supplementary Table 11**

*PLS-DA modelling of sCHD group utilising EUROCAT and clinical classifications. Best performing model for each classification on cross-validation (CV) presented. Table A: Training, CV and Test performance for EUROCAT classification utilising metabolomics (met) in electron spray ionisation (ESI) positive (+) mode. Table B: Training, CV and Test performance for clinical classification utilising met ESI^+^ mode. VIP shows number of analytes with VIP score >1 within the model. VIP: variable important in projection; LV: number of latent variables.*

| **Table A** | **Mode** | **Analytes** | **VIP** | **LV** | **Accuracy**  **(%)** | | | | | **Test Sensitivity**  **(%)** | | | | **Test Specificity**  **(%)** | | | |
| --- | --- | --- | --- | --- | --- | --- | --- | --- | --- | --- | --- | --- | --- | --- | --- | --- | --- |
|  |  |  |  |  | *Training* | *CV* | | | *Test* | *Severe* | *Non-Severe* | | *Unclassified* | *Severe* | *Non-Severe* | | *Unclassified* |
|  | Met ESI^+^ | 74 | 21 | 5 | 97.27 | 84.52 | | | 78.72 | 82.76 | 85.71 | | 25.00 | 100.00 | 81.82 | | 90.70 |
|  | | | | | | | | | | | | | | | | | |
| **Table B** |  |  |  |  | **Accuracy**  **(%)** | | | | | **Test Sensitivity**  **(%)** | | | | **Test Specificity**  **(%)** | | | |
|  | **Mode** | **Analytes** | **VIP** | **LV** | *Training* | | *CV* | *Test* | | *Acyanotic* | | *Cyanotic* | | *Acyanotic* | | *Cyanotic* | |
|  | Met ESI^+^ | 74 | 22 | 4 | 89.09 | | 85.05 | 89.36 | | 96.88 | | 73.33 | | 73.33 | | 96.88 | |

**Supplementary Figure Captions**

Supplementary Figure 1*: Three-dimensional PCA illustrating class separation between sCHD (red) and control (blue). Hotelling (T^2^) confidence ellipses with 95% confidence level shown for each class. (a) PCA scores and loadings plots of lipidomics analyses in electron spray ionisation (ESI) positive (+) and negative (-) modes. (b) PCA scores and loadings plots of metabolomics analyses in ESI^+^ and ESI^-^ modes.*

Supplementary Figure 2*: VIP scores plot of the 20 metabolites with the highest VIP scores identified through PLS-DA utilising the metabolomics dataset for sCHD and control. sCHD: structural CHD.*

Supplementary Figure 3*: Maternal BMI and maternal age correlations of selected analytes (n=11) from So-CovSel-LDA. For each analyte (a) graph showing correlation of analyte and maternal age with correlation coefficient and p value; data from sCHD, gCHD and control groups (b) graph showing correlation of analyte and maternal BMI with correlation coefficient and p value; data from sCHD, gCHD and control groups.*
